# Molecular mechanisms and evolutionary robustness of a color switch in proteorhodopsins

**DOI:** 10.1126/sciadv.adj0384

**Published:** 2024-01-24

**Authors:** Jiafei Mao, Xinsheng Jin, Man Shi, David Heidenreich, Lynda J. Brown, Richard C. D. Brown, Moreno Lelli, Xiao He, Clemens Glaubitz

**Affiliations:** ^1^ Institute for Biophysical Chemistry and Center for Biomolecular Magnetic Resonance (BMRZ), Goethe University Frankfurt, Max von Laue Straße 9, 60438 Frankfurt am Main, Germany.; ^2^ Shanghai Engineering Research Center of Molecular Therapeutics and New Drug Development, Shanghai Frontiers Science Center of Molecule Intelligent Syntheses, School of Chemistry and Molecular Engineering, East China Normal University, Shanghai, 200062, China.; ^3^ Department of Chemistry, University of Southampton, Southampton, SO17 1BJ UK.; ^4^ Department of Chemistry “Ugo Schiff” and Magnetic Resonance Center (CERM), University of Florence, Via della Lastruccia 3, Sesto Fiorentino, 50019 Italy.; ^5^ Consorzio Interuniversitario Risonanze Magnetiche MetalloProteine (CIRMMP), Via Luigi Sacconi 6, Sesto Fiorentino, 50019 Italy.; ^6^ New York University–East China Normal University Center for Computational Chemistry, New York University Shanghai, Shanghai, 200062, China.

## Abstract

Proteorhodopsins are widely distributed photoreceptors from marine bacteria. Their discovery revealed a high degree of evolutionary adaptation to ambient light, resulting in blue- and green-absorbing variants that correlate with a conserved glutamine/leucine at position 105. On the basis of an integrated approach combining sensitivity-enhanced solid-state nuclear magnetic resonance (ssNMR) spectroscopy and linear-scaling quantum mechanics/molecular mechanics (QM/MM) methods, this single residue is shown to be responsible for a variety of synergistically coupled structural and electrostatic changes along the retinal polyene chain, ionone ring, and within the binding pocket. They collectively explain the observed color shift. Furthermore, analysis of the differences in chemical shift between nuclei within the same residues in green and blue proteorhodopsins also reveals a correlation with the respective degree of conservation. Our data show that the highly conserved color change mainly affects other highly conserved residues, illustrating a high degree of robustness of the color phenotype to sequence variation.

## Teaser

The mechanism of color switching between green and blue proteorhodopsin has been resolved in its evolutionary context.

## INTRODUCTION

Proteins have a certain capacity of adaption to evolutionary selection pressure by mutational modification of functions (or phenotypes). A fundamental yet underexplored question of protein evolution is how this ability is maintained during sequence diversification across species. Here, we have chosen the proteorhodopsin (PR) protein family, which has a high overall sequence variety, as a model system to address the molecular mechanism of such a robust mutation-induced phenotype change.

**Fig. 1.**
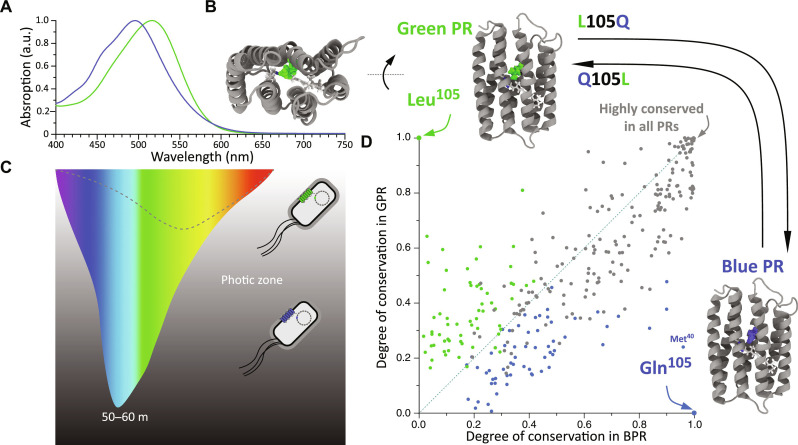
Evolution selects a single residue for tuning the color phenotype of PRs. (**A**) Within the PR family, green and blue variants are found with two distinct light absorption wavelengths at 500 and 520 nm. (**B**) The color of PRs is determined by the retinal chromophore and its protein surrounding embedded in the 7TM core. (**C**) During evolution, the PR color phenotype is selected by the predominant wavelength of ambient light at different depths of seawater. Green PRs (GPRs) in shallow water have mainly a Leu residue at position 105, while blue PRs (BPR) in deep water carry predominantly a Gln residue at this position. (**D**) For each sequence position, the probability of the most conserved residue type in GPR is plotted against the probability of the same residue type at that position in BPR (green dots) and vice versa (blue dots). Leu^105^ only occurs for GPRs but is completely excluded in BPRs. It is therefore plotted on the vertical axis (blue 0.0, green 1.0), while Gln^105^, which is present in BPRs, is plotted on the horizontal axis (blue 1.0, green 0.0). The residues conserved in all PRs are shown on the diagonal (blue 1.0, green 1.0). The analysis of 2840 diverse PR sequences suggests that no other residue in the PR family has coevolved with the color mutation. For additional ConSurf (*26*, *27*) and MISTIC2 (*28*) analysis, see figs. S22 and S23. (C) was inspired by (*66*). a.u., arbitrary units.

PRs are light-driven outward proton pumps ubiquitously found in marine microbes (*1*–*4*). Since light is their primary energy source for proton transport, their absorption characteristics and color phenotype are determined by ambient light conditions (Fig. 1, A to C). Metagenomic and biophysical data (*5*–*7*) indicate that a single residue at position 105 controls the color of PRs. Blue light reaches greater depths in water and dominates the deep photic zone. Residue 105 of PRs found in this region is highly populated with glutamine (Gln) causing a blue-shifted light absorption. In contrast, PRs found in bacteria in the upper water layers are highly likely to carry a leucine (Leu) at this position, with maximized green light absorption (Fig. 1, A to C). This blue-green color switching effect is maintained within the PR family encompassing highly diverse protein sequences (*5*). Such a phenotype switch represents a unique chance for studying the origin of robustness in protein evolution.

**Fig. 2.**
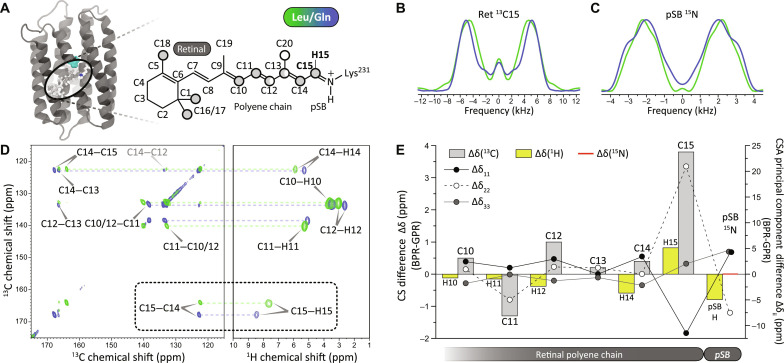
The color-determining residue Leu/Gln^105^ modulates the structure of retinal chromophore. (**A**) Leu/Gln^105^ is located near the end of the retinal polyene chain close to carbon position C15. Circles illustrate the retinal isotope labeling scheme used here (gray: ^13^C_10–18_-retinal, white: ^13^C_12,13,20_-retinal). (**B** and **C**) Recoupled ^13^C and ^15^N CSA pattern of retinal carbon C15 and pSB nitrogen in GPR and BPR. Spectral differences indicate color mutation–induced structural perturbations on these sites. (**D**) ^13^C-^13^C and ^13^C-^1^H 2D ssNMR spectra of ^13^C_10–18_-retinal in GPR and BPR. The observed ^13^C and ^1^H chemical shift changes highlight structural chromophore differences between GPR and BPR. (**E**) Summary of isotropic ^1^H, ^13^C, and ^15^N chemical shift differences (bar charts) and ^13^C-CSA and ^15^N-CSA changes (dots) between BPR and GPR. For the CSA, principal value differences are provided.

PRs are heptahelical integral membrane proteins that harbor all-trans retinal as their chromophore (Fig. 2A), which is covalently bound to Cε of the highly conserved Lys^231^ via a protonated Schiff base (pSB) linker. The general color tuning mechanism of retinal proteins, i.e., how the protein environment shifts the absorption maximum of the bound chromophore, represents a fundamental biophysical question. Several interrelated structural and physiochemical factors, such as pSB-protein interactions, retinal structure, H-bond patterns, conformation of the binding pocket, and the electrostatic potential around the chromophore, have been identified as contributing partners (*8*, *9*), and even machine learning–based approaches have been brought forward for rationalizing a sequence-based color prediction (*10*). For the specific case of the green PR (GPR)/blue PR (BPR) color switch, previous studies (*11*–*14*), including ours (*15*, *16*), have tried to explain how a simple polarity change of a single residue can cause such a large 20-nm green-to-blue color shift. Electrostatic effects (*13*, *14*) appeared as the most likely explanation, but the incomplete experimental datasets and limitations of the available three-dimensional (3D) structures required a number of theoretical assumptions that ultimately prevent final certainty of the proposed models.

The recently reported x-ray (*17*) and single particle cryo–electron microscopy (cryo-EM) structures of PRs (*18*) offer exciting mechanistic insights into the proton pumping functions and should provide a proper starting point for extensive simulation to predict their absorption color. However, these structures have been obtained at nonphysiological pH values where the color tuning effect diminishes (*11*, *15*). Notably, a quantitative understanding of color tuning as observed in PRs (Δλ = 20 nm) requires an explicit high-resolution characterization of both structures and electronic properties of the protein-chromophore complex, which remains elusive for these proteins. Quantum chemical calculations based on these structures could not quantitatively explain the color difference between GPR and BPR (*13*, *16*) without a number of assumptions (*14*).

In this work, we thoroughly address the mechanism of GPR/BPR color switching via an integrative biophysical approach combining advanced biophysical, structural, and computational techniques (text S1 and fig. S1). Solid-state nuclear magnetic resonance (ssNMR) spectroscopy, combined with automated fragmentation quantum mechanics/molecular mechanics (AF-QM/MM) (*19*), has enabled us to model the structures of GPR and BPR variants under functionally relevant conditions and to capture structural changes induced by the color switching mutation. Dynamic nuclear polarization (DNP), a powerful technique that boosts NMR sensitivity by orders of magnitude (*20*–*22*), has been extensively applied here to support the ssNMR spectroscopic mapping of the retinal chromophore and its protein environment. The AF-QM/MM approach permits accurate and efficient linear-scaling ab initio quantum chemical calculations of NMR spectroscopic parameters of large proteins such as PRs. Here, this approach has been applied for QM-level protein structural modeling with experimental ssNMR data. Our AF-QM/MM calculations are also extended by a polarized charge model, which allows exploring explicitly the contributions of the protein electrostatic field.

Our structural models derived through an integrative approach allow us to fathom the mechanism of color switching in PRs that involves synergistically coupled structural and electrostatic changes along the retinal polyene chain, the ionone ring, and within the binding pocket. We further investigated how this efficient color switching effect remains robust in the face of evolutionary diversity within the PR family. The molecular mechanisms revealed here can be used to infer color tunability in other microbial rhodopsins beyond the PR family. Our work reconciles function tunability, protein electrostatics and phenotype robustness at the interface of biophysics, quantum chemistry, and protein evolution.

## RESULTS AND DISCUSSION

### The lack of coevolutionary partner residues of the PR color switch

Previous metagenomic (*6*, *7*, *23*), biophysical (*11*, *12*, *15*), structural biology (*17*, *18*), and computational studies (*10*, *13*, *16*, *24*) suggest that the PR color switching is mainly dominated by the residue at position 105. We have initially searched potential coevolving partner residues of this color switch in PRs. To this end, we have analyzed residue patterns at each position in GPRs and BPRs from 2840 PR sequences collected in diverse environmental samples (text S21). For each residue position, we have identified the most frequently occurring residue type in the BPR group and have determined the corresponding frequency of this particular residue in the GPR group. As plotted in Fig. 1D, this analysis places the color switch residues Leu^105^ and Gln^105^ exclusively in GPR and BPR, respectively. No other residue position exhibits such a pattern, which indicates the absence of strongly coevolving residues. Residue Met^40^ appears the mostly coevolved with Gln^105^, while it diversifies drastically in GPRs. However, this residue is located in a region that is not affected by the color switch (*25*).

We have also calculated the level of conservations of each residue site using the ConSurf server (*26*, *27*), which takes into account the uneven sequence similarity and phylogenetic structure of the PR sequences. As shown in fig. S22, while comparing the evolutionary conservation score in GPRs and BPRs, a few residue sites show distinct levels of conservation. In particular, F48, L209, and S246 (residue type taken from GPR, UniProt Q9F7P4) seem more conserved in GPR than those in BPR, while K125 is more conserved in BPR. All these residues are located outside of the retinal binding pocket, on the protein surface or in the regions that are known to be unperturbed by the color switch (*15*) and are therefore unlikely to contribute to the color tuning. We have then further mined the coevolution pattern using MISTIC2 (*28*). As shown in fig. S23, no strong coevolution pattern can be discovered for the color switch residue. Only a few residues, including residue 39, 40, 65, 68, 114, and 186, show limited coevolution. Similar to the residues identified by ConSurf scores, these residues are also unlikely to contribute to the color tuning based on the structure and our previous NMR work (*15*, *18*). All these observations rule out the role of low-order epistasis in supporting the color switching function of residues at position 105.

The unambiguous role of residue 105 justifies the use of the L105Q mutation in GPR as a model for BPR. The comparative dataset described in the following was therefore recorded on GPR and its corresponding blue L105Q mutant to which we simply refer as BPR.

### The PR color switch and the pSB counterions

A widely accepted mechanistic hypothesis for color tuning of retinal proteins is based on interactions between pSB and counterions (*12*, *13*, *16*). However, our previous work has shown that the isotropic chemical shift of the pSB nitrogen is not influenced by the color switch (*15*). We therefore determined the chemical shift anisotropy (CSA) of the pSB nitrogen via R-symmetry CSA recoupling experiments (*29*) under DNP conditions (fig. S4A). The CSA reports on the anisotropic distribution of the electron density surrounding a nucleus and therefore maps comprehensively the molecular and electronic structures (*29*). The implementation of DNP in our CSA measurement has brought two improvements. First, the large DNP signal gain (about 50-fold) allows direct detection of ^15^N spins, which have low NMR sensitivity. Second, the low temperature required for DNP (ca. 110 K) suppresses protein dynamics and therefore reduces possible spectral averaging due to molecular motions. Technical details regarding the ^15^N CSA recoupling experiments can be found in text S7. As indicated by our CSA measurement (Fig. 2C; figs. S4, A and B, and S5, A and B; and table S2), the pSB nitrogen shows a slight response to the color mutation. The most affected CSA principal component (δ_22_) (Fig. 2E and table S2) aligns roughly with the pSB nitrogen–retinal C15 double bond, suggesting that the local structural change on the pSB occurs mainly toward the retinal moiety. In addition, the δ_11_ component of pSB nitrogen, which points toward the pSB proton, also undergoes observable changes. We have therefore further visualized this proton by 2D ^1^H-^15^N heteronuclear correlation (HETCOR) spectra (fig. S6 and text S8). The pSB proton shows observable ^1^H chemical shift changes (table S2). Here, both ^15^N CSA and ^1^H chemical shift data support a structural change of pSB in response to the color mutation.

To further investigate whether the observed structural change of the pSB is coupled with its counterions, namely, side-chain carboxylate groups of Asp^97^ and Asp^227^, we further mapped these moieties by NMR spectroscopy. Despite extensive efforts by our previous high-field ssNMR experiments on extensively isotope-labeled samples, the chemical shift assignment of these counterions remained unambiguous (*15*). Here, we have developed an alternative NMR approach for the direct detection of the pSB-counterion contacts via dipolar-based magnetization transfer between these sites. Establishing such a long-distance transfer between spatially distal ^15^N and ^13^C spins (4 to 5 Å) is associated with several problems, including low transfer efficiency, competition from short-distance spin pairs, loss of sensitivity with long transfer times, and radio frequency heat deposition, which were overcome by the specific labeling schemes and DNP-enhanced magic angle spinning (MAS)–NMR at low temperature. For this purpose, we prepared [U-^13^C, ^15^Nε-Lys]-PRs, in which the counterions will be ^13^C- and the pSB nitrogen ^15^N-labeled. Magnetization transfer from the pSB nitrogen to Asp^97^ and Asp^227^ was then achieved by a long double cross polarization (CP) transfer step under DNP conditions, which revealed no substantial differences between GPR and BPR (fig. S9). More experimental details are found in text S11A. Together, our NMR data show that the PR color switch induces locally restricted structural perturbations on the pSB without perturbing its counterions.

### Localized perturbations of the retinal chromophore

**Fig. 3.**
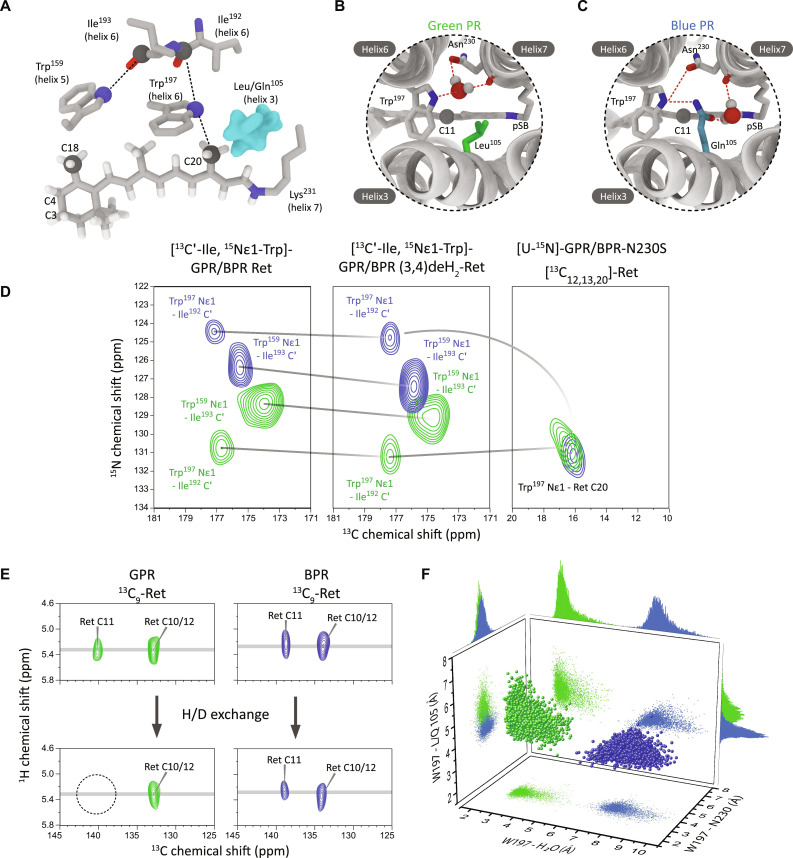
Color mutation L105Q induces structural changes in the retinal binding pocket of GPR and BPR. (**A**) Key contacts in the retinal binding pocket can be mapped by ssNMR spectroscopy (cartoon based on PDB 4JQ6). (**B** and **C**) AF-QM/MM calculations and MD simulations supported by our experimental data predict a bound water molecule at different locations in GPR and BPR, which is associated with specific structural perturbations across the retinal binding pocket [see (F) and text for details]. (**D**) The structural impact of the L105Q mutation onto residues in the retinal binding pocket is mapped via Trp^197^-Ile^192^ and Trp^159^-Ile^193^ through space cross peaks in DNP-enhanced 2D ^15^N-^13^C ssNMR spectra (left). The color mutant influences both the local Trp^197^ and the distal Trp^159^ residue in the retinal binding pocket. Both correlations are highlighted in (A). The modification of the retinal ionone ring by an additional double bond [(3,4)deH_2_-Ret] causes chemical shift changes not only at Trp^159^ but also for distal Trp^197^ in both GPR and BPR, which hints at a potential long-range effect of the color-shifting L105Q mutation (middle). Upon introducing a Asn^230^Ser mutation, the Trp^197^-Nε1 ^15^N-chemical shift in BPR changes toward a value observed in GPR, indicating that the mutation induces in BPR a local GPR-like conformation (right). The Trp^197^-Nε1 signal was detected via through-space correlation to the retinal carbon C20. (**E**) An H/D exchangeable proton signal possibly arising from bound water is detected in ^1^H-^13^C HETCOR spectra of GPR next to retinal carbon C11. In BPR, a non-exchangeable signal occurs. (**F**) MD simulation reveals distinct water locations around the retinal chromophore in GPR and BPR. Here, the water location and the local residue arrangement in MD trajectories are represented by the Trp^197^-water, Trp^197^-Leu/Gln^105^, and Trp^197^-N230 distances (shortest heavy atom distances).

As the NMR signals of the counterions do not respond to the green/blue color switch, we conclude that they are not directly involved in the PR color tuning. We have then turned to the retinal chromophore, which is conjugated to the pSB. Our previous ssNMR work has been restricted on retinal carbons C14 and C15 (*15*). To obtain a more comprehensive picture, we have explored other retinal atoms via multiple ^13^C isotope labeling schemes (Fig. 2A and table S1). The NMR signals of the retinal polyene carbons have been resolved by 2D ^13^C-^13^C proton-driven spin diffusion (PDSD) spectra (Fig. 2D, fig. S2, and text S3). Notable chemical shift changes between GPR and BPR have been observed for a number of retinal carbons (Fig. 2, and E, and table S2), indicating a propagation of structural perturbations along the retinal polyene chain beyond C14. Within all the investigated retinal polyene carbons, C15 carbon located near the color mutation residue (Figs. 2A and 3A) experiences the most drastic chemical shift change (Fig. 2E). The chemical shift perturbation diminishes gradually from carbon position C15 to C13. Beyond position C13, chemical shift perturbations reappear in an alternative pattern (Fig. 2E).

Similar to the approach on the pSB nitrogen, we have then determined the CSA of these retinal carbons (fig. S4, C and H). Here, we have first excluded potential structural effects of low DNP temperature or lipid membrane environment on retinal chromophore (text S9, A and B): As shown in fig. S7 (B and C), the retinal ^13^C chemical shifts resolved in 2D ^13^C-^13^C double-quantum (DQ)–single-quantum spectra agree well with those assigned from room temperature ssNMR spectra. In addition, a comparison of retinal ^13^C signals of PRs in synthetic lipids and in native *Escherichia coli* membranes show almost no differences (fig. S7, A and B). Therefore, we have determined the retinal carbon CSA values of PRs reconstituted in liposomes under low-temperature DNP conditions, which provides higher NMR sensitivity and reduces motional effects. The data are shown in figs. S4 and S5 and summarized in table S2 (for technical details, see text S9C). As shown in Fig. 2E, variations of ^13^C CSA principal components between GPR and BPR exhibit an alternating pattern along the polyene chain. Similar to the isotropic chemical shifts, the C13 carbon also shows only a minimal change in the CSA principal components. The most remarkable carbon CSA changes occur on the δ_22_ principal component of C15 (Fig. 2, B and E), similar to the CSA of the pSB nitrogen. The CSA δ_11_ component of this carbon, which aligns more along the C15─H15 bond (*30*), also experiences drastic changes.

Next, we have determined the retinal proton chemical shifts via high-field ^13^C-^1^H 2D correlation ssNMR spectroscopy under very-fast MAS (VFMAS) (Fig. 2D and table S2). The use of VFMAS permits to explore these ^1^H chemical shifts without protein deuteration or chemical shift rescaling (fig. S3 and table S3). The technical details regarding these experiments can be found in text S4. As shown in Fig. 2E, the most remarkable ^1^H chemical shift change has been observed on H15 that is bonded to C15 and faces the color mutation residue (Fig. 2E and fig. S3). This is followed by H14 that shows an opposite trend. Also, the chemical shift of the proton of the pSB shows a clear change (Fig. 2E, fig. S6, and text S8). In comparison, other polyene hydrogens (H10 to H12) show much smaller shift perturbations.

All of these retinal NMR data point to a structural perturbation hotspot on this moiety located near the color-tuning residue. This localized structural perturbation propagates along the polyene chain as shown by chemical shift changes. The observed alternating pattern is characteristic of a linear π-conjugated systems.

### Exploring the retinal binding pocket by DNP-enhanced ssNMR spectroscopy

The chemical shift changes observed along the retinal polyene chain suggest concomitant structural rearrangements within the retinal binding pocket. Previously, we have attempted to map such structural changes via conventional high-field ssNMR based on sequential assignment (*15*). Although our data have shown that the global folding of PR is immune to the color switching mutation (*15*), chemical shift assignment of many important residues in retinal binding pocket, in particular their side chains, has been missing. To address these challenges, we have turned to an alternative site-specific approach by combining DNP-enhanced ssNMR spectroscopy with tailored pairwise isotope labeling schemes. In general, our approach permits selective NMR detection of interesting sites at high sensitivity. The small number of NMR signals in our approach also allows evaluation of key sites in target proteins without the prerequisite of a very high spectral resolution.

We have first explored residue Lys^231^ that binds covalently to the retinal chromophore (text S11B). Its Nζ amine group exhibits a unique ^15^N chemical shift [>180 parts per million (ppm)] that is well separated from the protein backbone resonances. Therefore, the carbon NMR signal of Lys^231^ can be identified easily in 2D NζCε and Nζ(Cε)CX spectra (fig. S10). Pronounced ^13^C chemical shift changes have been observed for this residue (table S6), suggesting that the structural perturbations observed at pSB and the retinal chromophore propagate to this anchoring residue.

We have then used unique sequential pair labeling schemes to highlight selectively a number of other residues in the retinal binding pocket. Val^102^-Pro^103^ is a unique Val-Pro pair, and 2D N(CO)CX ^15^N-^13^C correlation spectra of [U-^13^C Val, ^15^N-Pro]-PRs (fig. S14 and text S11F) allow an unambiguous chemical shift assignment of both residues. Val^102^ shows a similar chemical shift change as Thr^101^ (*15*), while no effect is observed for Pro^103^. Another unique sequential pair within the retinal binding pocket is Tyr^200^-Pro^201^. ^13^C chemical shift changes have been identified on the phenol ring carbons of Tyr^200^ using this approach (fig. S11, table S6, and text S11C). The observed chemical shift changes have been confirmed by an alternative approach called Selective Detection of Internuclear Contacts by Methyl PoLarization Enhancement (SIMPLE) that we have developed previously (*25*). In this approach, the ^13^C-labeled retinal C20 methyl group has been used as an NMR “torch” to illuminate selectively its nearby Tyr^200^ residue via retinal C20-Tyr^200 13^C-^13^C polarization transfer. The ^13^C chemical shift changes of Tyr^200^ between GPR and BPR, as resolved by 1D SIMPLE ^13^C ssNMR spectra, agree well with those determined by 2D N(CO)CX experiments (fig. S11, C and D).

The abovementioned SIMPLE approach relies on through-space long-range dipolar interactions between two spins. By exploiting such long-range contacts, we have further explored other residues in retinal binding pocket that could not be selected by unique pair labeling. Such approaches rely partially on a 3D structural model as a starting point. However, as demonstrated in the next section, distinct local structural rearrangements can be explored. Here, we have mapped Trp^98^ in the retinal binding pocket via Trp Nε1-retinal carbon heteronuclear dipolar interactions in [^13^C_10–18_-ret, U-^15^N]-PRs (text S11D). This residue is located next to the pSB counterions toward the extracellular side of the retinal binding pocket. The corresponding NMR signal is masked by other signals coming from intraresidue Trp Nε1 side-chain carbon contacts in natural abundance ^13^C background (fig. S12). We have therefore included a ^13^C-^13^C DQ filter after ^15^N-^13^C dipolar recoupling scheme (fig. S12A) to suppress these ^13^C natural abundance signals (fig. S12B). This DQ filter also permits further ^13^C magnetization transfer to neighboring carbons. As shown in fig. S12D, NMR signals ascribed to Trp^98^ Nε1-retinal C14 contact can be identified using this approach, which is further confirmed by accompanied Trp^98^ Nε1-retinal C13/15 signals thanks to ^13^C14-^13^C13/15 DQ recoupling. The unperturbed Trp^98^ Nε1 chemical shifts show that structural impacts by color switch do not reach this residue, which is in line with the nearby unperturbed pSB counterions. The results obtained on these residues show that only a part of the retinal binding pocket is influenced by the color switch and that, in particular the extracellular side, is not affected.

The abovementioned observations have encouraged us to explore further the intracellular side of the retinal binding pocket (Fig. 3A), in which the color switch residue Leu/Gln^105^ is located. Trp^197^ is found next to Leu/Gln^105^ according to previous x-ray crystallography and cryo-EM structures (*17*, *18*). We have accessed this residue by correlating its indole Nε1 with nearby Ile^192^ carbonyl or retinal C20 methyl carbons (Fig. 3A) via through-space ^15^N-^13^C dipolar interactions in [^13^C′-Ile,^15^Nε-Trp]-PR samples (text S11E). In 2D ^15^N-^13^C TEDOR (transferred echo double resonance) spectra of these samples, two Trp Nε1-Ile C′ cross peaks have been detected (Fig. 3D, left). One cross peak arises, as expected, from Trp^197^ Nε1-Ile^192^ C′. The other cross peak, according to the available x-ray and cryo-EM structures, originates from Trp^159^ Nε1-Ile^193^ C′ (Fig. 3A). The peak assignment was based on mutating Ile^193^ to a valine, which occurs in some native PR sequences (fig. S13B) and further validated by visualizing the dipolar interaction between nitrogen Trp^159^ Nε1 and retinal carbon C20 (fig. S13C). Both cross peaks show remarkable differences between GPR and BPR (Fig. 3D, left). Trp^197^ Nε1 experiences a drastic chemical shift perturbation (6.3 ppm; table S6), while its nearby Ile^192^ C′ only undergoes a small change (−0.5 ppm). As Ile^192^ is located less than two α-helical turns away from Trp^197^ on the same helix, our NMR observations suggest a major local structural reorganization around the color switching residues that does not further propagate toward the intracellular direction along helix 6.

Also, Trp^159^ Nε1 experiences a substantial chemical shift change (1.9 ppm; table S6), indicating that the structural effects further propagate to this site. The nearby residue Ile^193^ C′, as visualized on the same spectra (Fig. 3D), also shows a ^13^C chemical shift change (−1.5 ppm; table S6). According to the previous x-ray structure of BPRs (*17*), Trp^159^ indole imino group forms interhelical interaction with backbone carbonyl group of Ile^193^. The opposite trend of chemical shift changes of Trp^159^ Nε1 and Ile^193^ C′ suggests that this interhelical H-bond is perturbed remotely by color switch. On helix 6, Ile^193^ is located further away from Trp^197^ than Ile^192^ (Fig. 3A). As mentioned in the previous section, the local structural perturbation on Trp^197^ does not propagate to Ile^192^. It is therefore likely that the proceeding residue Ile^193^ serves as an anchoring point for repositioning Trp^159^ indole ring upon color switch. A summary of all detected chemical shift changes in retinal binding pocket is provided in table S6.

### Local structural changes and bound water in the retinal binding pocket

Trp^197^ is highly conserved in microbial rhodopsins. To understand why the difference in the Nε1 chemical shifts between GPR and BPR is so pronounced, we have decided to predict it via automated fragmentation QM/MM (AF-QM/MM) calculations. This approach allows the prediction of chemical shift parameters of large biological macromolecules at the quantum mechanical level (for details, see text S12 and S14).

We used the BPR x-ray structure 4JQ6 (*17*) as a structural template for modeling GPR and BPR for initial chemical shift calculations and compared the structural variations between chains A and E. In addition, we included one of the best resolved 3D structures of a microbial rhodopsin, namely, bacteriorhodopsin [Protein Data Bank (PDB) 1c3w] (*31*), which contains structured water, as additional template. Only the combination of GPR structure similar to 1C3W and BPR structure similar to chain A of 4JQ6 has yielded the simulated chemical shift difference of Trp^197^ Nε1 (5.0 ppm) that could qualitatively reproduce our experimental observations (6.3 ppm; see Fig. 3D). These models differ in the side-chain orientations of Trp^197^, Leu/Gln^105^, Asn^230^, and including the position of a water molecule (see also text S12E).

These findings were included in the final model described further below, for which all NMR parameters are taken into account. Important differences are the Asn^230^ orientation and the location of the proposed water molecule between Asn^230^ and Trp^197^ in GPR, which shifts close to the pSB in BPR. In GPR, this water molecule is proposed to bridge Trp^197^ side chain and Asn^230^ backbone C′, while the hydrophobic Leu^105^ side chain is excluded from this interaction cluster (Fig. 3B). In BPR, this water molecule is suggested to switch its position away from Trp^197^ to promote the formation of a Gln^105^-Trp^197^-Asn^230^ side-chain interaction triad (Fig. 3C).

For model validation, we tried to detect chemical shift differences between GPR and BPR for Asn^230^, as these should reflect the proposed structural changes. Unfortunately, asparagine residues cannot be selectively labeled in *E. coli* without severe isotope scrambling. However, we were able to detect the ^15^N signal of side-chain Nδ2 of Asn230 via dipolar through-space correlation with nearby Tyr^200^ phenyl ring carbon in [U-^13^C Tyr, U-^15^N]-PR samples differences (text S11G). As chemical shifts of both Asn side-chain nitrogen and Tyr side-chain carbons are rather distinguishable from many other protein NMR signals, the Asn^230^ Nδ2–Tyr^200^ ring carbon cross peaks have been unambiguously identified in 2D ^15^N-^13^C DCP spectra (fig. S15). The observed Asn^230^ Nδ2 chemical shift difference between GPR and BPR (3.8 ppm; table S6) can be reproduced qualitatively by AF-QM/MM calculations based on the proposed models (Fig. 3, B and C). To further validate these models, we have introduced an Asn^230^Ser mutation in both GPR and BPR and have monitored its effect on Trp^197^. This mutation is expected to perturb the direct Asn^230^-Trp^197^ interaction in BPR rather than Asn^230^-water-Trp^197^ interaction in GPR. Upon Asn^230^Ser mutation, the Nε1 chemical shift of Trp^197^ in GPR_N230S_ is not much affected, but it drifts drastically in BPR_N230S_ to a value similar to that in GPR_N230S_ (Fig. 3D, right), which supports the proposed structural models. In these experiments, the Trp^197^ Nε1 signal was selected via dipole interactions to the adjected retinal carbon C20.

We have next obtained further experimental evidence for the proposed water molecule that switches its location and interaction partners between GPR and BPR (Fig. 3, B and C). 2D ^1^H-^13^C HETCOR spectra of [^13^C_10–18_-ret, U-^15^N]-PRs before and after hydrogen/deuterium (H/D) exchange (Fig. 3E and text S5) show that an exchangeable proton is present near retinal C11 in GPR but not in BPR. This observation agrees with the different water locations proposed by our models (Fig. 3, B and C). The suggested water locations in GPR and BPR are further supported by molecular dynamics (MD) simulations (Fig. 3F and text S14), which show stable water molecules at distinct positions near the color switching residue in both PRs.

**Fig. 4.**
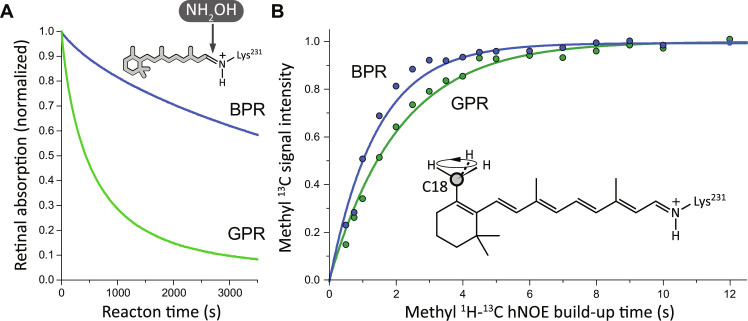
L105Q causes alterations of the retinal accessibility and ionone ring. (**A**) NH_2_OH chemical bleaching of the retinal chromophore probes the accessible of the binding pocket for small molecules. The GPR bleaching kinetics was substantially faster compared to BPR, indicating much better accessibility. (**B**) hNOE DNP build-up kinetics of the retinal C18 methyl group was used to probe the local molecular packing. The observed differences between GPR and BPR demonstrate that the color-determining L105Q mutation allosterically affects the ionone ring–protein interaction.

These different locations of the proposed water molecule might also be reflected in differences in general water accessibility of the region between Asn^230^ and pSB. The existence of a water-accessible region stretching toward Asn^230^ and pSB in GPR has been shown by a previous ssNMR mapping of exchangeable backbone amide protons (*15*). Here, we have probed the accessibility of this region for both PRs by hydroxylamine chemical bleaching experiments, which reports on small-molecule accessibility of the pSB site by cleaving the retinal cofactor from the opsin. The bleaching kinetics (text S20) exhibit evident differences (Fig. 4A) between GPR and BPR as inferred by our models. GPR is bleached much faster than BPR. In summary, our data point coherently to structural differences within the intracellular side of retinal binding pocket between GPR and BPR involving bound water.

### The impact of the color mutation on the retinal ionone ring

We have then wondered whether the drastic local structural reorganization around the color switching residue propagates further to other parts of retinal binding pocket and affects the retinal ionone ring. As shown in Fig. 3A and discussed above, the bulky side chain of Trp^159^ is located close to ionone ring moiety of retinal chromophore and shows chemical shift differences between GPR and BPR. Structural perturbation of Trp^159^ may therefore affect the ionone ring, which defines the end of the π-conjugation system and influences its absorption maximum.

To test this hypothesis, we have first probed whether the ionone ring and Trp^159^ interact. Therefore, an additional double bond between the C3 and C4 carbons on the retinal ionone ring [(3,4)-deH2-retinal] has been introduced. As a result, Trp^159^ Nε1 shifts by 0.5 ppm in GPR and 1.0 ppm in BPR (Fig. 3D, middle), indicating that the Trp^159^ side chain is indeed in structural communication with the retinal ionone ring. Smaller chemical shift changes also occur on Trp^197^ Nε1 (GPR: 0.4 ppm, BPR: 0.2 ppm), which suggests that the retinal ionone ring also communicates remotely with this residue located next to the color determining residue 105.

To further address the structural perturbation on the ionone ring, we have focused on its C18 methyl group, which faces the Trp^159^ side chain. It has been shown that the rotational dynamics of methyl groups drives the ^1^H-^13^C heteronuclear Overhauser effect (hetNOE), which, under DNP condition, leads to a negative enhancement of NMR signals of methyl carbons (*32*–*34*). Recently, we have shown that the build-up kinetics of such negative NMR enhancements reports on local molecular packing of methyl groups (*25*). Here, we have applied this method to probe the local molecular environments of retinal methyl groups C18 and C20 (text S10). As shown in Fig. 4B, the build-up kinetics of the C18 methyl carbon exhibits pronounced differences between GPR and BPR, which provides direct evidence for alterations in its interactions with the environment. In contrast to C18, the C20 methyl group, which is located close to Trp^197^ indole ring and Leu/Gln^105^, shows almost no differences in its hetNOE build-up kinetics (fig. S8). Therefore, it appears that the L105Q color-tuning mutation causes structural perturbations that propagate further to distal protein sites and remotely affect ionone ring-protein interactions.

### Chemical shift–driven structural modeling of PRs via AF-QM/MM

The NMR experiments described above have identified a number of spectroscopic differences between GPR and BPR within the retinal and in the retinal binding pocket. Our goal was to use these NMR parameters along with 3D structural models to provide an explanation for why the L105Q mutation converts GPR to BPR in such a highly specific manner.

The NMR data and the now available x-ray (*17*) and cryo-EM (*18*) structures of PRs alone are not sufficient to fully explain the observed color shift. In principle, our chemical shift parameters are very informative because they respond to small changes in molecular and electronic structures. But these NMR data are highly selective, and a structural interpretation has remained qualitative. The available 3D structures of PRs are not of sufficient resolution and have not been obtained under color-tuning relevant conditions. Our initial density functional theory (DFT) calculation (see the next section for more details) on the GPR cryo-EM structure [PDB 7b03 (*18*)] and on the BPR x-ray structure [PDB 4JQ6 (*17*)] predicts absorption maxima of 640 nm and 622 and 627 nm, respectively, which deviate strongly from the experimental values. We therefore derived an approach in which 3D structural models of GPR and BPR are refined on the quantum chemical level based on our chemical shift data. The resulting models are eventually used for calculating the absorption maximum. In this way, the main differences responsible for the color can be deciphered and identified, since the absorption maximum is very sensitive to multiple, finely coupled structural and electronic changes in the retinal chromophore and its protein environment.

Here, we used the BPR x-ray structure (PDB 4JQ6) as the initial 3D model and created a GPR homology model. In this homology model, in accordance with the design of our NMR experiments, the L105Q mutation was then introduced. To be in accordance with the experiments, simulation conditions were chosen that correspond to a pH > p*K*_a_ (D97), where *K*_a_ is the acid dissociation constant, by adding hydrogen atoms using the Leap module in the AMBER program. The amine groups were fully protonated (Lys and Arg residues and N terminus), and the carboxylic groups were deprotonated (Asp and Glu residues and C terminus). As a result, D97 and D227 were deprotonated. All His residues were left neutral and protonated at the ND1 position based on the local electrostatic environment.

These initial models have been then further improved via QM/MM supported by NMR data. Refining these models by chemical shift and CSA data at the quantum chemical level represents a major computational challenge. Although some earlier works have shown the feasibility of such an approach on smaller systems (*35*–*37*), the central issue remains a problem, namely, the highly demanding QM treatment of large molecules such as PRs. For resolving this bottleneck, we have chosen AF-QM/MM for calculating our NMR parameters, which offers high QM precision at low computational cost thanks to its linear scalability. On the basis of AF-QM/MM, we were able to explore a retinal binding pocket region with a total of 1313 atoms (approximately one-third of the total protein), which is one to two orders of magnitude larger than the regions explored in previous NMR-based protein structure modeling work (*35*–*37*). The method also permits the computation of chemical shifts of protein side chains and ligands, which are essential for our structural remodeling but could not be accessed by other methods (*19*, *38*, *39*).

Briefly, our approach explores retinal and protein conformations toward better matching between experimental and QM-predicted chemical shifts. First, we have remodeled the protein part to reproduce the observed chemical shift changes between GPR and BPR. We have kept the global fold of PR unchanged, which is strongly supported by our previous NMR study. This has permitted us to focus on residues in retinal binding pocket and has further reduced the computational complexity. A prototypical example is already shown in the above section (Fig. 3). Second, we have jointly optimized the structures of retinal chromophore and its surrounding residues. In particular, ^13^C CSA has permitted us to explore the fine structure of retinal moiety, as these parameters are sensitive to even small structural changes of this molecule. For example, a bond length changes down to 0.01 Å on retinal π-conjugation system can be sensed by retinal carbon CSA (*35*). Last, we have explored the additional experimental results described above (water location and ionone ring interactions), which have been left out in the first two rounds of structural remodeling, as additional validations of our remodeled PR structures. It should be emphasized that our approach requires multiple rounds of structural adjustment, which therefore benefits extensively from the reduction of computational burden by the AF-QM/MM method. The pipeline of our approach is visualized in fig. S17. The detailed descriptions can be found in text S12. More methodological advancements of choosing AF-QM/MM as the computation engine are discussed in details in text S12 (A and B) along with examples shown in fig. S16 and table S5.

**Fig. 5.**
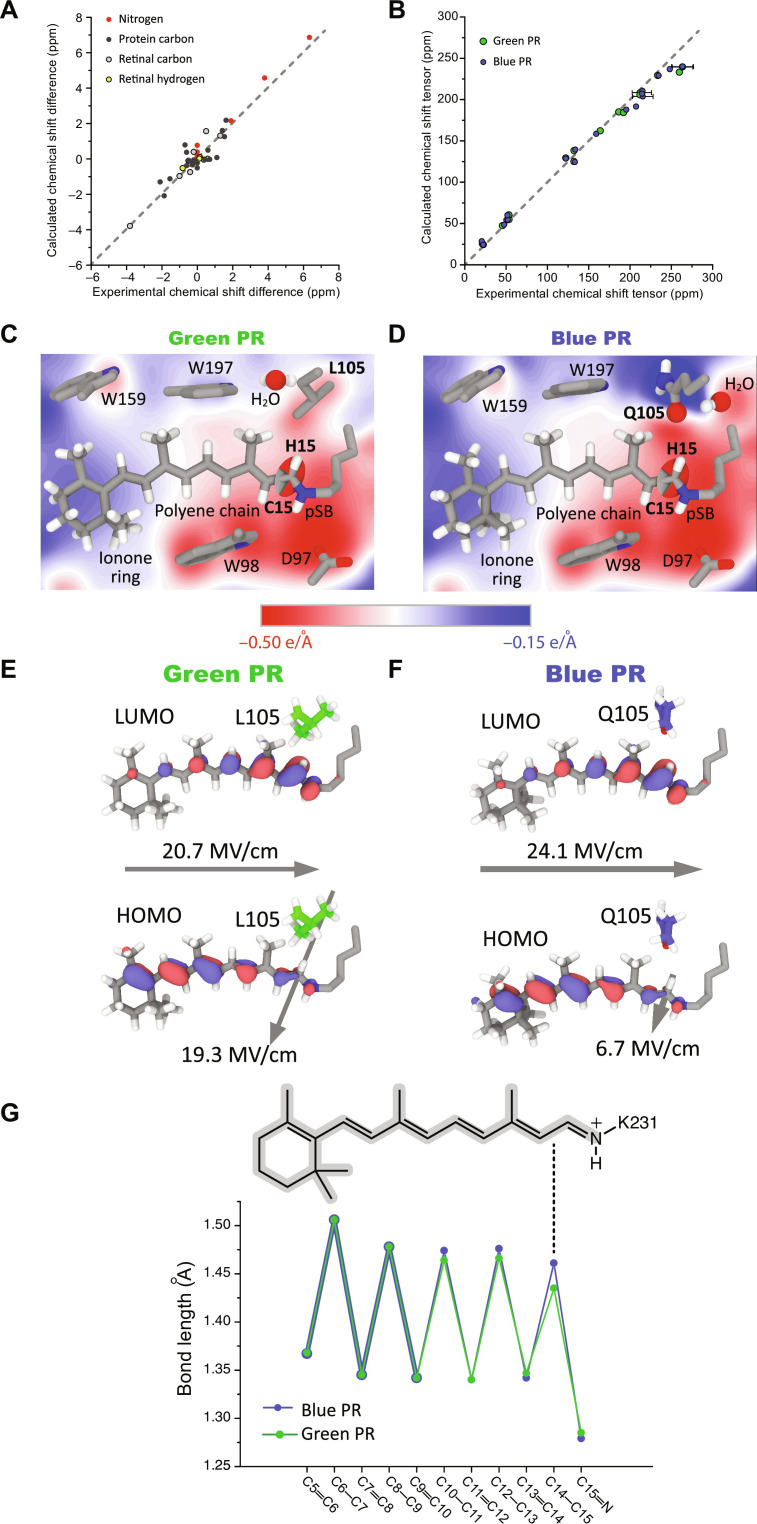
The molecular details of color switching in PRs. (**A** and **B**) AF-QM/MM–refined structural models of GPR and BPR reproduce the experimental chemical shift parameters. Isotropic chemical shifts of retinal and protein are shown in (A). Retinal chemical shift tensor elements are plotted in (B). (**C** and **D**) Calculated electrostatic potential within the refined GPR and BPR binding pocket. The color mutation created different electrostatic potential gradients within GPR and BPR resulting in E-fields not only along the C15─H15 bond but also along the polyene chain. The carbon CSA tensor of retinal C15 is presented as a 3D ellipse. (**E** and **F**) HOMO and LUMO representation of the retinal chromophore in GPR and BPR. The local E-fields are highlighted by arrows. (**G**) Calculated bond lengths of the retinal polyene chain in GPR and BPR (table S8). Color mutation–induced differences mainly affect the bond length at the end of the polyene chain.

As shown in fig. S18, after our structural remodeling, the agreement of experimental and QM-predicted NMR parameters of both retinal binding pocket and retinal chromophore has been clearly improved (tables S9 and S10). In particular, the chemical shift differences in (table S6) and carbon CSA values of retinal (table S7) can be reproduced rather well from our structural models (Fig. 5, A and B). We have also compared the structures of retinal binding pocket in our models with those in various structural models obtained via x-ray crystallography (*17*), cryo-EM (*18*), and AlphaFold2 (*40*). As shown in table S11, our remodeled PR structures differ from those determined by other methods at similar level, indicating that fine structural differences have been captured. On the basis of these refined models, the electrostatic potential within the retinal binding pocket (Fig. 5, C and D) and structural perturbations along the retinal polyene chain (Fig. 5G) can be calculated.

### Molecular mechanism of color switching in PR

**Fig. 6.**
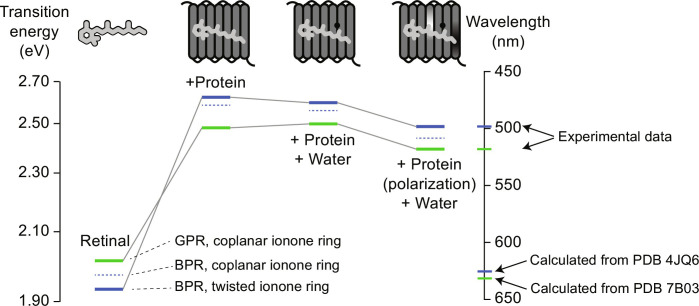
TD-DFT calculations predict the color differences from the refined GPR and BPR structural models. Calculations were performed on retinal without and with protein environment, with bound water, including full electrostatics and by taking ionone ring reorientations into account. The AMBER charge model (nonpolarized fixed charge model) was used for the nonpolarized and the polarized protein-specific charge model for the polarized protein environment (see text S16 and tables S12 and S13 for further details.

Time-dependent density functional theory (TD-DFT) calculations been shown in the past to predict successfully colors of retinal proteins (*41*–*43*). As mentioned in the previous section, TD-DFT calculations suggest that the GPR cryo-EM (7BO3) and the BPR x-ray structures (4JQ6) (*17*, *18*) could not be used directly for explaining the color switching (table S13), which is due to limitations of the structural resolution and the nonphysiological sample conditions (pH value). The situation improves strongly by using our GPR and BPR structural models, which have been optimized in the retinal binding pocket using NMR data recorded under more color-tuning relevant sample conditions. In this way, the L105Q-induced color difference could be reproduced correctly on our NMR-guided structural models (Fig. 6 and table S13). On the basis of our data and TD-DFT calculations, we decipher below step by step how the color-changing residue Leu/Gln^105^ causes such a remarkable color shift.

On the basis of our NMR-based structure models, the L105Q color-determining mutation together with its adjacent residues and bound water induces highly localized changes of the electrostatic potential at the end of the retinal polyene chain. These changes coincide with experimentally observed structural perturbations in the form of chemical shift and CSA changes and bond length alterations as described above. Sole steric effects caused by Leu/Gln^105^ as a source seem unlikely, as the rotation of the nearby C20 methyl group remains undisturbed. The electrostatic potential differences within GPR and BPR create a local electrostatic field (E-field), which is approximately oriented along the C15─H15 bond (Fig. 5, C and D). In GPR, its magnitude is predicted to be stronger than in BPR, which explains larger local structural perturbations on the retinal chromophore. The E-field polarizes the C15─H15 bond and causes opposite changes of the H15 chemical shift and the δ11 component of the C15 CSA tensor (Fig. 2E). This electrostatic tuning of the C15─H15 bond further propagates along the retinal polyene chain through the π-conjugation system but with decaying structural effects as shown by the bond length alternation pattern (Fig. 5G).

Electrostatic control of ligand chemistry has been shown in a number of proteins particularly enzymes and fluorescent proteins (*44*–*46*). In GPR/BPR, the local E-field on the retinal chromophore is in the low-10^1^ MV/cm regime, which is 3- to 10-fold weaker than in previously studied proteins (*44*, *45*, *47*, *48*). Such a low field still permits a fine structural tuning of the retinal chromophore while preventing strong interference with retinal photochemistry (trans-to-cis photoisomerization of the C13═C14 bond).

TD-DFT calculations of the resulting GPR/BPR retinal structure alone in vacuum lead to a color shift of opposite trend (615/627 nm) compared to that found experimentally (520/500 nm), but this effect is already partially compensated by taking the protein environment without bound water into account (498/478 nm) (Fig. 6 and table S13). Our simulations show that the local E-field along the C15─H15 bond is also partially regulated by the water molecule close to the color switch (Fig. 5, C and D). The role of conserved water molecules in defining functionally important E-fields in proteins has been reported before, for example, for kinases.(*47*). TD-DFT calculations taking the predicted water locations in GPR and BPR into account cause further shifts of both absorption wavelengths (495/483 nm) (Fig. 6 and table S13). Notably, our MD simulations show that the stability of the water molecule near the color switch relies on the charge distribution of its protein environment. In particular, the constant water occupation at this location in GPR only occurs with a polarized force field. The lifetime of the water molecule becomes rather short (ca. 6.4 ps) in MD simulations using the conventional CHARMM force field with a nonpolarized point charge model. Our previous works have also demonstrated that the electronic polarization is of critical importance for stabilizing hydrogen bonds between the protein and surrounding water molecules (*49*, *50*).

Our structural models further show that the electrostatic potential differences within GPR and BPR (Fig. 5, C and D) also create an E-field, which is pointing along the retinal polyene chain. The difference in the field magnitude is mostly defined by the difference in the electrostatic potential around the C15 carbon position between GPR and BPR as discussed above. This E-field stabilizes the charge-separated retinal ground state [highest occupied molecular orbital (HOMO)] more than the less polar light-excited state [lowest unoccupied molecular orbital (LUMO); Fig. 5, E and F, and fig. S19 and S20]. The larger E-field in BPR therefore increases the HOMO-LUMO energy gap and contributes to the blue-shift of the light absorption wavelength in this protein. Subsequent TD-DFT calculations including the retinal conformation, a polarized protein environment, bound water, and the E-field yield a further shift of both GPR/BPR absorption maxima (517/507 nm) (Fig. 6 and table S13).

Although the color switching trend can be partially reproduced when including all of these factors, the absolute values of the absorption maxima and their difference still deviate from the experiment. In these simulations, coplanarity of the ionone ring and the polyene chain was assumed. Our experimental data (Fig. 3) however indicated a long-range response toward the L105Q switch in BPR via a Trp^197^-Trp^159^-retinal C18 methyl pathway. The TD-DFT calculations were therefore repeated for each of the BPR cases described above after introducing a twist of the ionone ring around the C1-C9 vector. For BPR, a deviation from coplanarity of 35° is obtained, while GPR remains almost planar (2°). This additional ring orientation causes a redshift for retinal in vacuum but a blue-shift in all other cases (Fig. 6 and table S13). With this additional parameter, the 20-nm maximum light absorption wavelength difference and also correct absolute absorption maxima can be computationally reproduced. Without this contribution, the BPR absorption wavelength would be offset by about 10 nm, making it non-optimal for the fitness in deep photic zone.

In summary, our analysis shows that PR color switching is controlled by multiple structural, chemical, and physical factors, which are all triggered initially by the same mutation but involve different parts of the protein and chromophore. (Figs. 5, C to G, and 6). These factors lead to distinct contributions to the color tuning, featuring opposite trends in color regulation that are balanced lastly to reach the optimal light absorption wavelengths. In particular, local E-fields within the GPR and BPR retinal binding pocket play a twofold role in color tuning: one local E-field modifies the retinal structure by targeting the retinal C15─H15 bond, while another E-field along the polyene chain couples with the HOMO and LUMO of retinal chromophore. Size and orientation of both electrostatic fields are affected by the color switching residues and adjacent residues in retinal binding pocket. They contribute in an opposite trend to the observed color tuning. Besides of affecting the electrostatic fields within the retinal binding pocket, the color switch also remotely regulates the retinal ionone ring, which further contributes to color tuning. Our analysis, on the basis of the NMR-guided structural models of GPR and BPR, reveals an unexpected mechanistic complexity of the observed color difference, which can now be correctly reproduced by computation.

Our findings fill a number of knowledge gaps left by the previous efforts to explore the molecular mechanism of PR color tuning. As outlined above, the known structures (*17*, *18*) alone are not sufficient to explain the color switch. A previous mutational analysis (*11*) led to the proposal that specific local interactions involving the Schiff base, triggered mainly by volume differences of the side chains at position 105, are related to the absorption wavelength. However, it remains difficult to address the cause of PR color tuning based on these biochemical data alone. Our own previous ssNMR study (*15*) identified some changes within the retinal binding pocket. However, these data were restricted to the C14-C15 segment of the retinal chromophore and limited number of sites of the retinal binding pocket, therefore insufficient to explain the color shift (*13*, *51*). On the other hand, hybrid QM/MM simulations in combination with MD simulation (*17*) have suggested that the color shift is related to changes in the electrostatic potential around the C14-C15 (*13*). Further improvements in the prediction of the absorption maximum have been achieved by assuming bound water molecules at positions known from other proton pumps (*51*). These theoretical studies partly lack strong experimental support and therefore cannot fully describe the causal mechanism leading to the observed color change. In this study, our extensive experimental data combined with advanced computational methods reveal a much more complex interplay of structural changes throughout the retinal binding pocket and the protein E field. Our integrative approach allows the identification of the main factors contributing to the color change effect and their sources. The L105Q mutation triggers a series of conformational changes within the retinal binding pocket as well as the retinal chromophore including the ionone ring. Our data also show a rearrangement of bound water within the binding pocket.

### Robustness of the PR color switch

**Fig. 7.**
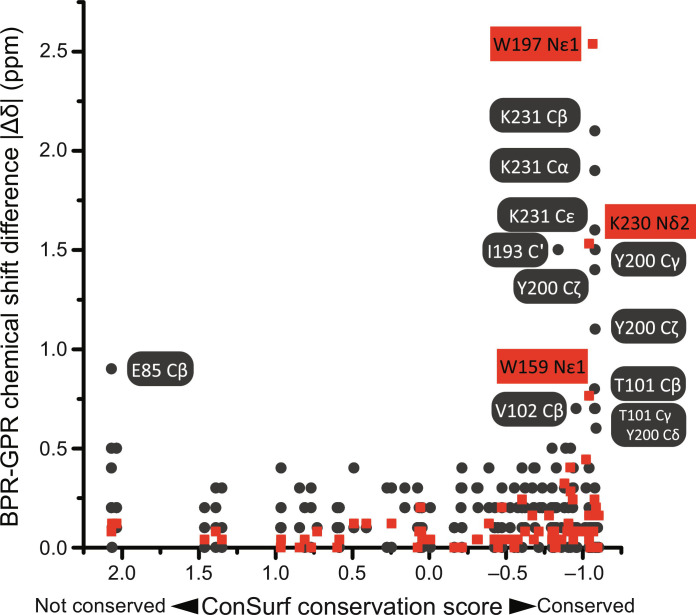
Correlation of the observed BPR-GPR chemical shift differences with the degree of residue conservation. The conservation is quantified using the ConSurf score (*26*, *27*) for the PR family (see also fig. S22). Larger chemical shift differences are observed for residues with a higher degree of conservation. Black dots represent ^13^C chemical shift changes, and red squares show ^15^N chemical shift changes scaled by gyromagnetic ratios. Correlation of the observed BPR-GPR chemical shift differences with the degree of conservation of the affected residues within the PR family (GPRs and BPRs). The degree of conservation on the *x* axis corresponds to Fig. 1D. An alternative representation is shown in fig. S24 in which the chemical shift differences are plotted against the degree of conservation on the *x* axis corresponding to Fig. 1D.

Our comprehensive characterization of the two PR color variants also provides evidence for the robustness of PR color switching to sequence diversification. We found that Asn^230^, a key factor in color change as shown by chemical shift perturbation, is highly conserved in the PR family. The Asn^230^Ser mutation selectively shifts the light absorption wavelength of BPR toward the GPR phenotype (from 500 to 512 nm; fig. S21, A and B). We have then decided to map experimentally observed chemical shift perturbations versus the degrees of residue conservation in evolution across the PR family (Fig. 7 and fig. S24). Similar to Asn^230^, most residues that respond to the color switch are highly conserved (Fig. 7). One exception is Ile^193^, which participates in color switching via its residue type–independent backbone C′═O group. It seems that the PR color switch exploits selectively certain highly conserved partner residues in the retinal binding pocket for maintaining robustly the color phenotype difference against protein sequence diversity. In addition, the involvement of highly conserved retinal binding pocket residues also maintains the stability of color phenotype within both PR subgroups. It has been shown that protein fitness forms a bottleneck for adaptive evolution (*52*–*54*). The participation of a number of highly conserved residues in PR color tuning reduces the number of mutations needed for this phenotype shift, which makes it more tolerable in adaptive evolution.

Protein fitness comes from both protein stability and functionality. The structural perturbations required for PR color switching are well tolerated by the protein architecture. For example, the highly conserved H-bond between Trp^159^ (helix 5) indole Nε1-Hε1 and Ile^193^ backbone C′═O supports both protein structural stability (interhelical arrangement) and color switching. The minor structural perturbations on this site required for color switching only lead to 1.6 kJ/mol change in local protein stability as estimated by our calculations. Our ssNMR studies also show that the structural perturbations triggered by the color switch are mostly limited to the intracellular side of retinal binding pocket, leaving the extracellular side of this pocket unaffected. The functional residues for initial proton transfer steps, including the highly conserved pSB counterions Asp^97^ and Asp^227^, are all located at the extracellular side of the retinal binding pocket. The restrained structural perturbations could protect the proton transport functions from color switching as shown by previous time-resolved flash photolysis data (*15*), therefore maintaining the protein functionality of these proton pumps during evolution. Here, evolution seems to reach a balance between protein evolvability and fitness via a network of highly conserved residues as phenotype controllers. Although most microbial rhodopsins have a very similar global 7 transmembrane helices (7TM) fold, they have different local structures and internal interaction networks that represent structural barriers to the functional transfer of this PR color phenotype to some other microbial rhodopsins (fig. S21).

In conclusion, we have addressed the molecular mechanism of PR color switching and its robustness against sequence diversity in evolution. The integrative biophysical approach developed here, bringing together advanced ssNMR spectroscopy, tailored isotope labeling, biochemistry, AF-QM/MM calculations, MD simulations, TD-DFT, and bioinformatics, has also unraveled several structural, biophysical, and chemical factors contributing to PR color change. In particular, our work has revealed the interplay of protein structure, electrostatic field, water molecules, chromophore conformation, and ligand chemistry in the regulation of protein function. A group of highly conserved interacting residues controls the PR color phenotype via a single residue under highly diverse protein sequence backgrounds. Our findings reconcile protein functional precision, tunability, and robustness in evolution. Now, residue coevolution, epistasis, and other mutagenic features are intensively exploited for sampling the structure, dynamic, energy, fitness, and functional landscapes, which often feature additive and/or cooperative mutations (*52*–*55*). The molecular mechanism of PR phenotype switching unlocked by our studies infers an alternative, economical, and robust approach by taking the advantage of highly conserved sites imprinted in protein architecture. The biophysical methods developed in this work can be transferred feasibly to a large range of other ligand-binding proteins such as optogenetic proteins, enzymes, and drug receptors. Our findings will help rational protein engineering of this protein family in the future.

## MATERIALS AND METHODS

### Preparation of GPR and BPR samples

All sample preparation steps (expression in isotope-labeling media, incorporation of labeled retinal, purification, and reconstitution into liposomes) were carried out as described by us previously (*15*, *56*, *57*). A detailed protocol is provided in text S2. All samples are summarized in table S1. For completeness and reproducibility, a brief summary is given here.

The GPR construct (UniProt Q9F7P4) from γ-proteobacterium strain EBAC31A08 (SAR86 clade) was subcloned into pET27b(+) plasmid between Nde I and Xho I sites. The C-terminal herpes simplex virus tag and its consecutive hexahistidine tag were kept in frame for protein purification. The blue-shift mutation L105Q was introduced into GPR to which we refer as BPR. In addition, I193V and N230S mutants of GPR and BPR were prepared. The corresponding constructs were purchased from GenScript. The proteins were expressed in *E. coli* C43 strain in M9 minimal medium.

For amino acid–type selective labeling or reverse labeling, specific amino acids were added to the medium. For uniform ^15^N labeling, ^15^NH_4_Cl was used as nitrogen source. The expression of PRs was induced by adding isopropyl-β-d-thiogalactopyranoside. The retinal stock (10 mg/ml in ethanol, 200 μl/liter culture) was also added at the point of induction. After harvesting, cells were disrupted by passing them through a continues flow cell disruptor (Constant Systems). For protein purification, the membrane fraction was solubilized in 2% (w/v) n-Dodecyl-Beta-Maltoside (DDM) [50 mM MES, 300 mM NaCl, and 5 mM imidazole (pH 6.5)] at 4°C overnight. The insoluble materials were removed by ultracentrifugation (42,000 rpm) at 4°C for 1 hour. The supernatant was incubated with nickel nitrilotriacetic acid (Ni-NTA) beads (Qiagen) under gentle stirring at 4°C for 1 hour. The beads loaded with proteins were then collected in a gravity column washed with precooled buffer [50 mM MES, 300 mM NaCl, 50 mM imidazole, and 0.15% DDM (pH 6.5)] and eluted with 50 mM MES, 300 mM NaCl, 500 mM imidazole, and 0.05% DDM at pH 7.5. For reconstitution, the eluted protein was added into a 1,2-dimyristoyl-sn-glycero-3-phosphocholine/1,2-dimyristoyl-sn-glycero-3-phosphate stock solution (4 mg/ml) drop by drop to reach the final lipid-to-protein ratio (w/w) 1:2. The mixture was incubated at room temperature for 30 min. Biobeads (Bio-Rad) were then added (80 mg beads/ml solution), and the mixture was incubated at room temperature for 2 hours with gentle rotation. Biobeads were then removed via a cell drainer, and the turbid proteoliposome suspension was subjected to ultracentrifugation (55,000 rpm, 1 hour). The pellet was resuspended in 1.5 ml of 50 mM tris and 5 mg of MgCl_2_ (pH 9.0) and collected again by ultracentrifugation (55,000 rpm, 1 hour). The pellet was then collected and centrifuged into a 3.2-mm MAS NMR rotor. For DNP-enhanced ssNMR experiments, the proteoliposome pellet was incubated with AMUPol solution [20 mM AMUPol, 7/3 (v/v) NMR buffer/glycerol mixture, and 200 μl for about 30 μl of pellet] at 4°C for 12 to 16 hours. No deuterated components were used for the DNP matrix in our sample preparations (*25*), which prevents the partial deuteration of the pSB site. For VFMAS ssNMR experiments (S3), GdDOTA (Macrocyclics, 100 mM stock solution in water) was added to reach the final concentration of 0.1 mM for reducing the ^1^H T1. The pellet was transferred into a 1.3-mm rotor using a customized ultracentrifuge packing device (*58*).

Microbial rhodopsins *Exiguobacterium sibiricum* rhodopsin, *Nonlabens marinus* rhodopsin 2, *Nonlabens marinus* rhodopsin 3, and *Natronomonas pharaonis* rhodopsin have been cloned to pET plasmids and expressed recombinantly in *E. coli* C43 strain. Genes were synthesized by GenScript (www.genscript.com). Expression and purification followed the protocol used for GPR and BPR. Further details are provided in text S18 and tables S14 and S15.

### Isotope-labeled retinals and retinal analogs

The ^13^C-labeled all-trans retinals (^13^C_10–18_-retinal and ^13^C_12,13,20_-retinal) were synthesized as reported previously (*25*, *59*). The 3,4-deH_2_ retinal was obtained from BioSynth and used without further purification. Retinals were incorporated into proteoopsin in *E. coli* membrane suspensions prior purification using a previously established protocol (*57*).

### UV-Vis spectroscopy

All microbial rhodopsins (wild types and mutants) used in thus study have been characterized by ultraviolet-visible (UV-Vis) spectroscopy using a Jasco V550 instrument. All spectra were recorded at a pH above the p*K*_a_ of the Schiff base counterion. Further details are provided in text S18 and S19.

### Retinal bleaching experiments

The covalently bonded retinal chromophore in PRs has been cleaved off by addition of NH_2_OH and followed by real-time UV-Vis spectroscopy. An imidazole-free buffer [300 mM NaCl, 50 mM tris, and 0.05% DDM (pH 8)] was used. The protein concentration was adjusted to about 17.5 μM. The NH_2_OH concentration was adjusted to 30 mM. The absorption at 500/520 nm was recorded every 5 s with an initial dead time of about 20 s. Further details are provided in text S20.

### NMR spectroscopy

^13^C-^1^H HETCOR and ^13^C-^13^C PDSD spectra were recorded on a Bruker Avance III wide-bore ssNMR spectrometer operating at 850 MHz (^1^H Larmor frequency) using a Bruker 3.2-mm MAS HCN probehead operating at a MAS rate of 14 kHz and a temperature of 290 K. Further details are given in text S3 and S4.

Direct proton detection based on VFMAS NMR was achieved on a Bruker Avance III narrow-bore 800-MHz (^1^H Larmor frequency) spectrometer with a 1.3-mm VFMAS HCN probehead using a 60-kHz MAS rate and a sample temperature of 300 K. Further details are given in text S4.

All NMR experiments using DNP were performed using a Bruker Avance II spectrometer operating at 400.197 MHz (^1^H Larmor frequency, 9.4 T) connected with a gyrotron operating at 263.580 GHz (microwave frequency) and a 3.2-mm eHCN DNP MAS probehead. All DNP ssNMR experiments were carried out using 8-kHz MAS sample spinning frequency and at about 110 K. All experimental details are given in text S6 to S10. Experimental parameters are summarized in table S4.

### AF-QM/MM chemical shift calculations

The isotropic chemical shifts and/or chemical shift anisotropies of various nuclei (^1^H, ^13^C, and ^15^N) were calculated by AF-QM/MM approach proposed by He and co-workers (*39*, *50*, *60*, *61*). In the AF-QM/MM approach, each individual amino acid is taken as the core region. The buffer region for the *n*th core region is defined by the following criteria: (i) sequentially connected (*n* − 2)th, (*n* − 1)th, (*n* + 1)th, and (*n* + 2)th residues (neighboring residues); (ii) nonneighboring residues outside the core region with an atom less than 4 Å away from any atom in the core region; (iii) nonneighboring residues outside the core region with a hydrogen atom less than 3 Å away from a hydrogen atom in the core region; (iv) nonneighboring residues with a heavy atom on its aromatic ring less than 5 Å away from any atom in the core region. Both the core and the buffer regions are treated by quantum mechanics, while the rest of the protein is described using the point charge model to account for the electrostatic effect (electrostatic embedding). Each core-centric (core and buffer) QM/MM calculation is carried out separately, and only the shielding constants of the atoms in the core region are extracted from individual QM/MM calculations. Further details are provided in text S12 and S13 including figs. S16 and S17 and table S5.

### MD simulations

The MD simulations were performed using AMBER 16 with graphics processing unit (GPU) acceleration (*62*). The simulation was performed at a constant temperature of 300 K in the isothermal–isobaric (NPT) ensemble, using Langevin dynamics with a friction coefficient of 1 ps^−1^. The constant pressure is 1 bar and is controlled by Berendsen pressure scaling algorithm with a relaxation time of 8 ps. The SHAKE algorithm was used to constrain the hydrogen atoms. The particle-mesh Ewald method was applied for electrostatics calculations with nonbonded cutoff of 8.0 Å. Each simulation was integrated at a time step of 2 fs for a total MD trajectory of 10 ns. The MD simulations were performed on an in-house GPU cluster (GeForce GTX 2080 Ti). Further details are provided in text S14.

### TD-DFT calculations

The excited state calculations for PRs were performed using the QM/MM method at the TD-B3LYP/6-311 + G* level (*41*–*43*). The QM region contains the whole retinal, the pSB, and the linked Cε atom (on Lys^231^). The dangling bond was capped by a hydrogen atom (*41*). The other residues along with the bridging water in the pocket were treated as background charges. We applied polarized protein-specific charge model for the electrostatic embedding field in our calculations. The molecular orbital files were exported from GaussView. Further details are provided in text S15 to S17.

### Bioinformatic analysis

PR sequences have been obtained from four sources (*7*, *23*, *63*, *64*). After alignment, they have been classified into GPR (686 sequences) and BPR (2154 sequences) according to the location of Leu or Gln at position 105. The degree of conservation at each residue site across both PR groups has then been calculated by in-house Python scripts followed by a more in-depth analysis using ConSurf (*26*, *27*, *65*) and MISTIC2 (*28*) to take the uneven sequence similarity and phylogenetic structure into account. Further details are provided text S21.

## Supplementary Materials

### This PDF file includes:

Text S1 to S21 Figs. S1 to S24 Tables S1 to S15 References
