# Supplementary material for: Molecular mechanisms and evolutionary robustness of a color switch in proteorhodopsins
Source: Sci Adv. 2024 Jan 24;10(4):eadj0384. doi: 10.1126/sciadv.adj0384 (PMC10807816; doi:10.1126/sciadv.adj0384)
Supplement: Supplementary file 1 — Text S1 to S21 Figs. S1 to S24 Tables S1 to S15 References [file sciadv.adj0384_sm.pdf]

Science Advances
AAAS

Supplementary Materials for

Molecular mechanisms and evolutionary robustness of a color switch in proteorhodopsins

Jiafei Mao *et al.*

Corresponding author: Jiafei Mao, j.mao@em.uni-frankfurt.de; Xiao He, xiaohe@phy.ecnu.edu.cn; Clemens Glaubitz, glaubitz@em.uni-frankfurt.de

Sci. Adv. **10**, eadj0384 (2024)
DOI: 10.1126/sciadv.adj0384

This PDF file includes:

Text S1 to S21
Figs. S1 to S24
Tables S1 to S15
References

Table of Contents

Overview and experimental details
Text S1: Brief description of approach and experimental pipeline
Text S2: Expression, purification and reconstitution of PRs
High-field room-temperature ssNMR experiments of retinal chromophore in PRs
Text S3: Retinal ^{13}C chemical shifts
Text S4: Retinal ^{1}H chemical shifts
Text S5: Retinal-water contact
DNP-enhanced ssNMR experiments of retinal chromophore and retinal binding pocket
Text S6: General setup of DNP-enhanced ssNMR experiments
Text S7: ^{15}N CSA of pSB nitrogen in PRs
Text S8: ^{1}H chemical shift of pSB hydrogen in PRs
Text S9: Retinal ^{13}C CSA and control experiments in native membranes
- (a) ^{13}C chemical shifts of retinal in PRs under DNP condition
- (b) ^{13}C chemical shifts of retinal in PRs in native cellular membranes
- (c) ^{13}C CSA of retinal polyene carbons in PRs

Text S10: hetNOE DNP build-up kinetics of retinal C18 and C20 methyl groups
Text S11: DNP-enhanced ssNMR mapping of retinal binding pocket residues
- (a) pSB nitrogen – counter ion (D97 and D227 Cγ) interactions
- (b) Schiff base residue Lys231
- (c) Tyr200 and Pro201
- (d) Trp98 sidechain
- (e) Ile193 – Trp159 and Ile192/retinal – Trp197 contacts
- (f) Val102 – Pro103 pair
- (g) Asn230 sidechain

AF-QM/MM-based structural modeling and PR color-tuning mechanism
Text S12: AF-QM/MM-based structural modeling of GPR and BPR
- (a) Background and challenges of chemical shift-based structural modeling
- (b) Rationale for AF-QM/MM calculations on PR chemical shiftss
- (c) General protocol of AF-QM/MM calculation
- (d) Derivation of the polarized protein-specific charges
- (e) Protocol for AF-QM/MM-based structural modeling of PR

Text S13: Analysis of PR structural modeling results
Text S14: MD simulations of PRs
Text S15: Derivation of protein electrostatic potential and electric field strength
Text S16: PR light absorption wavelength by TD-DFT calculations
Text S17: Analysis of contributors to PR color switching
UV-Vis spectra of microbial rhodopsins, mutants and retinal bleaching experiments
Text S18: Expression and purification of diverse microbial rhodopsins
Text S19: UV-Vis spectroscopic characterization of diverse microbial rhodopsins
Text S20: Retinal bleaching experiments
Bioinformatics
Text S21: Bioinformatic analysis of PR color switching

List of Figures

Fig. S1: Schematic overview of the experimental approach
Fig. S2: ^{13}C-^{13}C PDSD spectra of [$^{13}C_{10-18}$-ret, U-^{15}N]-GPR and [$^{13}C_{10-18}$-ret, U-^{15}N]-BPR
Fig. S3: ^{1}H-^{13}C HETCOR spectra of [$^{13}C_{10-18}$-ret, U-^{15}N]-GPR and [$^{13}C_{10-18}$-ret, U-^{15}N]-BPR
Fig. S4: DNP-enhanced ^{13}C and ^{15}N RNCSA recoupling experiments on retinal and pSB
Fig. S5: Fitting of recoupled ^{13}C and ^{15}N CSA patterns from retinal and pSB
Fig. S6: DNP-enhanced ^{1}H-^{15}N 2D HETCOR spectra of [U-^{13}C, $^{15}N\varepsilon$-Lys]-GPR and BPR
Fig. S7: DNP-enhanced ^{13}C-^{13}C 2D DQ-SQ spectra of [$^{13}C_{10-18}$-ret, U-^{15}N]-G/BPR
Fig. S8: ^{1}H-^{13}C hNOE build-up kinetics of retinal C20 methyl group at 110 K
Fig. S9: The counter ion chemical shifts in G/BPR
Fig. S10: The K231 ^{13}C chemical shifts in G/BPR
Fig. S11: The Y200 ^{13}C chemical shifts in G/BPR
Fig. S12: Detection of the indole nitrogen of Trp98 in [$^{13}C_{10-18}$-ret, U-^{15}N]-G/BPR
Fig. S13: Assignment of Ile192-Trp197 and Ile193-Trp149 contacts
Fig. S14: Val102-Pro103 correlations in G/BPR
Fig. S15: $^{15}N\delta_2$-Asn230 in G/BPR
Fig. S16: Demonstration of the AF-QM/MM approach
Fig. S17: Working flowchart of the AF-QM/MM structural modeling of PRs
Fig. S18: Analysis of calculated chemical shift parameters
Fig. S19: Electrostatic potential in the retinal binding pocket
Fig. S20: HOMO and LUMO of the retinal chromophore in G/BPR
Fig. S21: UV-Vis absorption spectra of microbial rhodopsins and their mutants
Fig. S22: Analysis of the conservations of each GPR and BPR residue site using ConSurf
Fig. S23: Inter-residue coevolution pattern of PRs obtained by MISTIC2
Fig. S24: Correlation of the BPR-GPR chemical shift differences with conservation

List of Tables

Tab. S1: Summary of NMR samples and ssNMR experiments
Tab. S2: Summary of GPR and BPR retinal ^{1}H, ^{13}C and pSB ^{15}N chemical shift data
Tab. S3: Comparison of GPR and BPR ^{1}H retinal chemical shift data
Tab. S4: Summary of acquisition and processing parameters for DNP ssNMR experiments
Tab. S5: Showcase of the sizes of core/QM buffer/MM regions in GPR for selected core regions
Tab. S6: Comparison of experimental and DFT simulated chemical shift differences
Tab. S7: Comparison of experimental and DFT simulated retinal chemical shift values
Tab. S8: Bond lengths of refined retinal polyene chain
Tab. S9: Summary of AF-QM/MM modeling results
Tab. S10: Analysis of chemical shifts without structural modeling
Tab. S11: Structural deviation of retinal binding pocket in various PR structures
Tab. S12: Electrostatic potential at retinal chromophore in PRs
Tab. S13: *In-silico* analysis of PR color switching mechanism
Tab. S14: Overview of additional microbial rhodopsins used here
Tab. S15: Overview of expression and purification conditions

Overview and experimental details

Text S1: Brief description of integrative approach and experimental pipeline

In our earlier work on the color tuning in PR (*15*), we have observed a large de-shielding of the retinal carbon C15, a longer C14-C15 bond and a slightly larger H-C14-C15-H torsional angle in BPR compared to GPR. In addition, chemical shift changes for a number of residues were identified. These data suggested that the color switch L105Q triggers only highly localized structural changes within PR and pointed to the end of the retinal molecule as the hotspot for color tuning. However, the side chains of functionally and structurally important residues within the retinal binding pocket could not be resolved/assigned or did not show chemical shift differences. Most surprisingly, even the Schiff base nitrogen ^{15}N chemical shift did not change between GPR and BPR. Therefore, only an incomplete, qualitative picture was obtained at that time. Therefore, the data set was systematically expanded in the study presented here with the aim of linking NMR data, 3D structures and optical properties via quantum chemical calculations.

We have applied both high-field (S3) and DNP-enhanced MAS ssNMR (S4) spectroscopy for mapping the structure of the retinal chromophore, the retinal binding pocket, the pSB-counter ion and retinal protein interactions in order to decode the molecular basis of the color difference between green and blue proteorhodopsin (GPR, BPR). The obtained NMR data have been used as target parameters for a high-resolution structural refinement of the chromophore and its environment based on AF-QM/MM-based simulations. The refined structures have then been used for calculating the color difference between BPR and GPR via TD-DFT calculations and for disentangling the contributing factors (S5) along with supporting experimental data (Text S18, S19, S20). Finally, the molecular basis of the GPR/BPR color switching based on the L105Q mutation has been analyzed in the context of the PR family evolution and with respect to other microbial rhodopsins (S8).

A schematic overview of our integrative approaches and pipeline is shown in Fig. S1. A list of samples, isotope labelling schemes and ssNMR experiments together with corresponding Figure index is provided in Tab. S1.

Text S2: Expression, purification and reconstitution of PRs

Sample preparation was carried out as described by us previously (*15, 56, 57*) and is described in detail below for reproducibility.

The green proteorhodopsin GPR construct (UniProt Q9F7P4) from γ-proteobacterium strain EBAC31A08 (SAR86 clade) was subcloned into pET27b(+) plasmid between NdeI and XhoI sites. The C-terminal HSV-tag and its consecutive His6-tag were kept in frame for protein purification. The blue-shift mutation L105Q was introduced into GPR. Throughout this paper, we refer to this GPR L105Q mutant as BPR. In addition, I193V and N230S mutants of GPR and BPR were prepared. The corresponding constructs were purchased from Genscript.

The proteins were expressed in *E. coli* C43 strain in M9 minimal medium (22 mM KH_2PO_4, 47 mM Na_2HPO_4/K_2HPO_4, 8.5 mM NaCl, pH 7.0). The medium was supplemented with $MgSO_4$ (1 M, 2 mL stock/L medium), $CaCl_2$ (1 M, 50 µL stock/L medium), vitamin mixture (1.5 g crushed Centrum A-Zn pill extracted in 20 mL water, 2 mL stock/L medium), kanamycin (100 mg/mL, 1 mL stock/L medium) and was completed with glucose (3 g/L medium) and NH_4Cl (2 g/L medium) as the carbon and nitrogen sources. First, the inoculated LB medium (one fresh colony or one Roti-Store cryo-bead for 100 mL medium) was shaken (240 rpm) overnight at 27 - 32 °C. The cells were collected, washed with M9 buffer twice (without glucose and NH_4Cl) and then diluted into the completed M9 medium to reach the initial OD_{600} at 0.05. The culture was shaken at 220 rpm at 37 °C until OD_{600} has reached 0.6 to 0.8. The expression

of PRs was induced by adding IPTG stock solution (200 mg/mL, 1 mL stock/L culture) to reach the concentration of 0.84 mM. Retinal stock (10 mg/mL in ethanol, 200 µL/L culture) was also added at the point of induction to reach the concentration of 7 µM in the culture. For the expression of proteoopsins (apoPRs) for the later incorporation of isotope labeled retinal or retinal analogue, the retinal was not supplied during the protein expression. For amino acid-type selective labeling or reverse labeling, specific amino acids were added at OD_{600} of 0.4 and about 40 to 60 min after the addition of amino acids, the protein expression was induced by IPTG at OD_{600} 0.6. The amino acids were added as powder (low solubility amino acids) or concentrated water solution (water soluble amino acids). For the selective labeling of Trp sidechain, the precursor molecule indole was added as concentrated DMSO solution. The amounts of amino acid/precursor for selective labeling/reverse labeling were as following (for half liter medium): histidine indole 50 mg, isoleucine 115 mg, lysine 105 mg, proline 50 mg, tyrosine 85 mg, valine 115 mg. After the IPTG induction, the temperature was switched to 27 °C and the culture was shaken at 220 rpm for 12 - 16 h. The cells were harvested by centrifugation (Beckmann Avanti J-E, JLA10.500 rotor, 5000 rpm for 15 min, 4 °C). The harvested cells were suspended in precooled buffer A (50 mM Tris, 5 mM $MgCl_2$, freshly added DNAse I (Applichem, > 5000 u/mg, a few milligrams), 200 mL buffer for cells from 1L culture) and were disrupted in three passages through a continues flow cell disruptor (Constant Systems) under 1.85 kbar (27 kpsi) and at 4 °C. The cell debris was removed by centrifuge at 8000 rpm (Beckmann GS-15R, F0850 rotor, 6726 rcf) at 4 °C for 20 min. The cellular membrane fraction was collected by ultracentrifugation (42000 rpm, Ti45 rotor) and was suspended in buffer B (50 mM MES, 300 mM NaCl, 5 mM imidazole, pH 6.5) using a painting brush. The membrane fraction can be frozen and stocked at –80 °C for the further use.

For protein purification, the membrane fraction was solubilized in solubilization buffer (buffer B with 2% w/v DDM, pH 6.5, 100 mL for membrane from half liter culture) at 4 °C overnight. The insoluble materials were removed by ultracentrifugation (42000 rpm, Ti45 rotor) at 4 °C for 1 h. The supernatant was incubated with NiNTA beads (Qiagen, prewashed with solubilization buffer, 6 mL beads for 200 mL supernatant) under gentle stirring at 4 °C for 1 h. The beads loaded with proteins were then collected in a Protino gravity column (Macherey-Nagel) and washed with 5 CV (column-volume) precooled buffer C (50 mM MES, 300 mM NaCl, 50 mM imidazole, 0.15% DDM, pH 6.5). The protein was eluted with buffer C (50 mM MES, 300 mM NaCl, 500 mM imidazole, 0.05% DDM, pH 7.5) and the concentration was determined from the retinal absorption using $\varepsilon_{retinal} = 1.81\ mL\cdot mg^{-1}\cdot cm^{-1}$, which was determined following a reported protocol (*67*).

The ^{13}C-labelled *all-trans* retinals ($^{13}C_{10-18}$-retinal and $^{13}C_{12,13,20}$-retinal) were synthesized as reported previously (*25, 59*). The ^{13}C-labeled retinals as well as 3,4-dehydro-retinal (*3,4-deH$_2$* Ret, BioSynth) were incorporated into proteoopsin in *E. coli* membrane suspension at room temperature following the previously published protocol (*25, 57*). The retinal incorporation in lipid environment rather than in detergent maintains the folding integrity and stability of PRs. For the purification of PRs with the post-expression incorporated retinal, an extensive washing step with at least 10 CV buffer C was required to fully remove the excess retinal.

The purified protein was reconstituted into DMPC/DMPA liposomes (9:1, 200 µm) in buffer D (50 mM MES, 100 mM NaCl, pH 7.0). The eluted protein was added into DMPC/DMPA stock (4 mg/mL) drop by drop to reach the final lipid-to-protein ratio (LPR, w/w) 1:2. The mixture was incubated at room temperature for 30 min. Prewashed and autoclaved biobeads (BioRad) were then added (80 mg beads/mL solution) and the mixture was incubated at room temperature for 2 h with gentle rotation. Another batch of biobeads (80 mg beads/mL solution) were added and the mixture were incubated for another 2 h at room temperature or at 4 °C

overnight. Subsequently, the third batch of biobeads (160 mg beads/mL solution) was loaded and the mixture was incubated for another 2 - 4 hours. Biobeads were then removed via a cell drainer (100 µm, Fisher Scientific) and the turbid proteoliposome suspension was subjected to ultracentrifugation (Beckmann 70Ti rotor, 55000 rpm, 1 h). The pellet was resuspended in 1.5 mL NMR buffer (50 mM Tris, 5 mg $MgCl_2$, pH 9.0) and collected again by ultracentrifugation (Beckmann 70Ti rotor, 55000 rpm, 1 h). This washing process was repeated at least twice. For the high-field ssNMR experiments (S3), the pellet was packed directly into a 3.2 mm ZrO_2 rotor by bench-top centrifuge (15000 g). For H/D exchange, the PR proteoliposome (about 30 mg) was washed in 1.5 mL NMR buffer (D_2O) twice and was then suspended in D_2O buffer again and was stirred under light illumination at 4 °C overnight. The pellet was then collected by ultracentrifugation and packed into the NMR rotor. The H/D exchanged samples were stored in D_2O buffer in sealed Eppendorf tubes at 4 °C. For VFMAS ssNMR experiments (S3), GdDOTA (Macrocyclics, 100 mM stock solution in water) was added to reach the final concentration of 0.1 mM for reducing the ^{1}H T1. The pellet was transferred into a 1.3 mm rotor using a customized ultracentrifuge packing device (*58*). The sample was sealed at the top with a rubber plug. For DNP-enhanced ssNMR experiments (S4), the proteoliposome pellet was incubated with AMUPol solution (20 mM AMUPol, 7/3 (v/v) NMR buffer/glycerol mixture, 200 µL for about 30 µL pellet) at 4 °C for 12 -16 h. No deuterated components were used for the DNP matrix in our sample preparations (*25*), which prevents the partial deuteration of pSB site.

For DNP ssNMR experiments on PRs in native *E. coli* membranes, the membrane fraction obtained from 150 mL culture was first loaded with retinal and then washed intensively with methyl β-cyclodextrin (50 mM, ca. 67 mg/mL, Glentham life science) to remove the free retinal. The membrane was then washed three times in NMR buffer (1.5 mL per wash) and was incubated with AMUPol solution as described previously. The amount of PR in native membrane packed into one 3.2 mm rotor was estimated to be less than 0.5 mg. All DNP ssNMR samples were stored at –80 °C.

High-field room-temperature MAS ssNMR experiments of retinal chromophore

Text S3: Retinal ^{13}C chemical shifts

In order to probe the retinal structure, the chemical shifts of the retinal carbons C10-C18 were determined by recording 2D ^{13}C-^{13}C proton-driven spin diffusion (PDSD) spectra (*68*) of $^{13}C_{10\text{-}18}$-retinal reconstituted into GPR and BPR (Figs. 2d, S2).

Spectra were acquired on a Bruker Avance III wide-bore 850 MHz (^{1}H Larmor frequency) spectrometer. All PDSD spectra were acquired under 14 kHz MAS and at 290 K (estimated sample temperature) on a Bruker 3.2 mm HCN probehead. About 10 mg of protein was packed into a 3.2 mm ZrO_2 rotor. The ^{1}H-^{13}C CP transfer (800 µs) was achieved by applying ramped ^{1}H (80% to 100% power) and continuous-wave (CW) ^{13}C spin-lock pulses at the field strength 63.5 (100%) and 46.4 kHz respectively. Detection took place under high-power SPINAL64 ^{1}H decoupling (83 kHz) (*69*). Mixing times of 10, 20 and 800 ms were used. All 2D PDSD spectra were acquired with 2560 (F2) and 512 (F1) points covering the spectral window of 300 (F2) and 327 (F1, 5*ω_r) ppm respectively. The spectra were processed with an 8192 (F2) by 2048 (F1) matrix. Qsine (ssb = 4) and Gaussian (lb = - 20 Hz, gb = 0.1) window functions were applied to the indirect and direct dimension respectively. Besides of the polyene carbons C10-C15, the ionone ring methyl carbons C16,17,18 can also be resolved in the 2D ^{13}C-^{13}C PDSD spectra (*25*).

All retinal ^{13}C chemical shifts are summarized in Tab. S2. Chemical shift differences between GPR and BPR are plotted in Fig. 2e.

Text S4: Retinal ^{1}H chemical shifts

The retinal hydrogen atoms are located at the retinal-protein interface and therefore sense the protein environment more directly than the retinal carbons. We have therefore also determined the chemical shifts of the retinal protons H10-H15 in GPR and BPR via ^{13}C-^{1}H HETCOR experiments as well as using direct proton detection based on very fast MAS (VFMAS).

^{13}C-^{1}H HETCOR spectra of [$^{13}C_{10\text{-}18}$-ret, U-^{15}N]-GPR and -BPR are shown in Fig. S3. Spectra were recorded on a Bruker Avance III wide-bore ssNMR spectrometer operating at 850 MHz (^{1}H Larmor frequency). Short ^{1}H-^{13}C CP (100 μs) has been used for the selective short-distance magnetization transfer from ^{1}H to the direct bonded ^{13}C. The ^{1}H and ^{13}C RF powers for CP transfer are same as those used for the PDSD experiments shown in Fig. S2. High-power ^{1}H homodecoupling (frequency-switched Lee-Goldburg, FSLG decoupling, 90 kHz) was used for suppressing ^{1}H-^{1}H dipolar coupling and for improving the ^{1}H resolution during ^{1}H chemical shift evolution period at moderate MAS (14 kHz). Each *t1* increment contains 6 pairs of ^{1}H decoupling pulses (9.07 μs) with offsets switching between +63.6 and – 63.6 kHz. The standard ^{1}H chemical shift scaling factor (0.578) was taken for the data processing. High-power SPINAL64 ^{1}H decoupling (83 kHz) has been applied for FID recording. The HETCOR spectra have been recorded with 2048 (F2) and 128 (F1) points covering the spectral window of 399 (F2) and 18.7 (F1) ppm respectively. The spectra were processed with a 4096 (F2) * 512 (F1) matrix. Gaussian (lb = -20 Hz, gb = 0.02) and Qsine (ssb = 4) window functions have been applied for F2 and F1 dimensions respectively. As shown in Fig. S3, all five ^{1}H-^{13}C pairs of retinal carbins C10-C15 are well-resolved. The ^{1}H resolution of the spectra processed with the window function specified above is about 0.2 – 0.4 ppm (170 to 340 Hz). Chemical shifts and chemical shift differences are summarized in Tab. S3.

The ^{1}H chemical shift in ^{1}H-^{13}C FSLG HETCOR experiments is scaled by a factor, which also depends on the frequency offset. Since the accurate ^{1}H chemical shifts are required for the AF QM/MM refinement in this work, we have validated these chemical shifts by additional direct ^{1}H detected experiments based on very fast MAS (VFMAS) NMR. ^{1}H-^{13}C dipolar HSQC spectra of [$^{13}C_{10\text{-}18}$-ret, U-^{15}N]-GPR and -BPR (Fig. 2d) were acquired on a Bruker Avance III narrow-bore 800 MHz (^{1}H Larmor frequency) spectrometer with a 1.3 mm VFMAS HCN probehead using a 60 kHz MAS rate. The sample has been cooled by gas flow set to 250 K and the real sample temperature under VFMAS is estimated to be about 300 K. About 2 mg protein were packed into a 1.3 mm rotor using an ultracentrifugation device (*58*). ^{1}H-^{13}C CP was achieved by applying ramped ^{1}H (70-100%, 50 kHz at 100%) and ^{13}C (CW, 15 kHz) spin-lock pulses for 1.1 ms. A double quantum (DQ) CP matching condition was used in these VFMAS experiments, which required less RF power than the zero-quantum (ZQ) CP matching condition. This is followed by a ^{13}C chemical shift evolution (*t1*) period, during which the low-power ^{1}H heteronuclear decoupling (slTPPM, 15 kHz) was applied. A ^{15}N π-pulse (8 μs) is also applied in the middle of *t1* period. The ^{13}C-^{1}H CP is achieved by the same RF power used for ^{1}H-^{13}C CP, with the exception that the duration is shortened to 200 μs in order to improve the selectivity of transfer. Water suppression is achieved via a MISSISSIPPI pulse train (without gradient) composed of four ^{1}H saturation pulses (50 μs each, 15 kHz) with alternating phases (XYXY) applied before the ^{1}H-^{13}C CP transfer step. No ^{1}H decoupling was applied during data acquisition. All 2D HSQC spectra were acquired with 128 (F1, ^{13}C) by 1024 (F2, ^{1}H) points for the spectral windows of 30 (F1) and 224 (F2) ppm. The spectra were processed with a 1024 (F1) by 2048 (F2) matrix. Qsine (ssb = 4) and Gaussian (lb = -20 Hz, gb = 0.04) window functions were applied to the indirect and direct dimension respectively. The ^{13}C chemical shifts were referenced to DSS indirectly via an adamantane sample. The ^{1}H

chemical shifts were then referenced indirectly to DSS by scaling the referenced ^{13}C “spectrometer frequency” using the conversion factor 0.251449530 (*70*). The ^{1}H resolution of the spectra processed with the window function specified above is in the range of 0.33 to 0.78 ppm (260 to 620 Hz), which already permits to resolve all five ^{13}C-^{1}H pairs on the labeled retinal polyene chain. The comparison of the results obtained by HETCOR and HSQC are summarized in Tab. S3.

Chemical shifts and chemical shift differences are summarized in Tab. S3 and compared to the data obtained from the ^{1}H-^{13}C HETCOR experiments. Overall, a very good agreement is achieved. Only the BPR-GPR chemical shift differences of H11 and H12 differ by 0.1 ppm between both methods. The ^{1}H chemical shift differences between GPR and BPR are plotted in Fig. 2e.

Text S5: Retinal-water contact

For probing the contact between water-exchangeable protons and retinal chromophore, ^{1}H-^{13}C HECTOR spectra were acquired on [$^{13}C_{10\text{-}18}$-ret, U-^{15}N]-GPR and -BPR. The acquisitions and processing parameters were the same as described in Text S3 with the exception that ^{1}H-^{13}C CP contact time was set to 800 µs. The H/D exchange protocol is described in Text S2. The resulting spectra can be found in Fig. 3e.

<u>DNP-enhanced ssNMR experiments of retinal chromophore and retinal binding pocket</u>

Text S6: General setup of DNP-enhanced ssNMR experiments

The ssNMR experiments described in Texts S3 - S5 above were recorded on non-frozen samples at high magnetic fields and provided ^{1}H and ^{13}C retinal chemical shift differences between GPR and BPR. The possibility to enhance the detection sensitivity by dynamic nuclear polarization (DNP) and to work under cryogenic conditions (under which protein dynamics and proton exchange process are quenched) enabled the recording of these additional data:

- ^{15}N CSA of the Schiff base nitrogen in GPR and BPR (Text S7)
- ^{1}H chemical shift of the pSB proton in GPR and BPR (Text S8)
- ^{13}C CSA of retinal carbons in GPR and BPR and retinal ^{13}C chemical shifts in PRs in native membranes (Text S9)
- hetNOE on the C18 and C20 retinal methyl groups in GPR and BPR (Text S10)
- Chemical shift changes within retinal binding pocket residues (Text S11)

All DNP-enhanced MAS ssNMR spectra were acquired on a Bruker Avance II spectrometer operating at 400.197 MHz (^{1}H Larmor frequency, 9.4 T). The high-power microwave beam was generated by a CPI (Communication and Power Industries) gyrotron operating at 263.580 GHz (microwave frequency). The microwave beam was directed to the DNP ssNMR probehead via corrugated waveguides. The effective microwave power input into the NMR stator was above 15 W. All DNP ssNMR samples were packed into 3.2 mm ZrO_2 rotors and were sealed with Vespel caps. In general, we found that both thin wall ZrO_2 or Sapphire rotors performed well in terms of DNP ssNMR sensitivity. All DNP ssNMR experiments presented below were carried out using 8 kHz MAS sample spinning at about 110 K. A 3 s interscan delay for all DNP ssNMR experiments was used. Unless described specifically, the following CP matching conditions applied: The ^{1}H-^{15}N CP is achieved by applying ramped ^{1}H (80-100%, max. RF strength 74 kHz) and CW ^{15}N (RF strength 50 kHz) spin-lock pulses for 1.2 ms. The ^{1}H-^{13}C CP is achieved by applying ramped ^{1}H (80-100%, max. RF strength 53.1 kHz) and CW ^{13}C (RF strength 49.6 kHz) spin-lock pulses for 800 µs. For the

^{13}C-^{15}N DCP transfer (6 ms), CW ^{13}C (28.6 kHz) and ramped ^{15}N (90-100%, 25.6 kHz) pulses are applied under CW ^{1}H heteronuclear decoupling (100 kHz). For the ^{15}N-^{13}C DCP transfer (6.4 ms to 25 ms), ramped ^{13}C (90-100%, 28.0 kHz) and CW ^{15}N (41 kHz) pulses are applied under CW ^{1}H heteronuclear decoupling (100 kHz). The ^{15}N-^{13}C DNP contact time is set to 6.0-6.4 ms and 25 ms for the short-distance (directly bonded ^{15}N-^{13}C pair, see Text S9c, S11b, S11c, S11f) and the long-distance transfer (see S11a, S11g) respectively. For all DNP experiments, high-power ^{1}H heteronuclear decoupling (SPINAL64, 100 kHz) is applied during acquisition. Other specific experimental setups can be found in the respective sections. For chemical shift calibration, an alanine sample is used as the external reference (C' chemical shift 180.3 ppm at 110 K as referenced to DSS at room temperature). The ^{15}N chemical shifts are then referenced indirectly to liquid ammonia by scaling the referenced ^{13}C spectrometer frequency with the conversion factor 0.402979940 (*70*). The acquisition and processing parameters for DNP ssNMR experiments are summarized in Tab. S4.

Text S7: ^{15}N CSA of pSB nitrogen in PRs

It was demonstrated by us before, that the isotropic ^{15}N chemical shift of the protonated Schiff base nitrogen is identical in GPR and BPR (*15*). This finding was surprising, since the adjacent retinal carbon C15 differs substantially between both PRs (Fig. 2e). We have therefore determined the ^{15}N CSA of the protonated Schiff base nitrogen, in order to gain a better insight into its electronic environment.

[$^{13}C_{10\text{-}18}$-ret, U-^{15}N]-PR samples were prepared in a non-deuterated DNP matrix in order to avoid a mixture of protonated and deuterated pSB species. It has been reported before that the pSB ^{15}N chemical shift determined under cryogenic DNP condition is equal to room-temperature MAS ssNMR measurements (*25*). A structural impact by sample freezing or usage of a DNP matrix can therefore be neglected. The pSB ^{15}N resonance is found at 182.1 ppm, which overlaps with the His sidechain ^{15}N signals under cryogenic conditions. Since the retinal $^{13}C_{15}$–pSB ^{15}N is the only isotope labeled carbon-nitrogen pair in this labeling scheme, a ^{13}C-^{15}N DCP filter can be used to specifically select the pSB ^{15}N resonance. The transversal, DCP-filtered ^{15}N magnetization is then flipped into longitudinal magnetization followed by CSA recoupling. ^{15}N CSA recoupling is achieved using the $RN10_1^4$ scheme (*29*) (40 kHz, 5*ω_r) under CW high-power ^{1}H decoupling (100 kHz) with a centered ^{13}C π-pulse for refocusing the ^{13}C-^{15}N dipolar modulation during the CSA recoupling period (Fig. S4a). The $RN10_1^4$ sequence was chosen due to the relatively large CSA scaling factor (0.446) of this σ_1-recoupling scheme ($m = \pm 1$). It also recouples homonuclear dipolar couplings, which are however negligible here for the pSB ^{15}N where the strongest ^{15}N-^{15}N dipolar coupling is only 14 Hz (4.5 Å). The DCP-filtered ^{15}N RNCSA spectra are acquired in the form of 2D CSA-resolved ^{15}N NMR spectra. The CSA evolution is encoded by 48 hypercomplex (States) points, which corresponds to 24 increments in modulation time as only the cosinusoidal part of the signal (every second FID) is acquired. Each F1 increment contains 10 pairs of R-symmetry pulses. The recoupled CSA patterns are presented in Fig. 2c and Fig. S4b. The spectra appear symmetric due to recording of the cosinusoidal part of the data set only. Therefore, only the value but not the sign of the CSA value can be determined.

The recoupled CSA patterns were extracted as projection from the 2D CSA-resolved ^{15}N spectra and analyzed using SIMPSON (*71, 72*) in order to determine the chemical shift anisotropy and the asymmetry parameter. The parameters were scanned by minimizing χ^2 to find the best fit. The zero-frequency "signal", which originates partially from the non-recoupled components, was excluded from the data fitting in order to minimize the bias. It is noteworthy that, different from the SUPER experiment (*73*), the isotropic chemical shift does not contribute

to the recoupled CSA pattern. Therefore, the CSA spectrum is centered at zero-frequency without spectral shearing, which simplifies data analysis. The errors of the fitting values were defined from the tolerance of χ^2 values and were further converted to the errors in CSA principal values.

A comparison of fitted and experimental CSA patterns is shown in Fig. S5a,b and fitting results are summarized in Tab. S2. The CSA tensor data of GPR agree with those previously reported based on slow MAS spinning sideband analysis (*74*). Notably, the ^{15}N δ_{11} of GPR (323.9 ± 2.8 ppm) and BPR (320.2 ± 2.3 ppm) are more deshieleded (> 30 ppm) than δ_{11} in BR (287 ppm for bR568 as referenced to liquid ammonia) (*75*), which is in line with the stronger N-H bond of pSB in PRs due to the absence of a tightly coordinated water molecule.

Text S8: ^{1}H chemical shift of pSB hydrogen in PRs

We have further complemented the characterization of the pSB site by determining chemical shift of pSB hydrogen. This proton was detected via its correlation with the pSB nitrogen in 2D ^{1}H-^{15}N HETCOR spectra under DNP condition.

Experiments here were carried out on [U-^{13}C, ^{15}Nε-Lys]-PRs. High-power ^{1}H FSLG homonuclear decoupling (*76*) (100 kHz) was applied during ^{1}H chemical shift evolution. The same ^{1}H-^{15}N CP matching condition as described above was used with the exception that the CP contact time was reduced to 50 µs to minimize non-specific transfer. The theoretical chemical shift scaling factor (0.578) was used for determining the ^{1}H chemical shifts. They were calibrated via ^{1}H-^{13}C HETCOR spectra recorded on the same sample.

The ^{1}H-^{15}N HETCOR spectra of [U-^{13}C, ^{15}Nε-Lys]-PRs are shown in Fig. S6. The pSB proton shifts by -0.8 ppm from GPR to BPR. This shift depends on the exact knowledge of the FSLG scaling factor and is considered here as representation of a semi-quantitative trend. Its ^{1}H linewidth is about 920 Hz (2.3 ppm), which could be due to the limited magnetic field (9.4 T) and/or local structural heterogeneity of pSB proton site. The chemical shifts are given in Tab. S2 and chemical shift differences are plotted in Fig. 2e.

Text S9: Retinal ^{13}C CSA and control experiments in native membranes

In addition to the determination of ^{13}C chemical shift differences of retinal carbons C10-C15 between GPR and BPR as described in Text S3, ^{13}C CSA values have been also determined in this work. The ^{13}C CSA parameters are sensitive to the electronic structure of the retinal π-conjugation system. Therefore, these parameters provide the valuable high-resolution access to the molecular orbitals of retinal chromophore that is directly related to the light absorption behaviors. For this purpose, the isotropic retinal carbon ^{13}C chemical shifts under cryogenic DNP conditions have first been validated. These measurements were complemented by chemical shift determinations in native cellular membranes. The ^{13}C CSA measurements were based on CSA recoupling experiments similar to those described above for the ^{15}N CSA of the Schiff base nitrogen.

(a) ^{13}C chemical shifts of retinal in PRs under DNP condition

For validating the ^{13}C chemical shifts of retinal carbons C10-C15, 2D ^{13}C-^{13}C DQ-SQ spectra (*77*) on [^{13}C$_{10\text{-}18}$-ret, U-^{15}N]-PRs were recorded. DQ excitation and reconversion were achieved using the POST-C7 scheme (*78*) (56 kHz, 14 units, 0.5 ms) as described in previously (*15*). The spectral window of the indirect dimension is set to 5*ω_r (56 kHz). As shown in Fig. S7, all ^{13}C chemical shifts of these retinal polyene carbons are consistent with those obtained at room temperature. The DNP-specific experimental conditions (low temperature, glass-forming matrix) have therefore a negligible effect on the retinal structure in these proteins. The spectrum is shown in Fig. S7b.

(b) ^{13}C chemical shifts of retinal in PRs in native cellular membranes

Our ^{13}C and ^{15}N experiments on retinal carbons and Schiffbase nitrogen demonstrate that the glass matrix and the low temperature used for DNP experiments do not alter the active site of GPR and BPR. Here, we further show that the synthetic lipid environment used for our sample preparation does not pose additional structural perturbations compared to the native bacterial membrane. We have therefore recorded ^{13}C spectra of [$^{13}C_{10\text{-}18}$-ret, U-^{15}N]-PRs in non-purified inner *E. coli* membrane preparations. We have focused on the retinal structure as it is highly sensitive to environmental changes and is directly linked to the color phenotype studied in this work.

Sensitivity enhancement based on DNP has been essential to overcome the detection limit of retinal in PR in native cellular membranes. The sample preparation protocol for the native cellular membrane with overexpressed PRs can be found in Text S2. Here we choose *E. coli* as the host bacterium for the in-situ ssNMR study as it is also gram-negative bacterium as γ-bacteria, the main native hosts of PR, and therefore is expected to have the similar lipid composition. It was also shown preeviously that recombinantly expressed proteorhodopsin is fully functional in *E. coli* (*79*). $^{13}C_{10\text{-}18}$ retinal. Isotope labeled $^{13}C_{10\text{-}18}$ retinal, which binds covalently and selectively to proteoopsin, has been added to native membranes. The cellular membrane pellet was incubated in DNP solution in the same way as the proteoliposome samples (*15*). The DNP enhancement (35 folds) was comparable proteoliposome samples (40-60 folds) and enabled the acqusition of 2D ^{13}C-^{13}C DQ-SQ spectra of the retinal chromophore in GPR and BPC in native *E. coli* membrane on less than 0.5 mg of protein.

As shown in Fig. S7a and b, the resolved ^{13}C resonances match those obtained on GPR and BPR embedded in synthetic lipids (DMPC/DMPA). These results support the use of synthetic lipids for investigating the structure of retinal region in PRs.

(c) ^{13}C CSA of retinal polyene carbons in PRs

The ^{13}C CSA data of the retinal carbons C10-C15 were determined by ^{13}C RNCSA recoupling using samples [$^{13}C_{10\text{-}18}$-ret, U-^{15}N]-G/BPR and [$^{13}C_{12,13,20}$-ret, U-^{15}N]-G/BPR. Due to the strong ^{13}C-^{13}C homonuclear dipolar coupling in the uniformly ^{13}C-labeled retinal C10-C15 segment (up to 3.2 kHz for a 1.34 Å ^{13}C-^{13}C pair), a σ_2 CSA recoupling scheme is required, which does not actively recouple the homonuclear dipolar interactions. Therefore, the $R12_1^4$ (*29*) scheme was selected due to its high scaling factor (0.310) and because of its moderate ^{13}C RF strength requirements (48 kHz, $6^*\omega_r$). High power CW ^{1}H decoupling has been applied during the CSA recoupling period. The ^{13}C-^{15}N dipolar coupling was removed by applying a ^{15}N π-pulse. Due to the overlapping of retinal polyene ^{13}C signals with natural abundance ^{13}C signals of aromatic sidechains in PR, a POST-C7 DQ-filter (14 POST-C7 units, 0.5 ms) was applied *prior to* the RNCSA recoupling scheme (see Fig. S4c for the full pulse sequence).

In this way, the CSA pattern of C11 and C14 could be resolved (Fig. S4e,f). The C10/C12 resonances overlap and are hardly affected by the L105Q green/blue mutation. Therefore, the CSA patterns of these two carbons were analyzed together (Fig. S4d).

The retinal C13 and C15 signals partially overlap. For resolving these two carbons for the CSA measurement, we have applied two complimentary approaches. First, we have applied a ^{15}N-^{13}C DCP filter to select the C15 signal as the retinal C15 – pSB ^{15}N is the only ^{13}C-^{15}N in [$^{13}C_{10\text{-}18}$-ret, U-^{15}N]-G/BPR (Fig. S4h). In this way, the C15 CSA pattern could be extracted (Fig. 2b, S4i). Second, we have used another retinal labeling scheme, namely [$^{13}C_{12,13,20}$-ret, U-^{15}N]-G/BPR, in which the C13 but not the C15 carbon is labeled. With the pulse sequence described above (Fig. S4c) the C13 CSA pattern was obtained (Fig. S4f). The acquisition and processing parameters are summarized in Tab. S4.

The analysis of the recoupled CSA lineshapes of retinal carbons C10-C15 was carried out as described above for the ^{15}N CSA of the pSB nitrogen. Known CSA values determined on BR(*80, 81*) were used as starting values for data fitting. A comparison of simulated and experimental CSA patterns is shown in Fig. S5c-I. The fitting results are summarized in Tab. S2.

As for the ^{15}N CSA experiments, the recoupled Hamiltonian results in a symmetric lineshape from which only the value but not the sign of the CSA can be determined. This did not trouble our further data analysis using AF QM/MM simulations (S5.1) as one set of values can be easily excluded. In addition, all the reasonable signs of CSA of all polyene carbons selected by AF QM/MM match well with the experimental values reported on retinal derivatives (*82*).

Text S10: hetNOE DNP build-up kinetics of retinal C18 and C20 methyl groups

Retinal methyl groups offer unique opportunities for probing their protein environment by specific DNP techniques. Previously, we have shown for GPR that both the chemical shift and the heteronuclear Overhauser effect (hNOE) build-up kinetics of the retinal C20 methyl group reports on local changes induced by the photoisomerization of the nearby retinal C13=C14 double bond (*25*). We have also probed the distal mutation-induced local protein structural changes around the C20 methyl group in 11-cis retinal in visual rhodopsin via the C20 chemical shift (*83*).

Here, the C20 methyl group is found in close proximity to key residue L105/GPR or Q105/BPR, respectively. We have probed C20 via hetNOE build-up kinetics on [$^{13}C_{12,13,20}$-ret, U-^{15}N]-PRs. The C20 hetNOE build-up times were determined as described previously (*25*). In these experiments, also the ^{13}C chemical shifts of C20 was determined. All these measurements were performed using an 8 kHz MAS rate at 110 K. Only small chemical shift differences between GPR (16.2 ppm) and BPR (15.7 ppm) and similar hetNOE built-up times were observed (Fig. S8, GPR: 2.9 $\pm$ 0.1 s, BPR: 3.2 s $\pm$ 0.1 s). These data show that the color-switching residue Leu/Gln105 does not pose a strong direct steric effect on this methyl group.

We have extended this approach to the C18 methyl group in the retinal ionone ring in [$^{13}C_{10\text{-}18}$-ret, U-^{15}N]-G/BPR in order to probe differences in ring-protein interactions between GPR and BPR. The same experimental conditions were used as for C20. As shown in Fig. 3h, the C18 hetNOE build-up times differ between GPR (2.1 $\pm$ 0.1 s) and BPR (1.4 $\pm$ 0.1 s), demonstrating alterations in the molecular packing around this methyl group.

Text S11: DNP-enhanced ssNMR mapping of retinal binding pocket residues

Besides chemical shift differences in retinal chromophore and Schiff base between GPR and BPR, also L105Q-mutation-induced effects on adjacent residues need to be resolved. In our previous work (*15*) and based on the known backbone assignment of GPR (*84, 85*) chemical shift changes had been identified for a number of residues using uniformly labelled samples. However, many functionally important sidechains could not be resolved or assigned in these samples. Therefore, we expanded this database utilizing amino-acid selective labelling schemes and spectroscopic filtering methods either based on unique pair labelling or based on specific through-space residue-residue or retinal-protein contacts. The later involved a prior structural knowledge from the 3D BPR X-ray structure 4JQ6 (*17*).

(a) pSB nitrogen – counter ion (D97 and D227 Cγ) interactions

The counter ion - pSB complex is an important factor for color tuning in many retinal proteins. In G/BPR the counter ion is formed by the primary proton acceptor Asp97 and by

Asp227. Both residues are located close to the Schiff base nitrogen. Here, we tried to determine chemical shift differences of Cγ in Asp97 and Asp227 between GPR and BPR. For this purpose, [U-^{13}C, ^{15}Nε-Lys]-G/BPR samples were prepared with the aim to spectroscopically select Asp-Cγ via magnetization transfer from the Schiff base nitrogen. This approach appeared more feasible than ^{13}C-labelling of aspartates, which is not very efficient due to isotope scrambling. Magnetization transfer between Cγ of Asp97/227 and the pSB nitrogen was then established via a very long DCP step to account for the relatively long distance / weak dipole coupling between Asp-Cγ and pSB-N (3.6 - 5 Å). The pSB ^{15}N nitrogen has no directly bonded ^{13}C carbon in [U-^{13}C, ^{15}Nε-Lys]-G/BPR, which also aids this long range DCP transfer step.

The experimental setup for ^{1}H-^{15}N CP and ^{15}N-^{13}C DCP are as that described in Text S6. DCP contact time is set to 25 ms. The ^{13}C and ^{15}N offsets are set to 180 and 185 ppm respectively for promoting the magnetization transfer from pSB nitrogen to the Asp sidechain carboxylate carbons. The acquisition and processing parameters can be found in Tab. S4. In our hands, DCP worked more efficiently than TEDOR in this specific case.

^{15}N-^{13}C DCP spectra of [U-^{13}C, ^{15}Nε-Lys]-G/BPR are shown in Fig. S9a. Both D97- and D227-Cγ signals can be resolved via their correlation with the pSB ^{15}N resonance. They are assigned based on previous studies (*15, 86*). The chemical shift differences of both sites between GPR and BPR are negligible. In addition, natural abundance correlations with retinal carbon C15 and K231 Cε as well as with labelled W98 Cδ1 can be observed. DCP spectra of [U-^{13}C, U-^{15}N, rev-His]-G/BPR confirm the assignment of the pSB N - K231 Cε cross peaks Fig. S9b). Reverse labelling of His in these samples was necessary since the protonated nitrogens in the His sidechain resonate close to the pSB ^{15}N signal, which would have caused spectral overlap on the spectra. The data show, in contrast to the ^{15}N pSB chemical shifts, a clear difference between GPR and BPR (Fig. S9b). Chemical shift differences are summarized in Tab. S6.

(b) Schiff base residue Lys231

The retinal is covalently linked to GPR/BPR via Lys231. We have therefore probed the ^{13}C chemical shifts in the Lys sidechain as these might respond to differences in the retinal binding pose within both rhodopsins. The Lys231 signals were selectively filtered from the ^{13}C background of the uniformly labelled proteins via magnetization transfer from the Schiff base nitrogen (Lys231 Nζ). Since its chemical shift is similar to the protonated nitrogens in the His sidechains, reverse labelling of His was applied ([U-^{13}C, U-^{15}N, rev-His]-G/BPR).

The correlation between Lys231 Nε and the adjacent Cδ was established via a DCP experiment on [U-^{13}C, U-^{15}N, rev-His]-G/BPR. The ^{15}N and ^{13}C offsets for the DCP pulses are set to 183 and 47 ppm respectively in order to be on-resonance for ^{15}Nζ and ^{13}Cε. A DCP contact time of 5.6 ms was used. The DCP spectrum in Fig. S10a shows the a single ^{15}Nζ - ^{13}Cε cross peak. To reach also the other carbons along the chain, an additional PDSD step (100 ms) was used. The ^{15}N-^{13}C Nζ(Cε)CX are shown in Fig. S10b. All Lys231 carbons can be detected. The short range DCP experiment in Fig. S10a allows here to identify unambiguously the ^{15}Nζ - ^{13}Cε cross peak.

The spectra in Fig. S10 reveal small differences between GPR and BPR except for Lys231 C'. The assigned chemical shift differences can be found in Tab. S6.

(c) Tyr200 and Pro201

Tyr200 is located close to the retinal C20 methyl group. We have shown before for GPR that Tyr200 can be selective detected via magnetization transfer from the adjacent retinal

C20 methyl group via hetNOE under DNP and a subsequent spin diffusion step (SIMPLE, *S*elective detection of *I*nternuclear contacts by *M*ethyl *P*o*L*arization *E*nhancement, Fig. S11a, b) (*25*). Here, this approach was applied to samples of [$^{13}C_{12,13,20}$-ret, U-^{13}C Tyr]-G/BPR. These spectra were acquired using the standard setups described in reference (*25*) with 1.0 s build-up time. As shown in Fig. S11d small chemical shift differences on Tyr sidechain carbons can be detected. They are also resolved in 2D N(CO)CX 2D spectra of unique pair-labelled [U-^{13}C Tyr, ^{15}N-Pro]-G/BPR. The chemical shift differences are summarized in Tab. S6.

(d) Trp98 sidechain

The retinal chromophore is sandwiched by Trp98 and Trp197 in the binding pocket. Here, we have determined ^{15}N chemical shift differences of the indole nitrogen in these residues between GPR and BPR by utilizing dipolar couplings between ^{13}C retinal carbons and ^{15}N Trp nitrogens in [$^{13}C_{12,13,20}$-ret, U-^{15}N]-PRs.

The Trp98 indole nitrogen was detected via dipolar correlation with nearby retinal polyene carbons C13, C14, C15 by 2D ^{15}N-^{13}C TEDOR experiments on [$^{13}C_{10\text{-}18}$-ret, U-^{15}N]-PRs. To avoid natural abundance cross peaks, an additional double quantum filter for natural abundance suppression was used (Fig. S12a,b). A TEDOR transfer of 5 ms total recoupling time (throughout the whole pulse program) and a POST-C7 DQ-filter composed by 14 POST units for DQ excitation and reconversion (56 kHz, 0.5 ms), respectively were applied. Spectra are shown in Fig. S12c,d. The TEDOR spectrum (Fig. S12c) shows a strong intra-residue ^{15}N-^{13}C correlation originating from natural abundance (1.1%) ^{13}C on the indole ring, which overlaps with C14-Trp cross peaks. The additional DQF filter (Fig. S12d) removed these contributions. No ^{15}N chemical shift differences for Trp98 can be detected.

(e) Ile193 – Trp159 and Ile192/retinal – Trp197 contacts

For detecting Trp197, through-space correlations with the adjacent Ile192 were utilized for detecting both residues. For this purpose, ^{15}N-^{13}C TEDOR spectra of [^{13}C'-Ile,^{15}Nε-Trp]-G/BPR were recorded. Acquisition and processing parameters are listed in Tab. S4. The TEDOR mixing time was set to 20 ms.

^{15}N-^{13}C TEDOR spectra of [^{13}C'-Ile,^{15}Nε-Trp]-G/BPR are shown in Fig. S13a (same as Fig 3d). Instead of one correlation between Trp197 and Ile192, two cross peaks are observed for GPR and BPR. The assumption of close spatial proximity of Trp197 and Ile 192 is based on the BPR Xray structure PDB 4JQ6(*17*). In this PR, Ile192 is followed by Val193. In our GPR and GPR L105Q mutant used as BPR model, Val193 is replaced by Ile193. This residue is also labeled and could cause through-space correlations to adjacent residue Trp159. In order to validate this assumption, I193V mutants of GPR and BPR were prepared. Their ^{15}N-^{13}C TEDOR spectra only show one set of cross peaks (Fig. S13b), which can therefore be assigned to Trp197-Ile192. Chemical shift changes are summarized in Tab. S6. The Trp197 indole nitrogen was also detected via the dipole couplings with the nearby retinal methyl C20 carbon through ^{15}N-^{13}C TEDOR experiments on [$^{13}C_{12,13,20}$-ret, U-^{15}N]-G/BPR (Fig. S13c). A TEDOR transfer time of 15 ms was used. All acquisition and processing parameters are summarized in Tab. S4. The drastic chemical shift of Trp197 Nε1 is in line with our previous reports on the Trp197 ^{13}C chemical shifts (*15*). The same experiments were performed on samples of [^{13}C'-Ile,^{15}Nε-Trp]-G/BPR in which all-*trans* retinal was replaced by *3,4-deH$_2$*-retinal or Asn230Ser mutation was introduced (Fig. 3d).

(f) Val102 – Pro103 pair

Residues Val102 and Pro103 are located in the retinal binding pocket and form a unique pair. This pair can be selectively detected in 2D ^{15}N-^{13}C N(CO)CX spectra on samples

of [U-^{13}C Val, ^{15}N-Pro]-G/BPR. As shown in Fig. S14, these two residues do not experience detectable chemical shift changes.

Val102 and Pro103 are located in between the color-switching residue (Leu/Gln105) and counter ion Asp97. All these residues are located on helix C and as shown in Fig. S9, Asp97 is also unperturbed by the color mutant. It therefore appears that the structural impact of Leu105Gln mutation does not disrupt and propagate via the backbone of helix C towards the extracellular direction.

(g) Asn230 sidechain

The NMR detection of Asn230 based on selective pairwise labelling is challenging due to isotope scrambling of Asn. Therefore, the ^{15}Nδ2 signal of Asn230 was selected via through space correlation with the adjacent ^{13}C-labelled residue Tyr200 in samples of [U-^{13}C Tyr, U-^{15}N]-G/BPR. 2D ^{15}N-^{13}C DCP spectra were recorded with a long (25 ms) DCP contact time and are compared to spectra of [U-^{13}C Tyr, ^{15}N-Pro]-G/BPR in Fig. S17. The latter shows the ^{13}C resonances of Try200 (Fig. S17a) as described above (Fig. S15c). A number of correlations of these carbons with Asn230 Nδ2 can so be identified in [U-^{13}C Tyr, U-^{15}N]-G/BPR (Fig. S17b) demonstrating chemical shift differences between GPR and BPR. Their values are summarized in Tab. S6.

AF-QM/MM-based structural modeling and color tuning mechanism

Text S12: AF-QM/MM-based structural modeling of GPR and BPR

(a) Background and challenges of chemical shift-based structural modeling

The light absorption behaviors of retinal proteins are highly sensitive to both the retinal conformation and the structure of the protein surrounding. Therefore, high-resolution PR structures, in particular including the *atomic resolution structures* of retinal chromophore, are required for addressing the molecular mechanism of color switching pursued in this study. Such a high resolution is not routinely achievable on membrane proteins by X-ray crystallography or cryo-EM at the moment. Indeed, the limited resolution (2.3 Å) as well as some unsolved structural ambiguity in the retinal binding pocket of the previously determined X-ray crystallography structures of PRs (*17*) hamper the direct use of these data for the mechanistic investigation of color switching. In addition, these structures were determined at non-physiological acidic pH that is below the pKa of pSB counter ions. Therefore, further structural modeling is required for decoding the PR color switching.

Initially we have attempted to refine the structure of the retinal binding pocket through inter-residue and intra-residue distance restraints. Such an approach requires a comprehensive set of distances within the rather large and structurally complex retinal binding pocket, which are difficult to obtain at high-accuracy/precision. Alternatively, chemical shift data (isotropic and anisotropic chemical shifts) are sensitive indicators for fine structural changes. In particular, the CSA tensors offer a unique “3D view” of the electronic structures of molecules at the sub-atomic resolution, whereas the ^{1}H sites on the ligand sense directly the protein environment. Here, our collection of selected NMR chemical shift parameters on both retinal chromophore and its protein environment (Tabs. S6, S7) offer a starting point for AF-QM/MM-based structural modeling.

In general, chemical shift-based protein structural modeling faces six major challenges: First, chemical shift parameters (isotropic chemical shift and CSA tensor) are determined directly by the electronic structure and are therefore better to be computed by quantum chemical methods, which leads to the remarkable computational burdens for large protein systems. Second, chemical shift is fundamentally a local property and therefore is less efficient

for resolving directly global structural reorganizations. Third, the global structural complexity that can be explored by a membrane protein further escalates the computational complexity. Fourth, a sampling of local structural dynamics is required for reproducing explicitly the dynamically averaged chemical shifts. Fifth, both the chemical shifts and the protein structures are influenced by protein electrostatic fields (E-fields), which are often not treated explicitly or even not included within computation pipelines. Sixth, other types of complementary data are sometimes missing for guiding structural modeling or cross-validation.

Currently, a number of approaches have been developed for tackling some of these issues. The protein backbone chemical shifts can be predicted rapidly from protein (secondary) structures with a number of empirical methods.(*87-90*) This type of empirical approaches have even empowered the chemical shift-driven structural determination of middle sized soluble proteins.(*91*) We have also recently integrated a similar approach with Rosetta modeling to determine the structural model of GPCR-peptide complexes.(*92*) However, the protein sidechains are rather difficult to be treated empirically. Moreover, the empirical methods cannot deal with CSA tensors and are not suitable for treating ligands (e.g. retinal chromophore) embedded in complex protein environments. At the moment, QM-level calculations (on 10^2 atoms) have already succeeded in assisting the structure determination of small molecules in both solutions (*93, 94*) and solids (*95*) and have been even applied to the metal-binding residues in a metalloprotein for selecting the local high-resolution structure from a pool of available high-resolution X-ray crystallographic structural models.(*35*) However, none of these approaches can afford the computational demands for our structural modeling of PRs targeting both the fine structures of retinal chromophore and its protein environment. To address the abovementioned major challenges on our protein targets, we have turned to an emerging powerful technique, namely automated fragmentation QM/MM (AF-QM/MM), and have developed a tailored approach for the structural modeling of PR color variants based on this computational chemistry method.

(b) Rationale for AF-QM/MM calculations on PR chemical shifts

First, AF-QM/MM permits the QM-level calculation of protein chemical shift parameters even on macromolecular systems. Owing to the poor scaling of *ab initio* methods, it has been impractical to apply conventional full-system quantum chemistry methods for calculating the *ab initio* NMR chemical shifts of large proteins. Since the NMR chemical shift is fundamentally a local chemical property and is determined primarily by the local (electronic) structure rather than the entire protein structure, AF-QM/MM approach is highly suitable for the efficient *ab initio* calculations of these NMR parameters.(*19, 38, 43, 50, 61, 96-99*) In the AF-QM/MM approach, the entire protein is divided into non-overlapping *fragments* termed core regions. Usually, each amino acid is assigned as a core region. The residues within a certain range from the core region are chosen as the buffer region. Both the core region and the buffer region are treated by QM, whereas the rest of the system is described by embedding charges. The purpose of the buffer area is to introduce the local QM effects on the chemical shifts of the core region. In the AF-QM/MM approach, because a buffer region is added to smoothly link the core region and MM environment, atoms on the boundary between the QM and MM regions are relatively far from the core region and their influence is attenuated. Thanks to the presence of QM buffer region, the core region is not limited to a residue. We could assign any subgroup of a residue as a core region. Each fragment-centric QM/MM calculation is carried out separately and only the shielding constants of the atoms in the core region are extracted from the individual QM/MM calculations. Therefore, all the calculations on different fragments are mutually independent and can be parallelized, which remarkably accelerates the QM calculations on the protein. AF-QM/MM pushes the QM calculation to the linear O(N)

order/scale with rather small prefactor. This approach has opened the access for QM to large biological molecules. The GPR molecule studied in this work contains 1774 hydrogen atoms, 1191 carbon atoms, 264 nitrogen atoms and 302 oxygen atoms and 12 sulfur atoms (*about* 3.5×10^3 *atoms*). The protein molecule is embedded in 144 DMPC and 16 DMPA lipid molecules (11328 hydrogen atoms, 5680 carbon atoms, 144 nitrogen atoms, 1280 oxygen atoms and 160 phosphorous atoms) and 12000 water molecules (24000 hydrogen atoms and 12000 oxygen atoms) with 18 sodium ions and 16 chloride ions for neutralizing the entire system. The full model contains therefore *about* 6×10^4 *atoms*. As described in detail in Text S14 and showcased in Tab. S5 thereafter, the QM core region and total QM region (core and buffer) in the AF-QM/MM approach are two to three and one orders of magnitude smaller than the full size of GPR, respectively. More importantly, *the size of each QM fragment is independent from the overall protein size* because each residue can have only a limited number of residues in its vicinity. Hence, the largest fragment normally contains a few hundreds of atoms consisting of main-group elements, which is rather computationally affordable using Density Functional Theory (DFT). In this work, all DFT calculations were carried out using the GIAO method and with the B3LYP functional and the 6-31G** basis set. On a small-sized 20-core cluster (Intel Xeon E5-2685 2.6 GHz processor), the AF-QM/MM treatment of the whole GPR molecule at the specified level takes only about 5 days, which is in the drastic contrast with the extraordinarily extended CPU time required for the conventional approaches. The AF-QM/MM approach also reproduces faithfully the protein chemical shifts computed by the conventional full system QM calculations. It also outputs the chemical shift tensors, therefore could be used as the computational engine for CSA-guided structural modeling. Furthermore, solvent and lipid environments, either implicit or explicit, can be included feasibly in the model for improving the quality of chemical shift calculations by AF-QM/MM approaches. The technical details regarding AF-QM/MM calculations are described in Text S14. In general, this method can be applied feasibly and robustly to any biological molecular systems such as protein–ligand complexes, membrane protein−lipid complexes, proteins with modified/unnatural residues, metalloproteins, DNAs/RNAs and bioinspired macromolecular systems.

Second, the color tuning is mainly determined by the interplay between retinal structure and the local protein environment. Therefore, we have primarily focused on the local modeling within this region, which can be supported sufficiently by the chemical shift parameters that are sensitive to the local structures as well.

Third, the global folding of PR is unperturbed by the color switch Leu105Q.(*15*) The large-scale structural arrangement, which is still highly demanding for *ab initio* methods to depict, is not required in our structural modeling. Therefore, our structural modeling can rather focus on the retinal chromophore and its protein surrounding. This drastically reduces the structural complexity and ambiguity of the modeling process. (see S5.1e for more details)

Fourth, the low temperature used in our DNP experiments efficiently quenches most of the molecular motions, which alleviates the computational burden required for exploring the conformational space in search for the most populated structure. This structure corresponds approximately to the NMR peak maxima of frozen samples and to the maxima of light absorption spectra.

Fifth, the protein charges, both on the distal background and on the nearby local residues, are already included explicitly in the AF-QM/MM protocol. Therefore, the E-field effect is inherently taken into account in the chemical shift calculation and structural modeling in our approach (see Text S12c, S12d, S15 for more details). The external E-fields generated by membrane potential and membrane surface charges are not considered here. The membrane potential is usually less than 1 MV/cm, which is one to two orders of magnitude

smaller than the protein internal E-field and therefore shows much less direct impacts on chemical shifts compared to protein E-field. Since in this work we are primarily interested in the residues that are deeply buried in the protein interior and therefore far away from lipid head groups, the E-field by membrane surface charges are not relevant to our investigations. But to be close to our experimental situation, we have chosen simulation conditions that correspond to a pH > pKa (D97). To achieve that, hydrogen atoms were added to the protein using the Leap module in the AMBER program. The amine groups were fully protonated (Lys and Arg residues and N-terminal), and the carboxylic groups were deprotonated (Asp and Glu residues and C-terminal). As a result, D97 and D227 were deprotonated. All His residues were left neutral and protonated at the ND1 position based on the local electrostatic environment.

Sixth, in addition to the chemical shift parameters, our refined structural model has been also supported by a number of complementary computational and experimental results, including chemical shifts left out from our structural remodeling (Text S13), MD simulation of local water distribution using the compatible protein charge models (Text S14), TD-DFT calculations of light absorption wavelength (Text S16).

(c) General protocol of AF-QM/MM calculation

The isotropic chemical shifts and/or chemical shift anisotropies of various nuclei (^{1}H, ^{13}C and ^{15}N) were calculated by automated fragmentation quantum mechanics/molecular mechanics (AF-QM/MM) approach proposed by He, Wang, Merz and co-workers (*39, 50, 60, 61*). In the AF-QM/MM approach, each individual amino acid is taken as the core region. The buffer region for the *n*th core region is defined by the following criteria:

1. The sequentially connected (*n*-2)th, (*n*-1)th, (*n*+1)th and (*n*+2)th residues (neighboring residues)
2. A non-neighboring residue outside the core region with an atom less than 4 Å away from any atom in the core region (at least one of these two atoms is a non-hydrogen atom)
3. A non-neighboring residue outside the core region with a hydrogen atom less than 3 Å away from a hydrogen atom in the core region
4. A non-neighboring residue with a heavy atom on its aromatic ring less than 5 Å away from any atom in the core region.

The non-neighboring residues in the buffer region are simply capped by hydrogen atoms to saturate the dangling bonds. Both the core and the buffer regions are treated by quantum mechanics, while the rest of the protein is described using the point charge model to account for the electrostatic effect (electrostatic embedding). Each core-centric (core and buffer) QM/MM calculation is carried out separately and only the shielding constants of the atoms in the core region are extracted from individual QM/MM calculations. For Lys231-retinal moiety, the core region includes Lys231, retinal C10-C15 polyene chain segment and retinal C20 methyl group, whereas the remaining part of retinal chromophore is included in the buffer region. (Fig. S19) The size of different regions in our AF-QM/MM simulations are showcased in Tab. S5. All DFT calculations were carried out with the Gaussian 09 (*100*) package using the GIAO (*101*) method, and the density functional theory with the B3LYP functional and the 6-31G** basis set was employed.

The isotropic chemical shift ($\delta_{\rm iso}$) is obtained by taking the difference between the calculated magnetic shielding (σ) and the shielding of the same nucleus in DSS as the reference ($\sigma_{\rm ref}$):

$$\delta_{\rm iso} = \sigma_{\rm ref} - \sigma$$

where σ_{ref} = 193.75 ppm for the carbon in DSS. The anisotropic chemical shift δ_{aniso} is defined as the difference between the tensor element which is furthest away from the isotropic chemical shift (δ_{11}or δ_{33} renamed as δ_{zz}) and the isotropic chemical shift according to the Haeberlen conventions (*102*):

$$\delta_{\mathrm{aniso}} = \delta_{\mathrm{zz}} - \delta_{\mathrm{iso}}$$

The asymmetry parameter describes the deviation from axial symmetric:

$$\eta = \frac{\delta_{\mathrm{xx}} - \delta_{\mathrm{yy}}}{\Delta\delta_{\mathrm{aniso}}}$$

(d) Derivation of the polarized protein-specific charges

The polarized protein-specific charges (PPCs) were fitted to electrostatic potentials using fragment quantum mechanical calculation of the protein in implicit solvent. This charge model was implemented in both AF-QM/MM simulations (S6.3, S6.5) for generating the background charge (and therefore the E-field) and in MD simulations (S6.7) for introducing the polarization effects. The molecular fractionation with conjugate caps scheme by incorporating the Poisson-Boltzmann solvation model (MFCC-PB)(*103*) was applied to iteratively fit the atomic charges. The first step of the iterative procedure is solving the electronic structure of each individual amino acid fragment at the B3LYP/6-31G* level. In each fragment calculation, the rest atoms outside the QM region were considered as background charges. The retinal and the structural water molecules were considered as part of the side chain. In the second step, the standard two-stage RESP method(*104*) was used to fit the partial charges of protein atoms. The PB solver Delphi(*105*) was employed to calculate the induced charges on the solute-solvent interface, which was defined using a probe radius of 1.4 Å. The grid density was set to 4.0 grids/Å in numerically solving the PB equation with a solvent dielectric constant of 80. The newly fitted atomic charges were passed to the next round of charge fitting calculation. This process was iterated until the corrected reaction field energy calculated with Delphi converged and the variations of point charges were smaller than a certain criterion. Usually, the criterion will be reached within five iterations.

(e) AF-QM/MM-based structural modeling of PR

The general procedure of our AF-QM/MM approach for structural modeling of PR is presented graphically as flowchart in Fig. S17. Briefly, we have adjusted the protein and retinal structures in iterations for reaching the consistency between the AF-QM/MM calculated and the experimental chemical shifts as described below. Each initial step in our protocol focuses on a specific region of the protein. The structural templates were generated by homology modeling based on the BPR X-ray crystallography structure (PDB 4JQ6) (*17*) as described previously (*16*). As already described in S5.1b, in this work we have focused on refining the structure of retinal chromophore and its binding pocket. The modeling of retinal chromophore structure at the atomic level is more demanding than for proteins. In addition, retinal chemical shifts are rather sensitive to the protein surrounding. Therefore, our modeling has started from the protein part for resolving first the major protein structural changes between GPR and BPR. Once this has been accomplished satisfactorily, we have then moved onto an iterative modeling procedure covering both the fine structural adjustment of protein and retinal chromophore. We have also taken the advantage of the high efficiency of AF-QM/MM to map the impacts of the structural adjustment in the specifically focused region in each step on the other regions. This has permitted us to monitor the potential coupling between different key regions in PRs. In our structural modeling procedure, additional experimental NMR data and

supporting MD simulations have also been supplied when necessary. The detailed procedure for our structural modeling of PRs is described in details below:

First, we have resolved the key structural ambiguity in the area near color switching residue Leu/Gln105. The BPR homologue structure determined by Ran et al. (PDB 4JQ6) (*17*) shows diverse local structures in the region involving Gln105 in different protomers. We have taken these distinct conformations, including the water molecules close to residue 105, as the structural candidates and have checked their local chemical shifts by AF-QM/MM focusing on the nearby residues Trp197 and Asn230. We have also introduced one additional structural candidate using bacteriorhodopsin (PDB 1C3W (*31*)) as the template. Only the combination of GPR structure similar to 1C3W and BPR structure similar to chain A of 4JQ6 provided a simulated chemical shift difference of Trp 197 Nε1 (5.0 ppm) that could qualitatively reproduce our experimental observations (6.3 ppm, see Fig. 3d). The other combinations have led to chemical shift differences in the range from -5.7 to -0.3 ppm and therefore can be excluded. In addition, the conformations selected by our simulations also reproduce correctly the ^{15}Nδ2 chemical shift differences of Asn230 sidechain, which further validates the assigned local conformations. The trend of chemical shift changes captured by DFT calculations has persisted and has even been further optimized in the later stage of modeling.

Second, we have refined the interhelical Trp159-Ile193 contact based on the data obtained in Text S11e. The antiparallel trend of the chemical shift perturbation of Trp159 Nε1 and Ile193 C' suggest a change in the H-bond between these two sites. We have first performed geometry optimization on these residues in GPR and BPR models refined in the above step by keeping the other residues fixed. After geometry optimization, we have scanned the position of the Trp159 indole ring by varying the Cα-Cβ-Cγ-Cδ2 dihedral angle and have matched the simulated NMR chemical shifts with experimental values. After the initial modeling, the H-O distance in GPR and BPR differs by about 0.03 Å (1.95 Å in GPR, 1.92 Å in BPR), which agrees with the empirical trend illustrated by a previous work.(*106*) The simulated chemical shift differences (2.2 ppm for Trp159 Nε1 and -1.1 ppm for Ile193 C') are also in good agreement with the experimental results (1.9 ppm for Trp159 Nε1 and -1.5 ppm for Ile193 C').

Third, we have further optimized other residues in retinal binding pocket (Tab. S6) that show substantial chemical shift differences between GPR and BPR.

Fourth, we have then moved on to the iterative modeling of retinal-protein structures. The previous three steps have already set a proper starting point for the modeling of the fine structures of retinal binding pocket and the sub-atomic resolution structure of retinal in this step. Importantly, all the above modeling steps have not shown strong perturbation (> 5 ppm) on retinal ^{13}C chemical shifts. This excludes strong steric clashing (therefore orbital overlapping) between retinal and protein environment. We have fixed the counter ion to be same in both PRs and have also included in this stage the specific structural information on retinal from our previous publication.(*15*) The retinal structure has been refined together with pSB and Lys231 sidechain by including all these moieties into one large QM core region (Fig. S16, Tab. S5). The subatomic resolution structural modeling (with bond length variation at the scale of 0.01 Å has been supported by the available ^{13}C CSA values, which are highly sensitive to the C-C bond length and therefore show very high discriminative power in fine structural differences. Here the ^{13}C CSA also offers us a "3D view" of the retinal structure and electronic structure at the chemical resolution that cannot be accessed by other methods. The key regions refined in three above initial steps have also been subjected to further structural modeling in this step. In this step, all the protein sites, including the key regions refined in three above initial steps, have been also explicitly simulated by the AF-QM/MM method and been subjected to the structural modeling. The joint structural modeling of both retinal chromophore

and the protein environment has been concluded once no further improvement can be achieved and all the deviations of simulated values from experimental values have reached the expected thresholds. The comparison of experimental and DFT simulated chemical shift parameters can be found in Tab S6-S7. The detailed analysis of our structural modeling results can be found in S5.

Text S13: Analysis of PR structural modeling results

Our PR structural modeling have reached excellent consistency between experimental and QM/MM calculated chemical shift and CSA values. The BPR/GPR ^{13}C and ^{15}N chemical shift differences calculated from our final structural models match much better the experimental values than those obtained from our starting structures (S18e-i). After modeling, the mean standard errors (MSEs) of ^{13}C and ^{15}N chemical shift differences are 0.6 and 0.3 ppm respectively (Tab. S9), which already fall into the inherent accuracy of the AF-QM/MM method (*38, 50, 61, 99*). These two values have been drastically optimized than our starting condition (0.9 ppm for MSE of ^{13}C chemical shift differences and 3.0 ppm for MSE of ^{15}N chemical shift differences, Tab. S10). Our NMR-guided structural modeling has also yielded better linearity of experiment vs. DFT comparison as shown by the drastically improved slope of these correlations (Tabs. S9-S10). The detailed analysis of the AF-QM/MM calculated chemical shift parameters with and without structural modeling are summarized in Tab. S8-S9. The relatively lower correlation between experimental and simulated protein $\Delta\delta(^{13}C)$ are biased by the small range of data, in particular for chemical shift differences rather than the absolute chemical shifts. Here the MSE should be a better parameter for evaluating the quality of our structural modeling than the slope of the correlation.

On retinal chromophore, the correlation between experimental and simulated CSA principal values and isotropic ^{1}H chemical shifts of retinal chromophore present highly similar slopes (about 0.9, Fig. S181-d, Tab. S9), indicating that our AF-QM/MM treatment of these parameters are self-consistent. As shown in Fig. S18a-c, our structural modeling has significantly reduced MSE of retinal carbon CSA principal values from 8.9 ppm (8.2 for BPR and 10.2 ppm for GPR, Tab. S10) of starting structures to 5.7 ppm (5.3 ppm for BPR and 6.4 ppm for GPR, Tab. S9) of optimized structures.

Our PR structural models are supported by a number of independent and heterogeneous criteria. First, our structural models, which have been refined without the chemical shift information of pSB hydrogen, reproduce the chemical shift difference of this atom between two PR constructs (0.6 ppm as calculated vs. 0.8 ppm as experimentally determined). Therefore, our models capture faithfully the chemical features of this key site. Second, the modeled local structure close to the color-switching residues, including the local water molecule, is consistent with our MD simulations using polarized force field. Third, our structural models reproduce the experimental retinal light absorption wavelength (Text S16, S17). In addition, the refined protein structures even reproduce the light absorption behaviors of PRs with retinal substituted by 3,4-deH$_2$ retinal (Tab. S13).

We have further compared our BPR and GPR structural models with available PR structures determined by X-ray crystallography (PDB 4JQ6, 4KLY)(*17*) or cryo-EM (PDB 7B03)(*18*) as well as structural models predicted by AlphaFold2(*40*). Here we have focused on the retinal binding pocket residues. As shown in Tab. 11, the retinal binding pocket of these structural models different from each other at similar levels as shown by RMSD values. The RMSD of retinal binding pocket between our GPR and BPR structural models is 0.4 Å. Interestingly, the RMSD of retinal binding pocket between different protomers in X-ray crystallographic structures of BPRs are at the similar level (0.2 – 0.5 Å). The RMSD of retinal binding pocket between our GPR structural model and the corresponding cryo-EM structure

(7B03) is 0.8 Å. The cryo-EM structure of GPR was obtained at non-physiologically acidic pH. As a reference, the RMSD of retinal binding pocket between cryo-EM structure of GPR and X-ray crystallography structures of BPR is in the range of 0.7 – 0.8 Å. These analyses show that our structural modeling has captured small structural changes in the retinal binding pocket.

Text S14: MD simulations of PRs

The MD simulations were performed using AMBER 16 with GPU acceleration (pmemd.cuda)(*62*). The AF-QM/MM models generated in Text S12e were used as the starting input structures for MD simulations. The simulation was performed at a constant temperature of 300 K in the NPT ensemble, using Langevin dynamics with a friction coefficient of 1 ps^{-1}. The constant pressure is 1 bar and is controlled by Berendsen pressure scaling algorithm with a relaxation time of 8 ps. The SHAKE algorithm was used to constrain the hydrogen atoms. The particle-mesh Ewald method was applied for electrostatics calculations with nonbonded cutoff of 8.0 Å. Each simulation was integrated at a time step of 2 fs for a total MD trajectory of 10 ns. The MD simulations were performed on an in-house GPU cluster (GeForce GTX 2080 Ti).

We applied two different charge models for the electrostatic embedding field, namely, the Amber 94 charge model and the polarized protein-specific charge (PPC) model in our calculations. Our previous studies have found that MD simulations using the standard non-polarizable Amber force field may break some of the backbone hydrogen bonds in native secondary structures, which results in structural deformation due to the lack of electronic polarization effects (*50*). Therefore, in this study we also employed the recently developed polarized protein-specific charge (PPC) model (Text S15) to provide a new set of atomic charges for a better description of the protein environment (*49*). This change model is also implemented in our AF-QM/MM simulations (Text S12d), which ensures the consistency between the AF-QM/MM and MD simulations in this work.

Text S15: Derivation of protein electrostatic potential and electric field strength

The electrostatic field (E-field) within the protein can be computed by exploring protein PPC changes (Text S14) following the equation below:

$$\left|E_{C5-\mathrm{N}}\right| = \frac{\sum_{\substack{i=1 \\ i \notin Ret|K231}}^{N} \frac{1}{4\pi\varepsilon}\left(\frac{q_i}{\left|\vec{r}_i - \vec{r}_{C5}\right|} - \frac{q_i}{\left|\vec{r}_i - \vec{r}_{\mathrm{N}}\right|}\right)}{\left|\vec{r}_{\mathrm{C5}} - \vec{r}_{\mathrm{N}}\right|}$$

where $|E_{\mathrm{C5-N}}|$ is the magnitude of the average electrostatic field induced by the protein residues (excluding the retinal itself and Lys231) from C5 on the polyene chain of the retinal to pSB nitrogen, which approximates the electric field along the polyene chain. $i \notin \mathrm{Ret|K231}$ denotes that the atoms of retinal itself and residue Lys231 were excluded in the electric field calculation, q_i is the atomic charge of the *i*th atom, $\vec{r}_{\mathrm{i}}$ represents the coordinate vector of the *i*th atom, *N* represents the number of atoms in the protein, $\left|\vec{r}_i - \vec{r}_{\mathrm{C5}}\right|$ denotes the distance between protein atom *i* and retinal C5, and $\left|\vec{r}_i - \vec{r}_N\right|$ denotes the distance between protein atom *i* and pSB nitrogen.

Text S16: PR light absorption wavelength by TD-DFT simulations

The excited state calculations of the rhodopsins were performed using the QM/MM method at the TD-B3LYP/6-311+G* level (*41-43*). The QM region contains the whole retinal,

the pSB and the linked Cε atom (on Lys231). The dangling bond was capped by a hydrogen atom(*41*). The other residues along with the bridging water in the pocket were treated as background charges. We applied polarized protein-specific charge (PPC) model for the electrostatic embedding field in our calculations. The molecular orbital files were exported from GaussView.

Text S17: Analysis of contributors to PR color switching

The contribution of various structural, chemical and physical factors in the color tuning effect has been investigated in details by performing the *in-silico* simulations based on our PR structural models. Briefly, starting from the refined retinal structure, we have gradually added protein environment, specific water molecules in the color switching center and protein E-field to interrogate the impacts of these factors in the color switching (Fig. 4). For each case we have computed the S0-S1 energy gap using the TD-DFT method as described in Text S16. The effect of protein E-field has been examined by including or excluding the PPC charges in the simulations. It is worth noting that such a "deconvolution" cannot be performed experimentally via routine mutagenesis/chemical biology approaches as all these factors are coupled to each other and their *causal effects* on color switching can only be separated using the *in-silico* approach.

UV-Vis spectra of microbial rhodopsins, mutants and retinal bleaching experiments

Text S18: Expression and purification of diverse microbial rhodopsins

A scheme of microbial rhodopsins, including Exiguobacterium sibiricum (ESR, proton pump), Nonlabens marinus rhodopsin 2 (NM-R2, sodium pump), Nonlabens marinus rhodopsin 3 (NM-R3, chloride pump) and Natronomonas pharaonis rhodopsin (NpHR, chloride pump) have been cloned to pET plasmids and expressed recombinantly in *E. coli* C43 strain. The respect genes were, if stated, codon optimized for *E. coli* and synthesized by Genscript (www.genscript.com). The detailed information about the construct design and molecular cloning are summarized in Tab. S14. All these proteins have been expressed in M9 minimal medium. The protein expression was induced at OD_{600} 0.4. The membrane fractions were prepared as described in Text S2. All these proteins were solubilized in DDM except for NM_R2, which requires Triton X-100 for the effective solubilization. The proteins have been purified using IMC (Immobilized Metal ion affinity Chromatography) on NiNTA column with imidazole as the elution agent. The detailed conditions for the protein expression and purification are summarized in Tab. S15. For all downstream characterizations, such as pH titration and UV-Vis spectroscopy, the excess imidazole was removed via PD-10 column.

Text S19: UV-Vis spectroscopic characterization of diverse microbial rhodopsins

UV-Vis light absorption spectra of retinal proteins were recorded on a Jasco V550 spectrometer. Since the retinal absorption wavelength of the counter-ion deprotonated form is relevant to our study, pH titration experiments were first carried out. For this, the purified proteins were first buffer exchanged with Britton-Robinson buffer (0.04 M boric acid, 0.04 M phosphoric acid, 0.04 M acetic acid, titrated to pH 7 with 0.2 M NaOH, 0.05% DDM) on PD-10 column (GE Life Science) using the standard protocol as advertised from the manufacturer. The pH value of protein solution was gradually tuned by adding NaOH or HCl solution (1 M). For each pH, a UV-Vis spectrum was recorded. Such pH titration allowed us to decide the proper alkaline pH (plateau) for recoding the UV-Vis light absorption spectra of respective proteins. Since NM_R3 binds Cl^- ion in its dark state, 4 M NaCl was included in the experiments. All UV-Vis spectra are shown in Fig. S21.

Text S20: Retinal bleaching experiments

The covalently bonded retinal chromophore in PRs can be removed chemically by NH_2OH via the substitution of Lys231 sidechain by this small molecule. The kinetics of NH_2OH chemical bleaching is most limited by the accessibility of pSB site (reaction site) by NH_2OH. Since the retinal cleaved and released from protein loses the unique opsin-shift, the kinetics of this reaction can be followed by monitoring the PR retinal absorption by real-time UV-Vis spectroscopy. The purified protein was buffer exchanged on PD 10 column to an imidazole-free buffer (300 mM NaCl, 50 mM Tris, 0.05% DDM, pH 8). The protein concentration was adjusted so that the light absorption at the bound-retinal wavelength was about 0.9 (1.0 cm path, about 17.5 μM). The NH_2OH stock (50% w/v, ca. 15 M, 1 μL) was added directly into the PR solution (500 μl) in dark, which set the initial concentration (ca. 30 mM) to be much higher than the protein concentration and therefore maintained a constant NH_2OH concentration during the measurement. The dead time before the addition of NH_2OH solution and the first point of UV-Vis readout was about 20 s. The absorption at the bound retinal wavelength was recorded every 5 s.

Bioinformatics

Text S21: Bioinformatics analysis of PR color switching

PR sequences have been retrieved from four resources, namely MicRhoDE (*63*), Tara Oceans expedition (*64*) and two recent metagenomics reports from the DeLong lab (*7*) and the Beja group (*23*). MicRhoDE (http://application.sb-roscoff.fr/micrhode/doblast) is a curated database for microbial rhodopsin sequences collected from diverse geographic locations up to 2015. The non-PR sequences in MicRhoDE have been removed via the assigned annotation in the original database. PR sequences obtained from the Tara Oceans expedition (2009 - 2013) have been fetched from Ocean Gene Atlas (https://tara-oceans.mio.osupytheas.fr/ocean-gene-atlas/) through the summary by Martori Lopez and coworkers (*107*). The PR sequences collected in the North Pacific Subtropical Gyre (ALOHA station, 2010 - 2011) have been obtained via the gene catalog created by DeLong and coworkers (*7*). The red sea PR sequences obtained from the functional screening by Beja and coworkers (*23*) have been retrieved manually from NCBI database. We have removed those sequences shorter than 200 residues in order to ensure a good reading coverage of the sequenced for our analysis, which yields 547, 2616, 905 and 11 long-reading PR sequences from MicRhoDE, Tara Oceans, ALOHA and red-sea screening respectively.

The MicRhoDE sequences were already aligned while retrieved. PR sequences from other resourced have been aligned to GPR reference sequence (γ-proteobacterium EBAC31A08) by ClusterOmega. All the pre-processing of the sequences and the statistical analysis of the residue type at position 105 have been carried out by home-written python scripts. The aligned sequences have been classified into GPR and BPR groups featuring Leu and Gln at position 105. The degrees of conservation at each residue site across two PR clades have been calculated by in-house python scripts. The inter-clade comparison, which is shown in Fig. 1d, has been generated based on the statistics from the previous step. In total, 686 GPR and 2154 BPR sequences have been analyzed.

The MicRhoDE sequences were already aligned while retrieved. PR sequences from other resources have been aligned to a GPR reference sequence (γ-proteobacterium EBAC31A08) by ClusterOmega. All the pre-processing of the sequences and the statistical analysis of the residue type at position 105 have been carried out by home-written python

scripts. The aligned sequences have been classified into GPR and BPR groups featuring Leu and Gln at position 105. The degrees of conservation at each residue site across both PR groups have been calculated by in-house python scripts. The BPR-GPR comparison, which is shown in Fig. 1d, has been generated based on the statistics from the previous step. In total, 686 GPR and 2154 BPR sequences have been analyzed.

For the evaluation of residue coevolution patterns and conservation levels, the above curated BPR and GPR sequences were further analyzed by ConSurf (*26, 27, 65*) and MISTIC2 (*28*) to take the uneven sequence similarity and phylogenetic structures into account. For computing the conservation scores, the multi-sequence alignment (MSA) of curated GPR, BPR (Fig. 22) and combined GPR and BPR sequences (for Fig. 4i) were submitted to the ConSurf server. The MISTIC2 analysis were performed for the combined GPR and BPR sequences using standard parameters (Fig. 23). The Bayesian method with WAG evolutionary substitution model were used for calculating the evolutionary conservation score. The WAG model was chosen by testing multiple models on the ConSurf server.

Supporting Figures

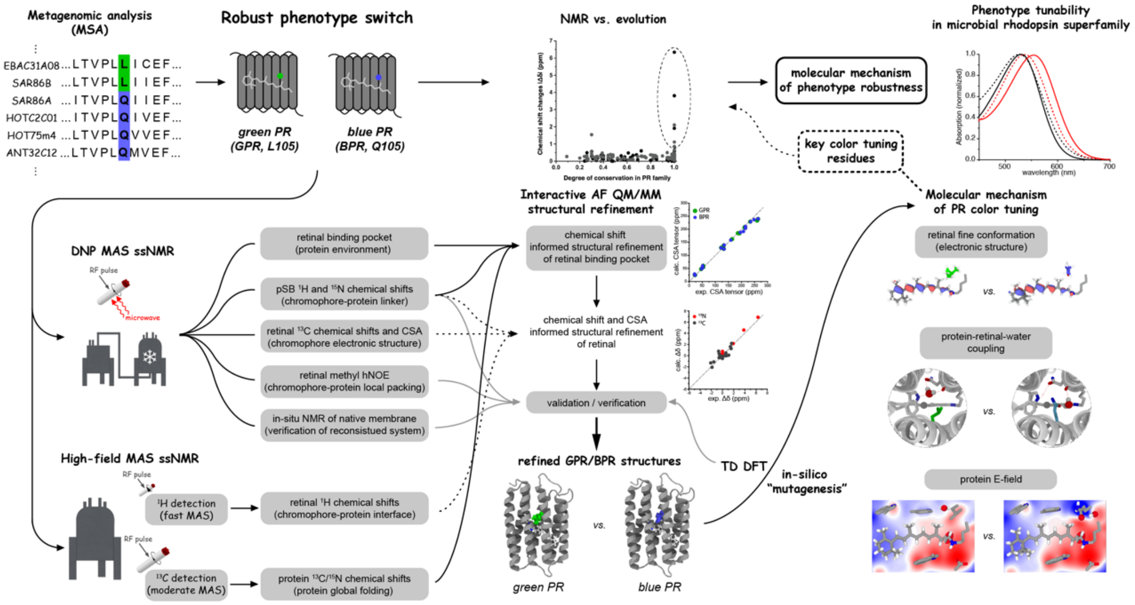

Fig. S1: Schematic overview of the integrative approaches used in this work.

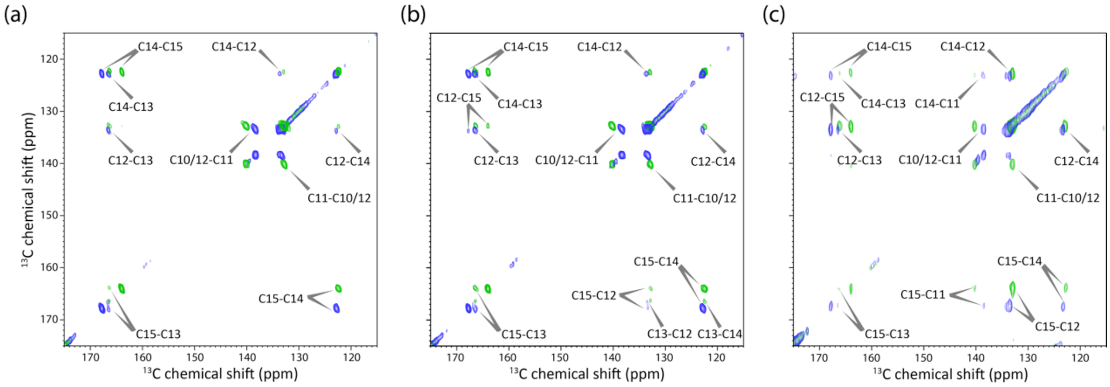

Fig. S2: ^{13}C-^{13}C 2D proton-driven spin-diffusion (PDSD) spectra of [$^{13}C_{10\text{-}18}$-ret, U-^{15}N]-GPR (green) and [$^{13}C_{10\text{-}18}$-ret, U-^{15}N]-BPR (blue). The chemical assignments of peaks are listed in the format of F1-F2. All spectra were acquired at room-temperature on a Bruker 850 MHz ssNMR spectrometer. MAS frequency was stabilized at 14 kHz. The ^{13}C-^{13}C mixing time was set to 10 ms **(a)**, 20 ms **(b)** and 800 ms **(c)**, respectively. Chemical shifts are summarized in Tab. S2 and chemical shift differences are plotted in Fig. 2e.

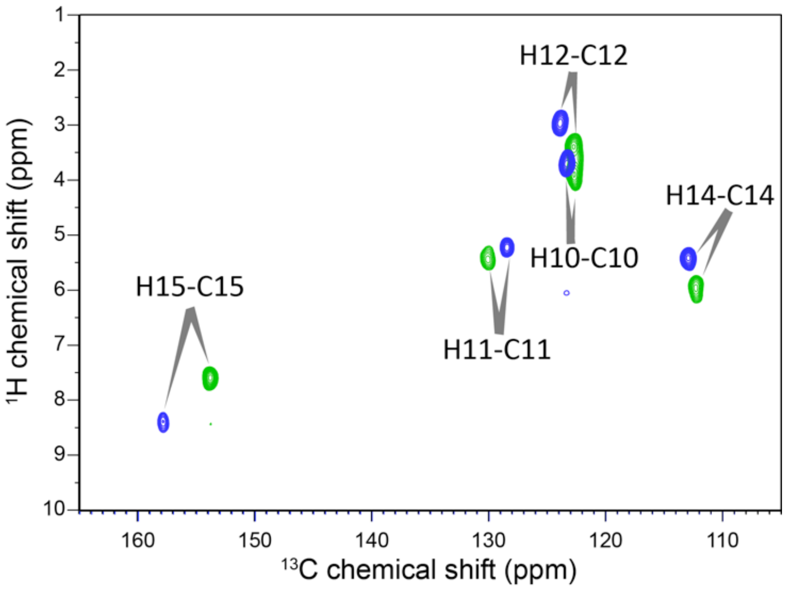

Fig. S3: ^{1}H-^{13}C 2D heteronuclear correlation (HETCOR) spectra of [$^{13}C_{10\text{-}18}$-ret, U-^{15}N]-GPR (green) and [$^{13}C_{10\text{-}18}$-ret, U-^{15}N]-BPR (blue). The chemical assignments of peaks are listed in the format of F1-F2. All spectra were acquired at room-temperature on a Bruker 850 MHz ssNMR spectrometer. MAS was stabilized at 14 kHz. Initial magnetization was created by a short ^{1}H-^{13}C CP step of 100 μs and homonuclear FSLG decoupling at 90 kHz was applied during *t1* evolution. Chemical shifts and chemical shift differences are summarized in Tab. S3.

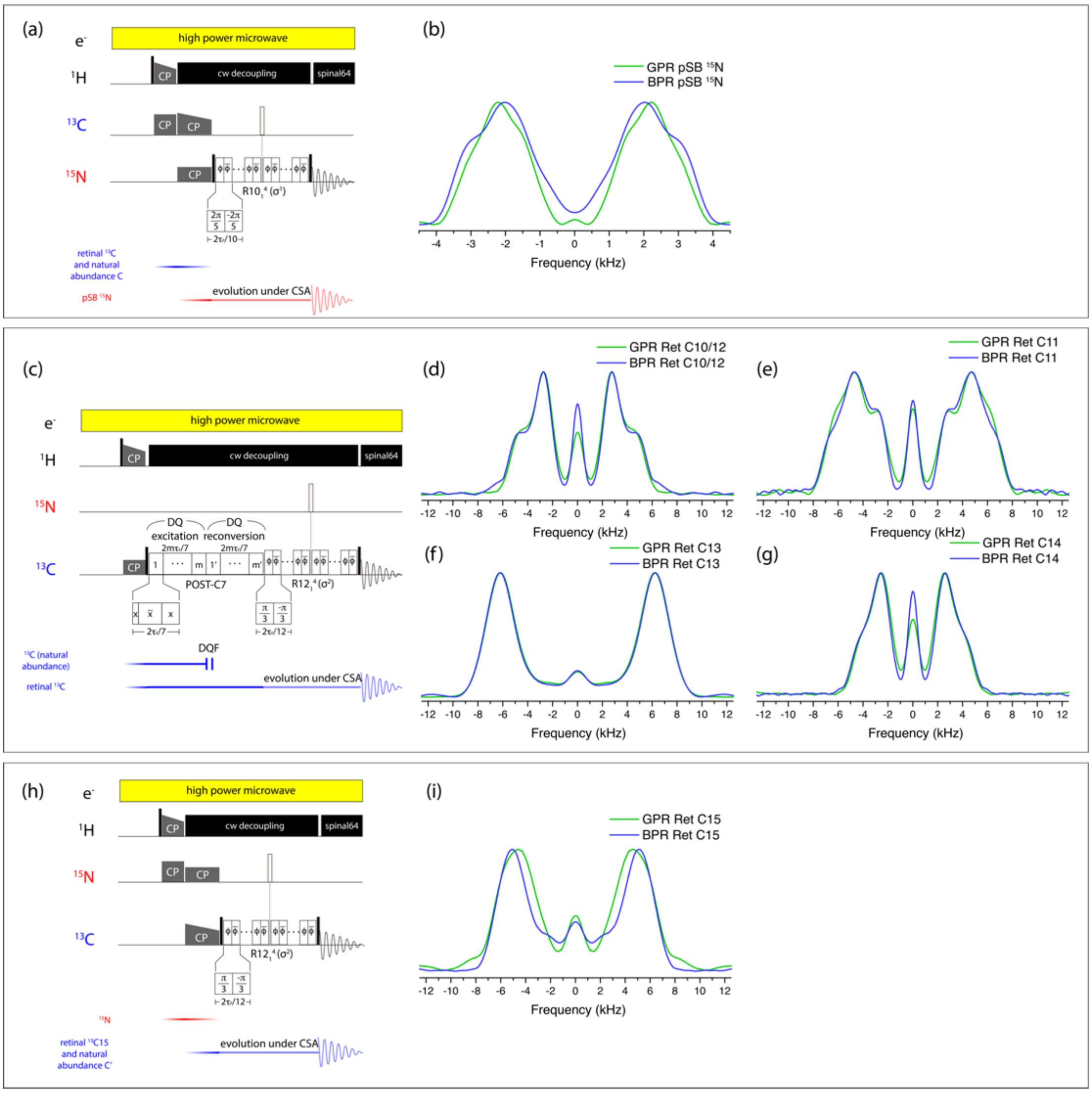

Fig. S4: DNP-enhanced ^{15}N and ^{13}C RNCSA recoupling experiments on retinal carbons and pSB nitrogen in GPR and BPR. **(a)** R-symmetry based recoupling scheme $R10_1^4$ (σ_1) pulse sequence for ^{15}N CSA recoupling used on samples of [$^{13}C_{10\text{-}18}$-ret, U-^{15}N]-PRs. The ^{13}C-^{15}N DCP step is required to select the Schiff base nitrogen signal via the C15 retinal carbon. **(b)** Recoupled CSA pattern of the Schiff base nitrogen in PRs. **(c)** R-symmetry based recoupling scheme $R12_1^4$ (σ_2) pulse sequence for ^{13}C CSA recoupling used on samples of [$^{13}C_{10\text{-}18}$-ret, U-^{15}N]- and [$^{13}C_{12,13,20}$-ret, U-^{15}N]-PRs. The POST-C7 double quantum filtering step is required to suppress ^{13}C natural abundance protein background. **(d)** CSA pattern of retinal carbons C10/C12. The spectra overlap due to the almost identical chemical shifts of C10 and C12. **(e)** CSA pattern of retinal carbons C11. **(f)** CSA pattern of retinal carbons C13. Here, the samples [$^{13}C_{12,13,20}$-ret, U-^{15}N]-PRs were used to resolve the C13 spectrum. **(g)** CSA pattern of retinal carbons C14. **(h)** To resolve C15, which partially overlaps with C13 in samples [$^{13}C_{10\text{-}18}$-ret, U-^{15}N]-PRs, a ^{15}N-^{13}C DCP step was applied prior the $R12_1^4$ pulse train. In this way, C15 was selected via the directly bonded Schiff base nitrogen. **(i)** CSA pattern of retinal carbons C15.

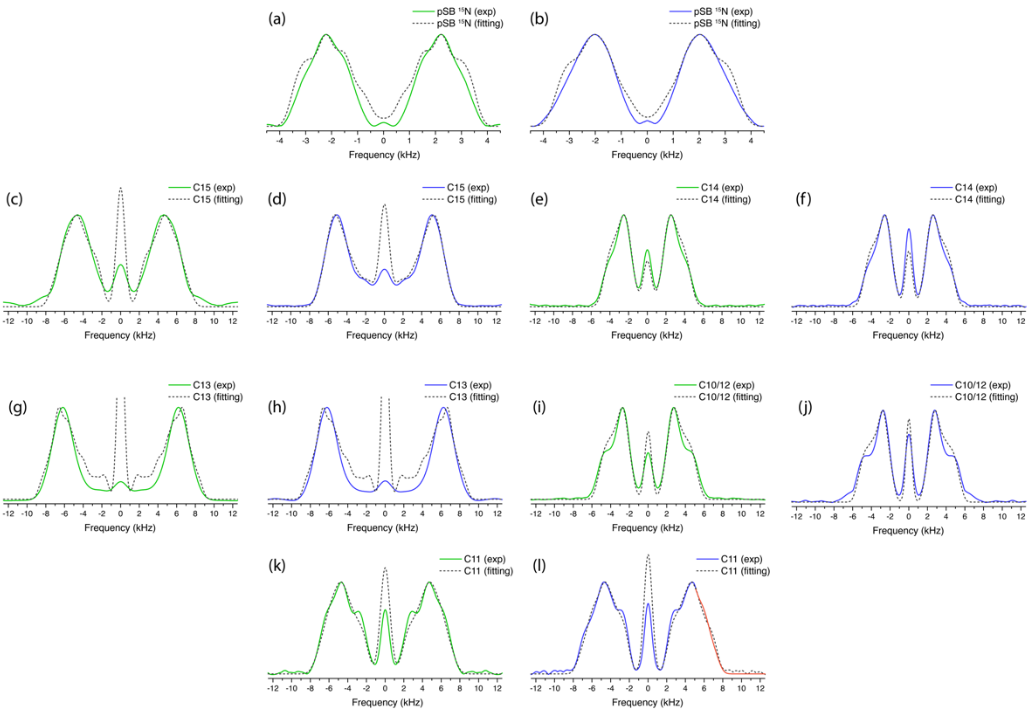

Fig. S5: Fitting of recoupled CSA patterns obtained from RNCSA experiments (Fig. S4). The SIMPSON-simulated best-matching CSA patterns are shown in dashed lines. The CSA patterns obtained on GPR and BPR are shown in green and blue respectively. The zero frequency signals at the central position of the recoupled patterns were excluded from the simulations. **(a-b)** ^{15}N CSA pattern of the Schiff base nitrogen. **(c-l)** ^{13}C CSA pattern of retinal carbons C10-C15. The CSA tensor values are summarized in Tab. S2.

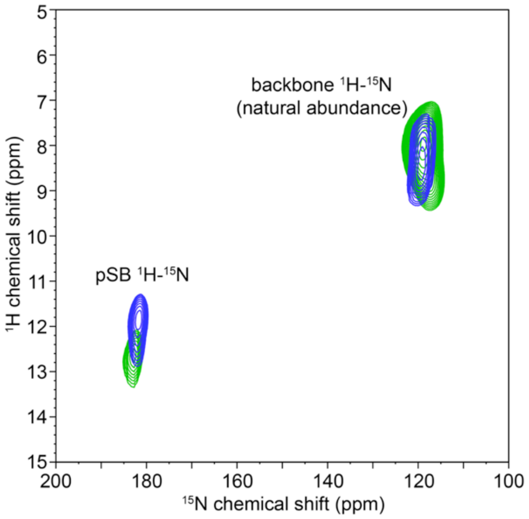

Fig. S6: DNP-enhanced ^{1}H-^{15}N 2D HETCOR spectra of [U-^{13}C, $^{15}N\varepsilon$-Lys]-GPR (green) and – BPR (blue). The spectra were acquired under DNP conditions (110 K). No solvent or protein deuteration was used. High-power ^{1}H FSLG homonuclear decoupling (100 kHz) was applied during the ^{1}H chemical shift evolution period. The theoretical chemical shift FSLG scaling factor (0.578) was used for the F1 (^{1}H) dimension.

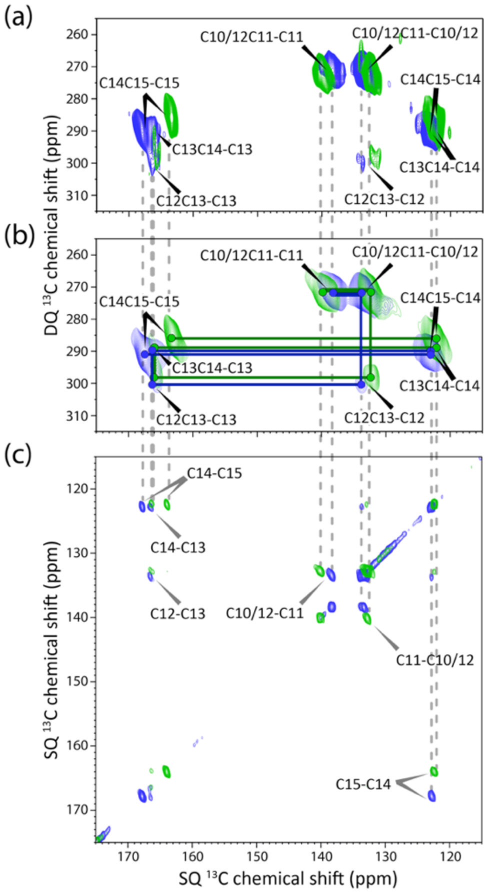

Fig. S7: DNP-enhanced ^{13}C-^{13}C 2D DQ-SQ spectra **(a-b)** and high-field room-temperature 2D ^{13}C-^{13}C PDSD spectra **(c)** of [$^{13}C_{10\text{-}18}$-ret, U-^{15}N]-GPR (green) and -BPR (blue). The DQ-SQ spectra were acquired on purified and reconstituted proteoliposome samples **(b)** or on native cellular membrane **(a)** at 110 K. The DQ excitation and reconversion times were optimized for strong ^{13}C-^{13}C dipolar coupling (directly bonded ^{13}C spin pairs). The PDSD spectrum shown in **(c)** is the same as shown in Fig. S2a (mixing time 10 ms). The retinal polyene ^{13}C chemical shifts are consistent between low temperature and room-temperature spectra as well as between the purified proteins and the native preparation. These observations support that purified and reconstituted PRs are folded correctly and represent the structures in a more native preparation.

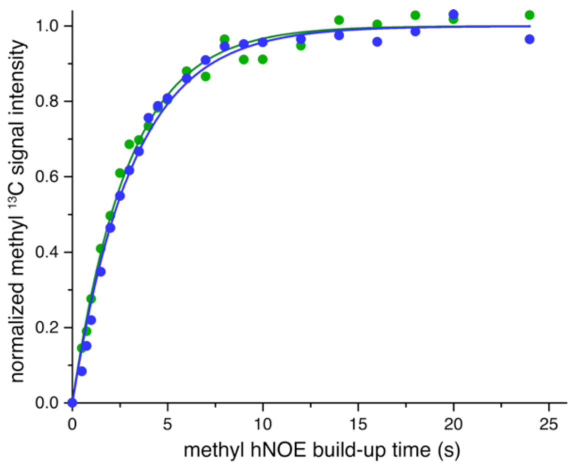

Fig. S8: ^{1}H-^{13}C hetNOE build-up kinetics of the retinal C20 methyl group at 110 K measured in [$^{13}C_{12,13,20}$-ret, U-^{15}N]-GPR (green) and -BPR (blue). All the data were fitted with a mono-exponential function. Almost identical hetNOE build-up times of 2.9 ± 0.1 s for GPR vs. 3.2 s ± 0.1 s for BPR were obtained. This observation indicates that the C20 methyl group is unperturbed by the differences between GPR and BPR with residue L105/Q105 in close proximity.

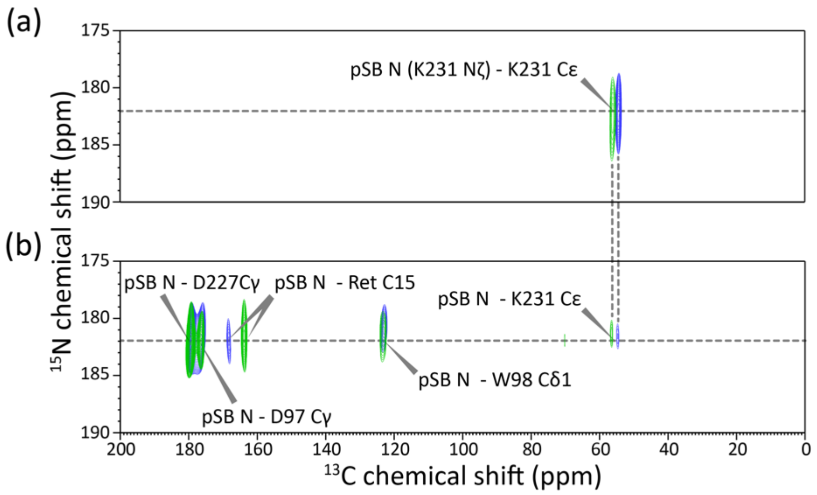

Fig. S9: The counter ion chemical shifts in GPR and BPR. **(a)** ^{15}N-^{13}C DCP spectra of [U-^{13}C, U-^{15}N, rev-His]-G/BPR (green) and -BPR (blue). In contrast to the pSB ^{15}N chemical shift, a clear difference between GPR and BPR is observed for the ^{13}C chemical shift of K231 Cε between GPR and BPR (see also Fig. S10a). **(b)** ^{15}N-^{13}C DCP spectra of [U-^{13}C, ^{15}Nε-Lys]-GPR (green) and -BPR (blue) acquired with a long DCP contact time (25 ms). The Cγ resonances of D97 and D227 were assigned according to previous studies (*15, 86*). They show no chemical shift difference between GPR and BPR. The other cross peaks are caused by natural abundance ^{13}C carbons directly attached to the pSB ^{15}N nitrogen (retinal carbon C15, K231 Cε) or from residue W98 Cδ1 within the retinal binding pocket.

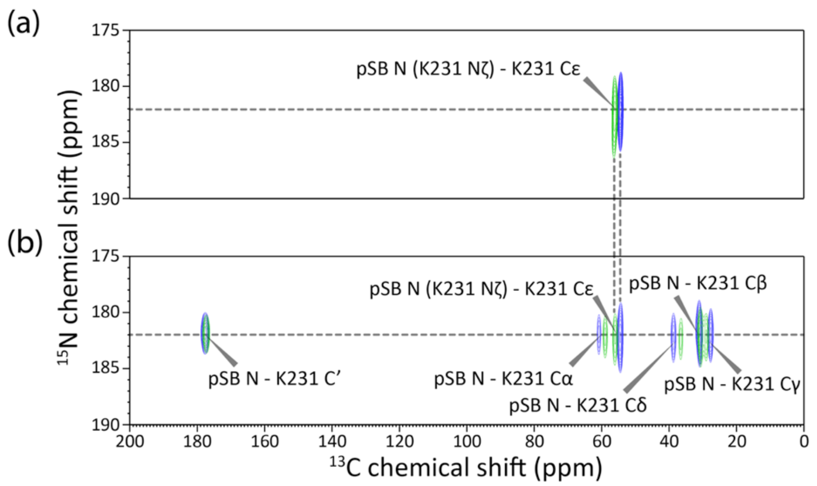

Fig. S10: The K231 sidechain resonances in GPR and BPR. **(a)** ^{15}N-^{13}C DCP spectra of [U-^{13}C, U-^{15}N, rev-His]-GPR (green) and -BPR (blue) (as in Fig. S9b) showing the Nζ-Cε cross peak. **(b)** ^{15}N-^{13}C Nζ (Cε)CX spectra. Chemical shift differences are found in all K321 sidechain carbons and Cα carbon (Tab. S6).

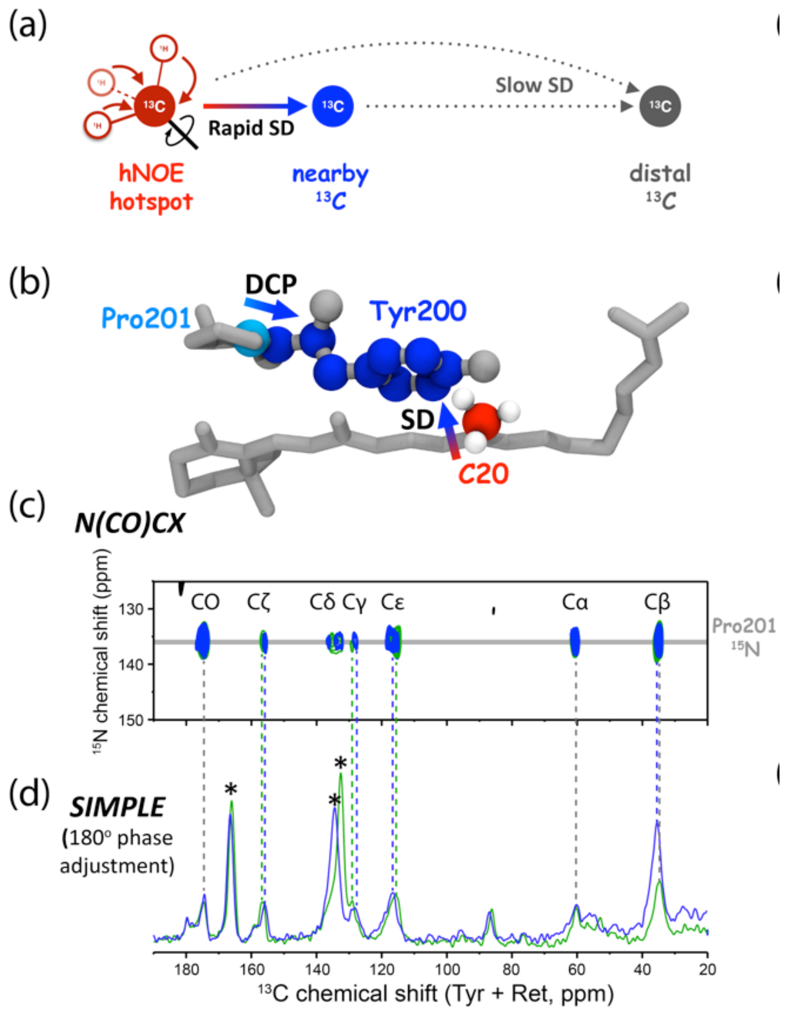

Fig. S11: Selective detection of Tyr200 via SIMPLE DNP NMR trough retinal C20 and by unique pair labelling through Pro201. **(a)** In the SIMPLE approach, the ^{13}C-C20 methyl group is selectively polarized by DNP using hetNOE. A subsequent spin diffusion step transfers magnetization to nearby ^{13}C-labelled sites (*25*). **(b)** In G/BPR, C20 is close to Tyr200. Furthermore, Tyr200 and Pro201 form a unique pair enabling a second possibility for selective detection. (c) 2D ^{15}N-^{13}C N(CO)CX spectra of [U-^{13}C Tyr, ^{15}N-Pro]-G/BPR (green/blue) utilizing the unique pair labelling. **(d)** SIMPLE spectrum of [$^{13}C_{12,13,20}$-ret, U-^{13}C Tyr]-G/BPR with 1.0 s build-up time. The GPR/BPR chemical shift differences are summarized in Tab. S6.

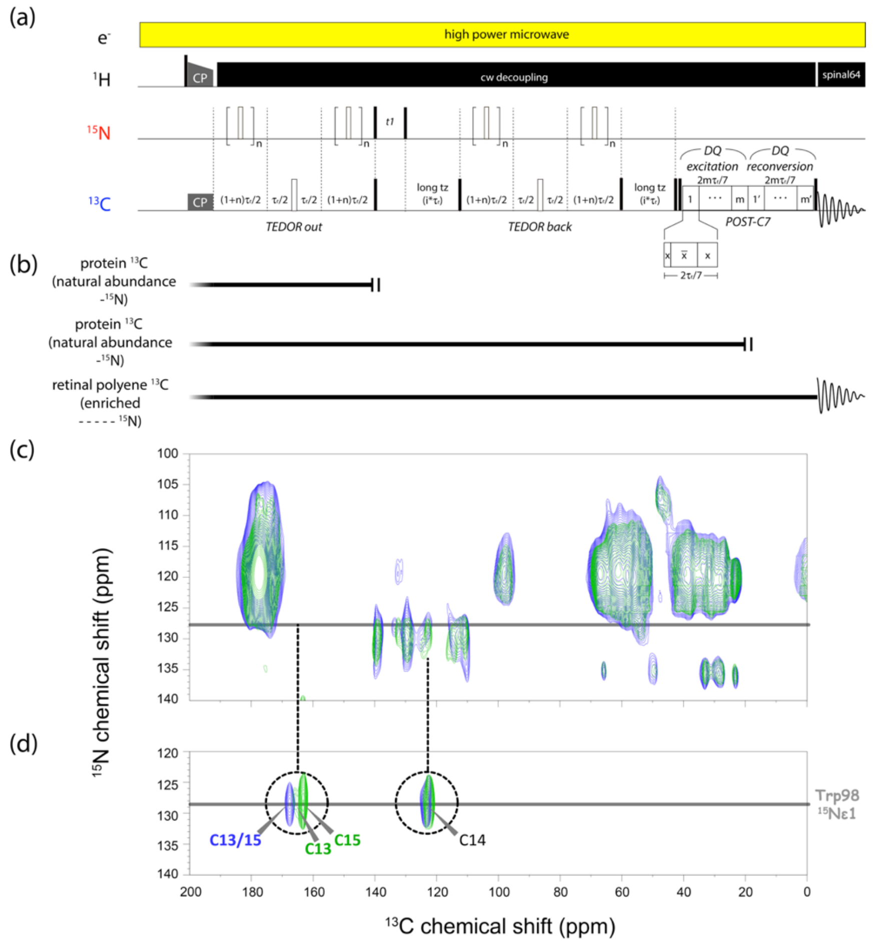

Fig. S12: Detection of the indole nitrogen of Trp98 in [$^{13}C_{10\text{-}18}$-ret, U-^{15}N]-G/BPR. **(a-b)** ^{15}N-^{13}C TEDOR pulse sequence with an additional POST-C7 double quantum filtering step to suppress ^{15}N-^{13}C natural abundance cross peaks. **(c)** Conventional 2D ^{15}N-^{13}C TEDOR spectra of [$^{13}C_{10\text{-}18}$-ret, U-^{15}N]-GPR (green) and -BPR (blue). **(d)** 2D ^{15}N-^{13}C TEDOR spectra with double quantum filter. Here, all natural abundance cross peaks are removed and only correlations between retinal C13, C14 and C15 with Trp98 ^{15}Nε1 are detected.

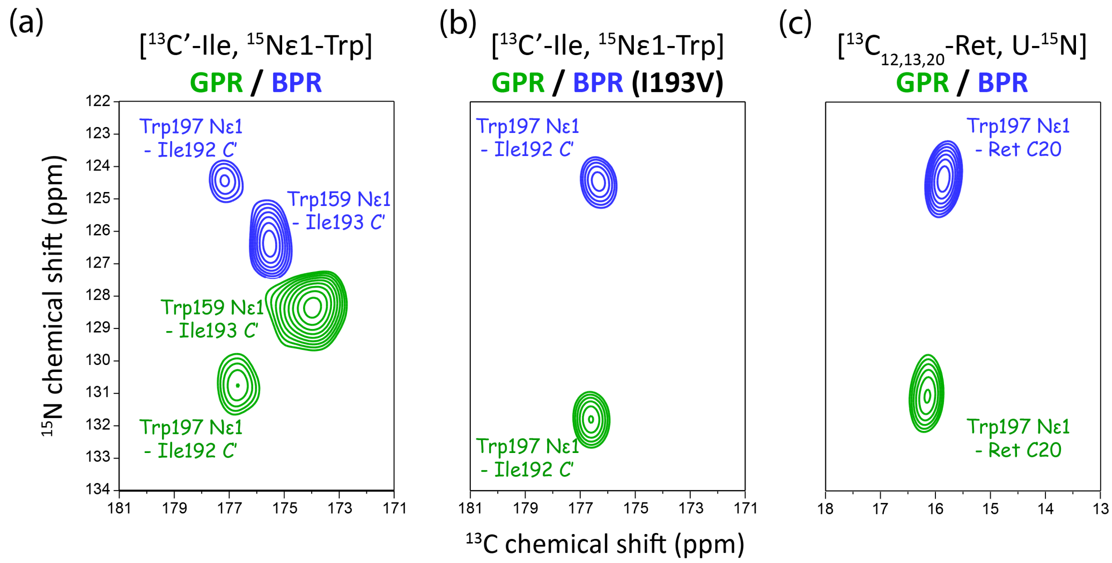

Fig. S13: Detection of Ile193 – Trp159 and Ile192/retinal – Trp197 through-space correlation in PRs. **(a)** ^{15}N-^{13}C TEDOR spectra of [^{13}C'-Ile,^{15}Nε-Trp]-G/BPR (green/blue). **(b)** ^{15}N-^{13}C TEDOR spectra of the I93V mutant of [^{13}C'-Ile,^{15}Nε-Trp]-G/BPR allows assignment of the cross peaks Ile192-Trp197 and Ile193-Trp159 based on the structural information from the X-ray structure 4JQ6(*17*). **(c)** The Trp197 sidechain Nε1 chemical shifts can also be validated via the Trp197-retinal contact visualized by the 2D ^{15}N-^{13}C TEDOR spectra of [^{13}C$_{12,13,20}$-retinal, U-^{15}N]-PRs. Chemical shift changes are summarized in Tab. S6.

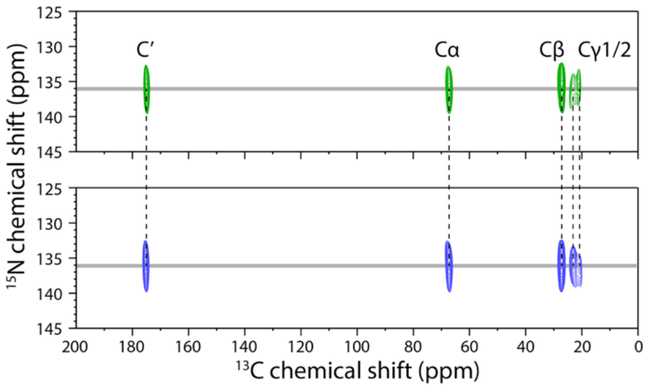

Fig. S14: Visualizing the unique Val102-Pro103 pair in G/BPR. 2D ^{15}N-^{13}C N(CO)CX spectra of [U-^{13}C Tyr, ^{15}N-Pro]-G/BPR (green/blue). No chemical shift changes have been found in these spectra.

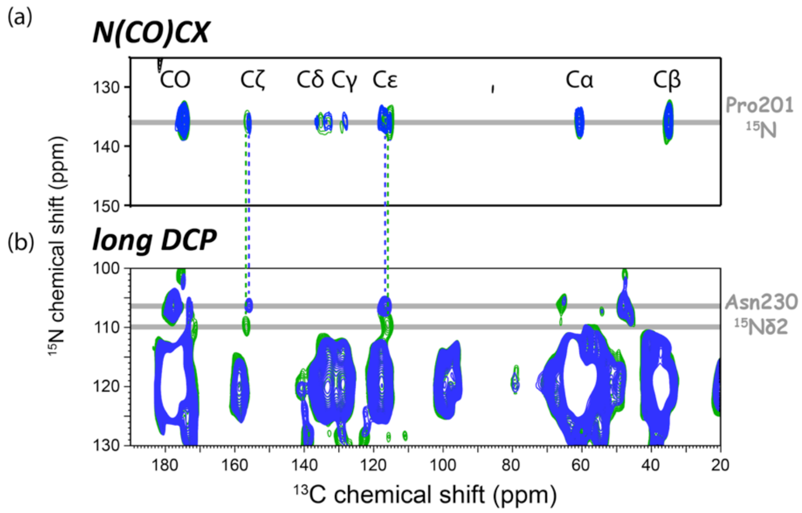

Fig. S15: Detection of Asn230 ^{15}Nδ2 via through-space correlation with ^{13}C-Tyr200. **(a)** ^{15}N-^{13}C DCP spectra of [U-^{13}C Tyr, ^{15}N-Pro]-G/BPR (green/blue). The unique pair Tyr200/Pro201 allows assignment of the Tyr200 ^{13}C resonances (Fig. S11c replotted, see description above). **(b)** ^{15}N-^{13}C DCP spectra of [U-^{13}C Tyr, U-^{15}N]-G/BPR with a long DCP contact time (25 ms) showing Tyr200-Asn230 correlations. The assignment of Asn230 is based in the unique ^{15}N chemical shift of Asn Nδ2 and due to the fact, that it is the only Asn in proximity of Tyr200. Small chemical shift differences between GPR and BPR are detected (Tab. S6).

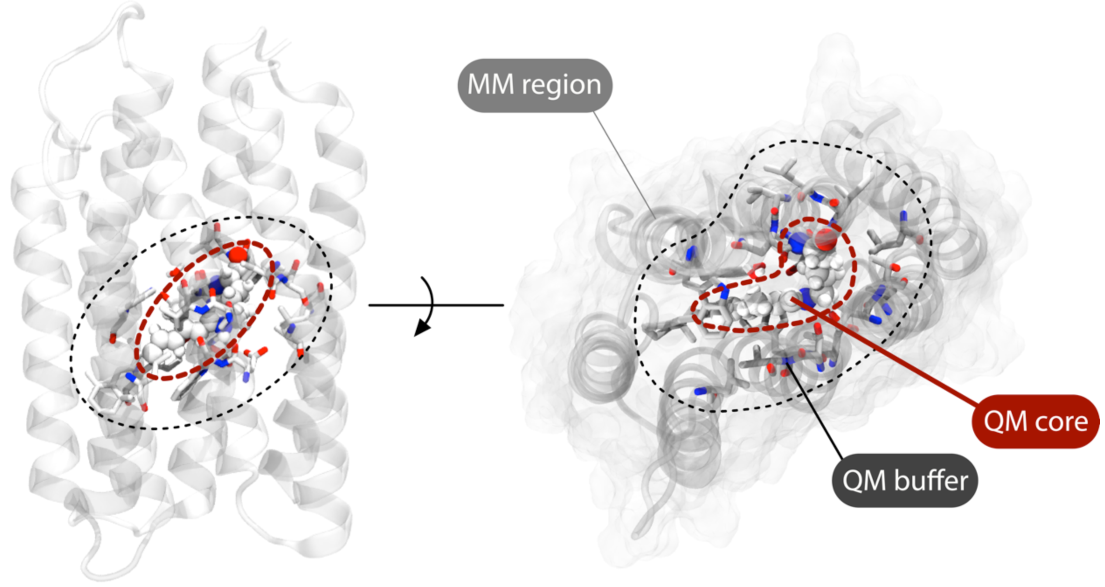

Fig. S16: Demonstration of QM and MM regions in the AF-QM/MM approach. As an example, retinal chromophore together with its covalently bound Lys231 residue was selected as the QM core region. The surrounding residues are selected as the QM buffer region, which was also treated at the QM level but was not subjected to chemical shift calculations. The rest of the system was treated by MM. The presence of QM buffer region "shields" the QM core region from the MM region and therefore prevents the biasing of chemical shift calculation by the mismatching of QM and MM methods at the boundary region. This is the key difference between our AF-QM/MM approach and the conventional QM/MM method. The size of each region can be found in Tab. S5.

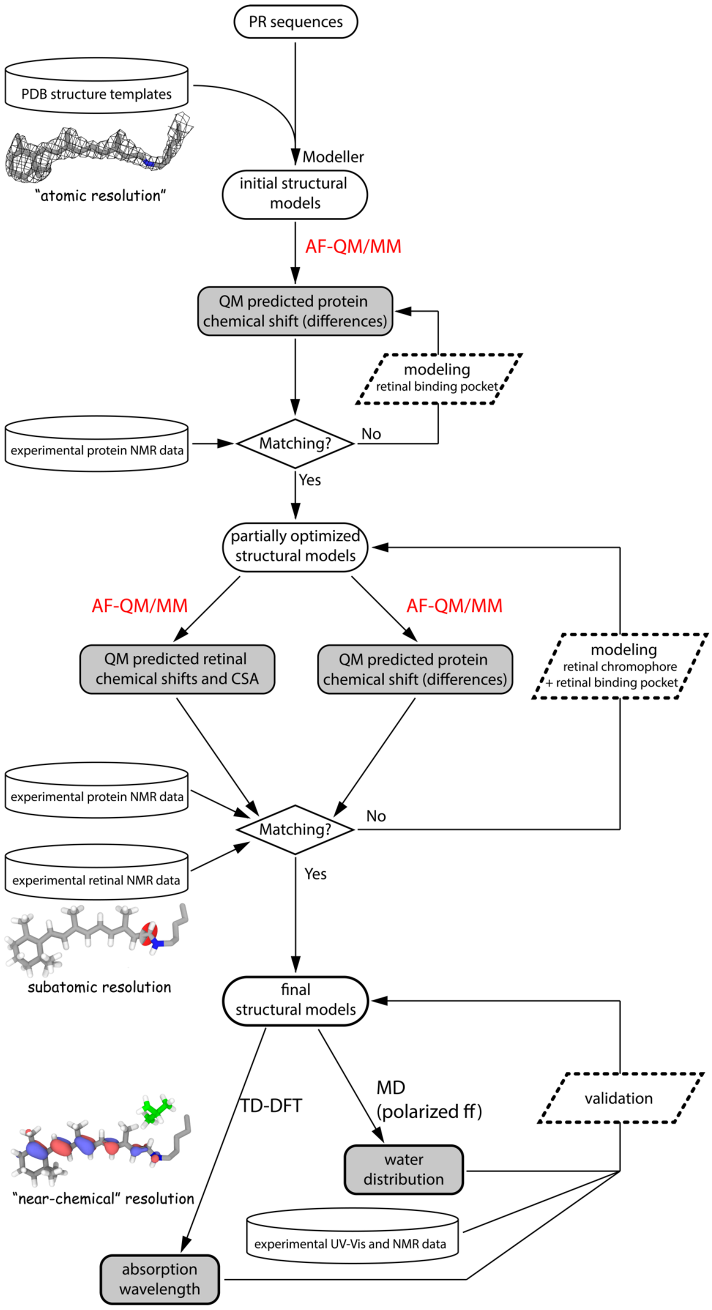

Fig. S17: Flowchart of the AF-QM/MM structural modeling of PRs. The initial structural models of GPR and BPR were built by homology modeling using the BPR structure (4JQ6) as template (near-atomic resolution). These models were submitted to supervised AF-QM/MM structural modeling against the experimental NMR chemical shifts on the protein (binding pocket residues). The refined models were then used for the second round AF-QM/MM structural modeling that targets both the protein chemical shift differences and the retinal CSA values. Our structural models reach high resolution of the chromophore and permit to explore the downstream features at the chemical resolution. The color of the proteins as well as the water-protein engagement were analyzed based on the refined models, which also serve as the validations of the refined structural models.

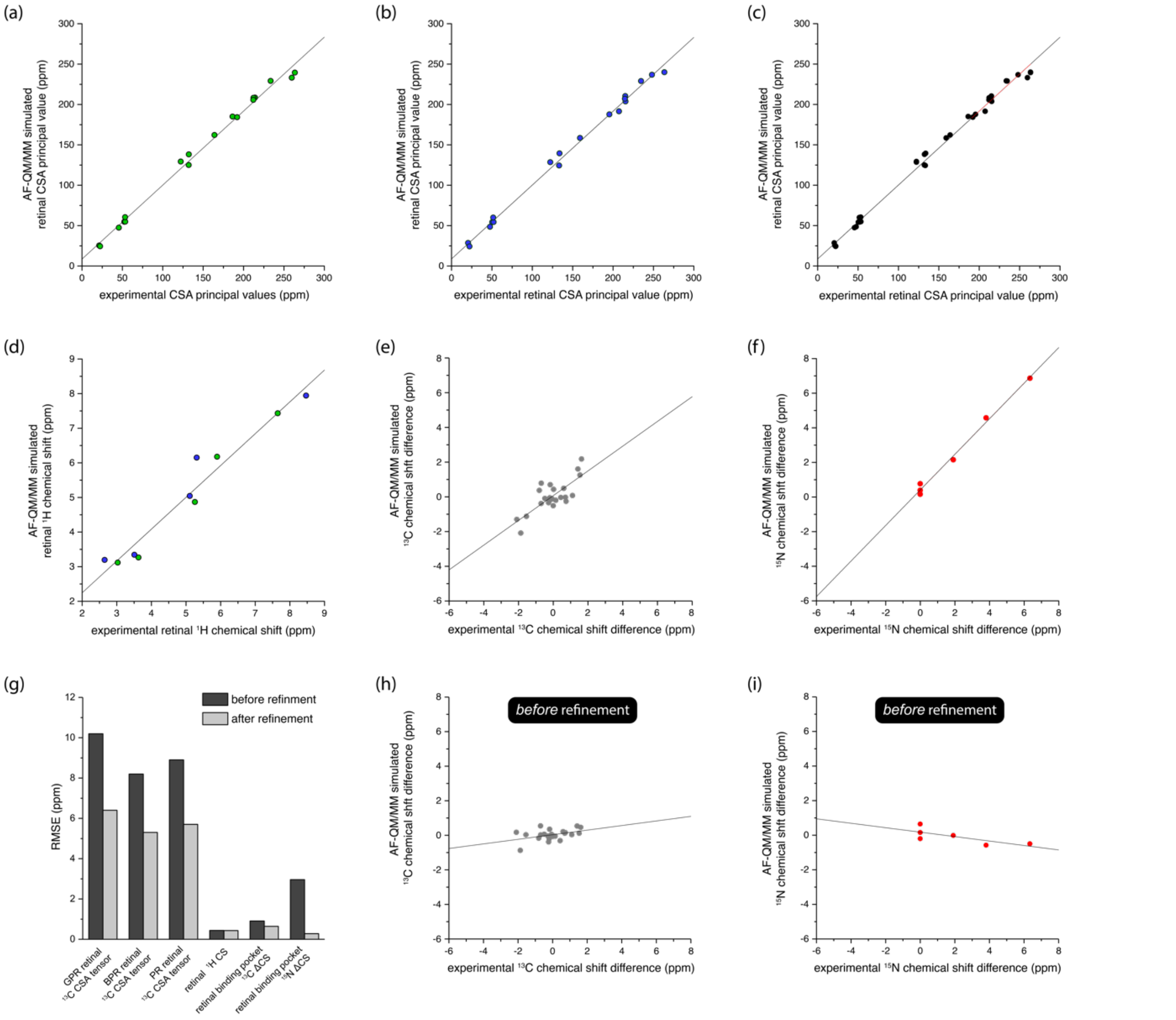

Fig. S18: Analysis of the calculated chemical shift parameters at the end point of structural modeling. The correlation between the calculated values (vertical axis) and the experimental values (horizontal axis) are fitted using linear regressions. The quantitative results of these analyses are listed in Tab. S9 below. The calculated retinal polyene (C10-C15) ^{13}C CSA principal values in GPR **(a)** and BPR **(b)** match with the experimental values rather well. All the retinal polyene ^{13}C CSA principal values are also co-presented in **(c)**. The comparison of calculated and experimental retinal polyene ^{1}H chemical shift values (H10-H15) are shown in **(d)**, in which the GPR and BPR values are shown in green and blue, respectively. The results of protein ^{13}C and ^{15}N chemical shift differences between GPR and BPR are shown in **(e)** and **(f)**, respectively. For clarity, only the chemical shift differences of retinal binding pocket residues are shown in these two panels. **(g)** Comparison of experiment-AF QM/MM chemical shift deviations before and after structural remodeling. **(h-i)** Comparisons of experimental and AF-QM/MM calculated retinal binding pocket ^{13}C and ^{15}N chemical shift differences without NMR-guided structural remodeling.

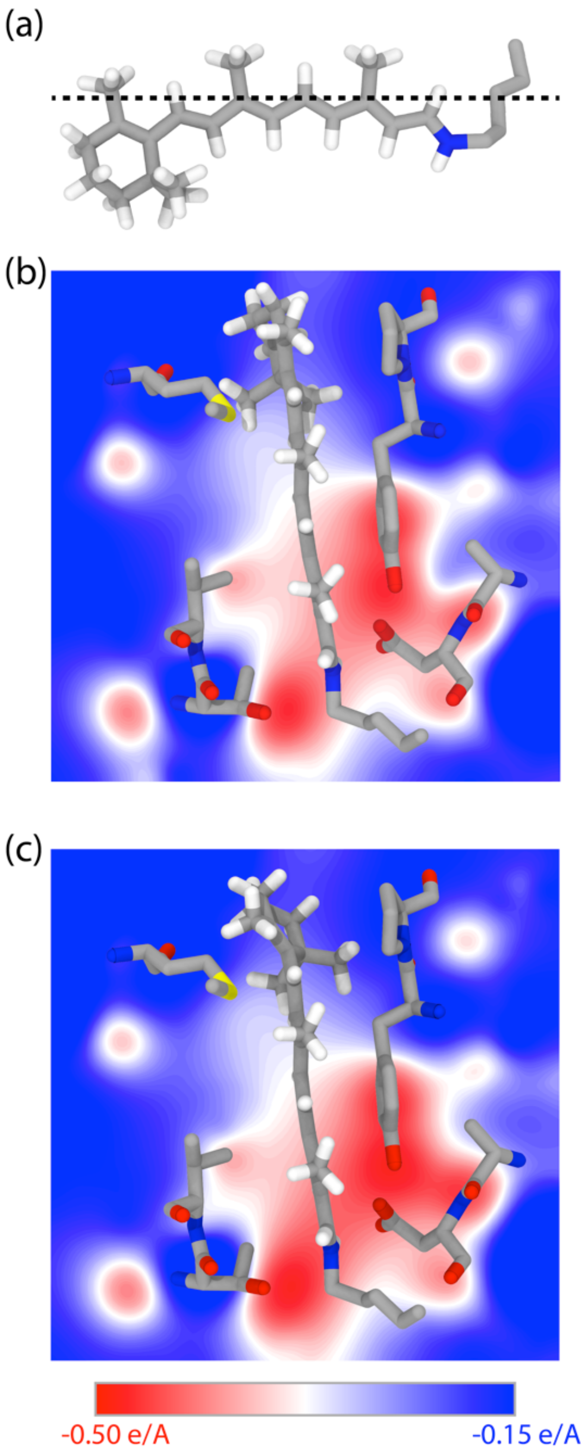

Fig. S19: A "top-view" **(a)** of protein electric field surrounding the retinal chromophore in GPR **(b)** and BPR **(c)**. The spatial distribution of E-field is presented via the color-encoded electrostatic potential. The more negative potential surrounding the pSB site is mainly generated by the counter ions.

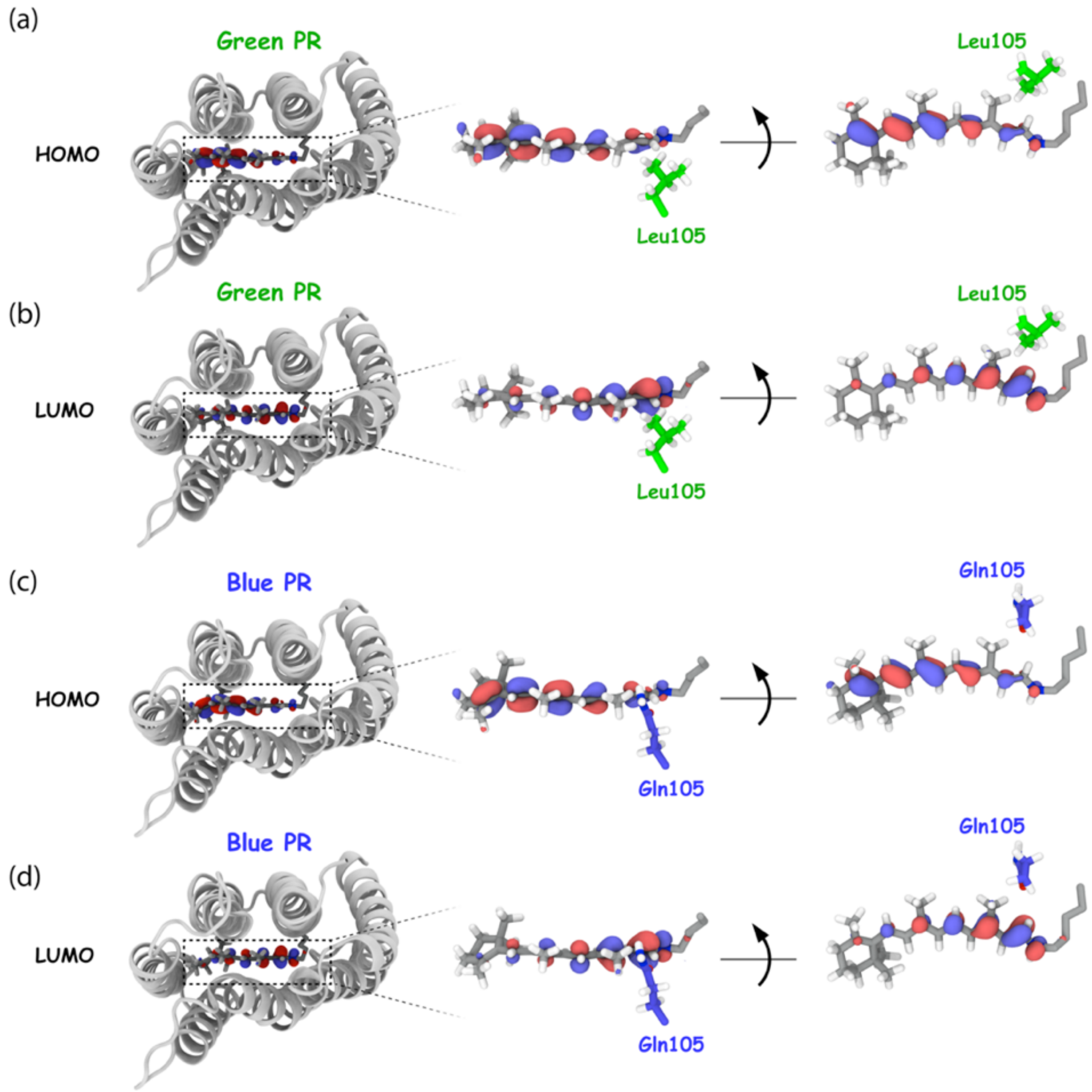

Fig. S20: HOMO **(a, c)** and LUMO **(b, d)** of the retinal chromophore in GPR **(a, b)** and BPR **(c, d)**. These orbitals were calculated from our NMR-guided QM/MM structural models. The residues Leu105 and Gln105 are highlighted. The orbitals are shown in both a side-view and a top-view. The HOMO is mainly distributed at the ionone-ring site of the retinal chromophore and the LUMO is mainly located at the pSB site of the chromophore. The dislocation of LUMO beyond the pSB site promotes the charge distribution from pSB to the polyene chain in the excited state.

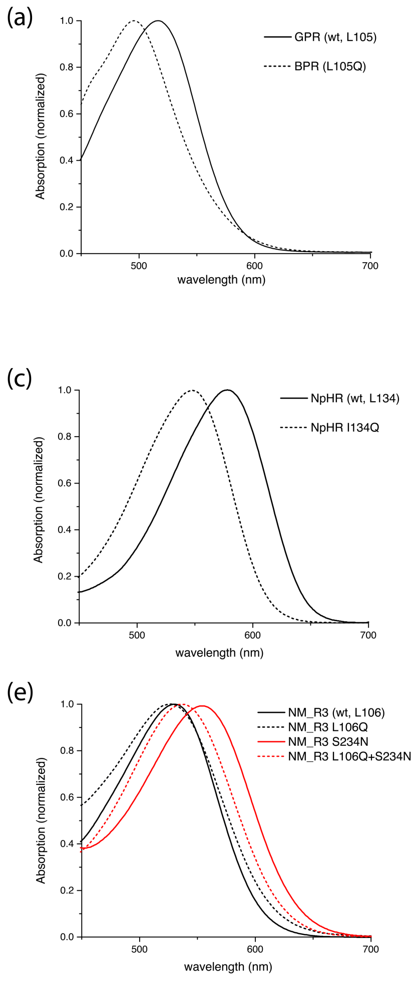

Fig. S21: UV-Vis absorption spectra of various microbial rhodopsins and their mutants. In comparison to the color mutation Leu105Gln in PRs (**(a)**, pH = 9.0), the corresponding Leu to Gln mutation in ESR only induces smaller color tuning effect (**(b)**, pH = 11.0). The color tuning effect is drastic in NpHR (**(c)**, pH = 8.5). In contrast, this mutation is largely ineffective in NM_R2 (**(d)**, pH = 11.3 to 11.4), and NM_R3 (**(e)** black curves, pH = 7.0). After introducing the Ser234Asn mutation, which corresponds to the position 230 in PRs, the color tuning effect of Leu106Gln mutation is amplified clearly (**(e)**, red curves, pH = 9.0). Similarly, the Asn230Ser mutation reduces the color-tuning effect of Leu105Gln mutation in PRs **(f)**. All spectra have been obtained above the pKa of counter ion in order to be consistent with PRs in this work.

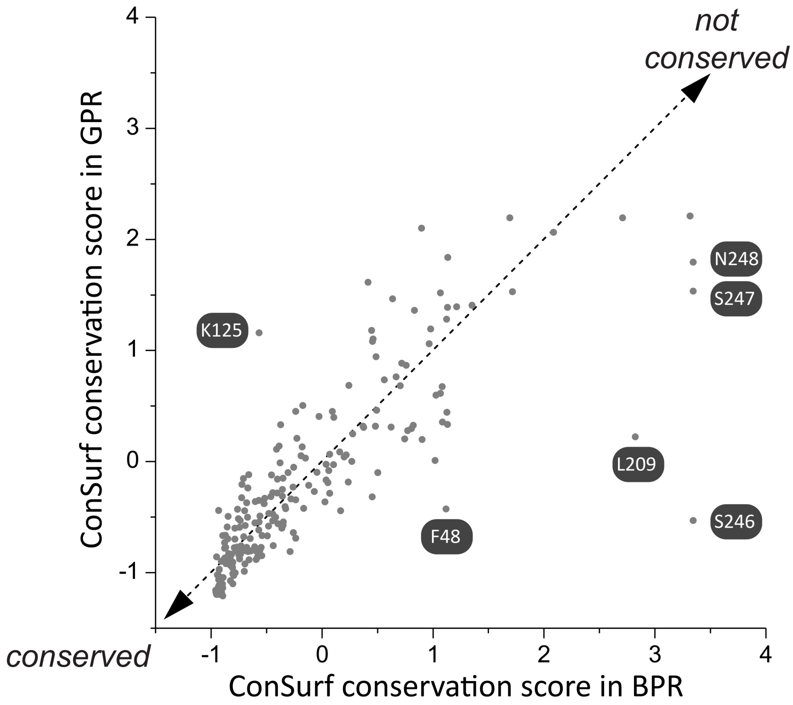

Fig. S22: Analysis of the degree of conservations of each GPR and BPR residue site using the ConSurf server (*26, 27*) to illustrate conservation differences between both groups (residue type taken from GPR, UniProt Q9F7P4). Residue K125 is more conserved in BPR than in GPR. Residues F48, L209 and S246 are more conserved in GPR than in BPR. All these residues are located outside of the retinal binding pocket, on the protein surface or in the regions that are known to be unperturbed by the color switch, lacking (*15*) and are therefore unlikely to contribute to the color tuning.

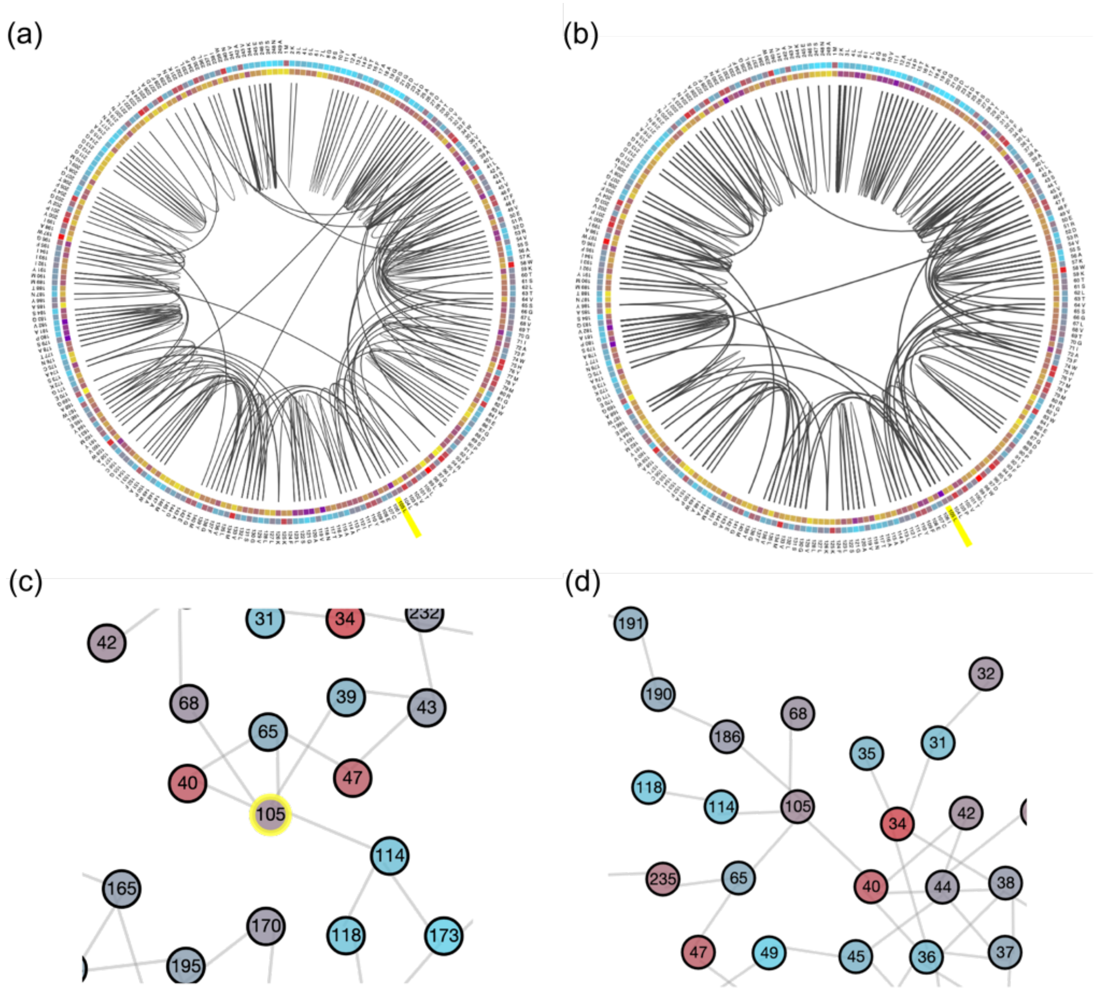

Fig. S23: Inter-residue coevolution pattern of PRs obtained by MISTIC2 (*28*). BPR and GPR sequences were analyzed together. **(a)** String plot of inter-residue coevolution pattern in PRs obtained using the plmDCA model. The threshold for the data presentation is 0.006%. **(b)** String plot of inter-residue coevolution pattern in PRs obtained using the GaussianDCA model. The threshold for the data presentation is 0.005%. **(c)** Network plot of inter-residue coevolution pattern in PRs obtained using the plmDCA model. The threshold for the data presentation is 0.007%. **(d)** Network plot of inter-residue coevolution pattern in PRs obtained using the GaussianDCA model. The threshold for the data presentation is 0.007%. No strong co-evolution pattern occurs for the color switch residue. A few residues, including residue 39, 40, 65, 68, 114 and 186, show limited co-evolution with the color switch. Similar to the residues identified by ConSurf scores (Fig. S22), these residues are also unlikely to contribute to the color tuning based on the structure and our previous NMR work (*15, 18*).

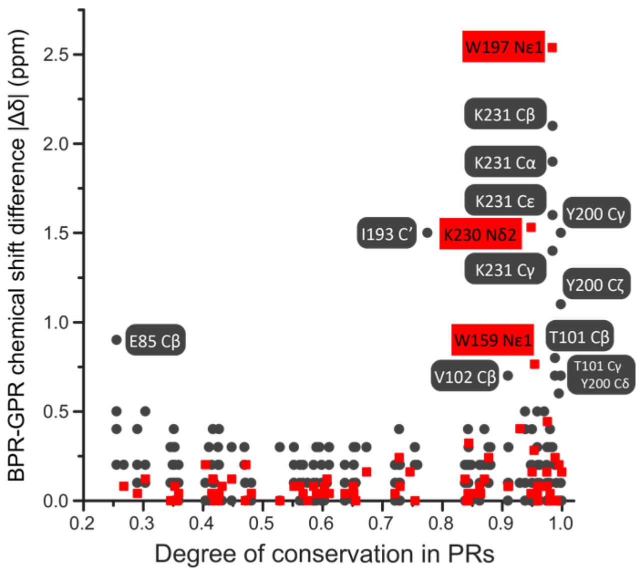

Fig. S24: Correlation of the observed BPR-GPR chemical shift differences with the degree of conservation of the affected residues within the PR family (GPRs and BPRs). The degree of conservation on the x-axis corresponds to Fig. 1d. An alternative representation is shown in Fig. 4i in which the chemical shift differences are plotted against ConSurf conservation score (see Fig. S22). Larger chemical shift differences are observed for residues with a higher degree of conservation. Black dots represent ^{13}C chemical shift changes, red squares show ^{15}N chemical shift changes scaled by gyromagnetic ratios.

Supporting Tables

Table S1: Summary of ssNMR samples and experiments in this work

Protein*	Retinal	Protein labeling	ssNMR experiment †
[$^{13}C_{10-18}$-ret, U-^{15}N]	$^{13}C_{10-18}$	U-^{15}N	^{13}C-^{13}C PDSD (Fig. 2D, S2, S7c), ^{1}H-^{13}C HETCOR (Fig. 3E, Fig. S3), ^{1}H-^{13}C D-HSQC (Fig. 2D), ^{13}C-^{13}C DQ-SQ (Fig. S7a-b), ^{13}C DQF RNCSA (Fig. S4d,e,g), DCP-filtered ^{13}C RNCSA (Fig. S4h), DCP-filtered ^{15}N RNCSA (Fig. 2B, Fig. S4a), ^{1}H-^{13}C hNOE (Fig. 4B), ^{15}N-^{13}C TEDOR/TEDOR-DQF (Fig. S12c,d)
[$^{13}C_{12,13,20}$-ret, U-^{15}N]	$^{13}C_{12,13,20}$	U-^{15}N	^{13}C DQF RNCSA (Fig. S4f), ^{15}N-^{13}C TEDOR (Fig. S13c), ^{1}H-^{13}C hNOE (Fig. S8)
[U-^{13}C, ^{15}Nε-Lys]	not labeled	U-[^{13}C, ^{15}Nε]-Lys	^{1}H-^{15}N HETCOR (Fig. S6)
[U-^{13}C, U-^{15}N, rev-His]	not labeled	U-^{13}C, U-^{15}N, His reverse labelled	^{15}N-^{13}C DCP (long contact time, Fig. S9a), NεCδ (Fig. S9b, S10a) and Nε(Cδ)CX (Fig. S10b)
[U-^{13}C Tyr, ^{15}N-Pro]	not labeled	^{15}N-Pro, U-^{13}C Tyr	^{15}N-^{13}C N(CO)CX (Fig. S11c)
[$^{13}C_{12,13,20}$-ret, U-^{13}C Tyr]	$^{13}C_{12,13,20}$	U-^{13}C Tyr	SIMPLE (hNOE-PDSD, Fig. S11d)
[U-^{13}C Tyr, U-^{15}N]	not labeled	U-^{15}N, U-^{13}C Tyr	^{15}N-^{13}C DCP (long contact time, Fig. S15b)
[U-^{13}C Val, ^{15}N-Pro]	not labeled	^{15}N-Pro, U-^{13}C Val	^{15}N-^{13}C N(CO)CX (Fig. S14)
[^{13}C'-Ile,^{15}Nε-Trp]	not labeled	^{13}C'-Ile , $^{15}N_{\varepsilon1}$-Trp	^{15}N-^{13}C TEDOR (Fig. S13a)
[^{13}C'-Ile,^{15}Nε-Trp]	3,4-deH_2 retinal	^{13}C'-Ile , $^{15}N_{\varepsilon1}$-Trp	^{15}N-^{13}C TEDOR (Fig. 3D)
[^{13}C'-Ile,^{15}Nε-Trp] I193V	not labeled	^{13}C'-Ile , $^{15}N_{\varepsilon1}$-Trp	^{15}N-^{13}C TEDOR (Fig. S13b)
[$^{13}C_{12,13,20}$-ret, U-^{15}N] N230S	$^{13}C_{12,13,20}$	U-^{15}N	^{15}N-^{13}C TEDOR (Fig. 3D)

* Each isotope labeling scheme was applied for both GPR and BPR (GPR L105Q mutant) in this work.
† The high-field MAS ssNMR experiments performed at room temperature are labeled in red. All DNP MAS ssNMR experiments are labeled in black bold fonts.

Table S2: ^{13}C, ^{1}H and ^{15}N chemical shift* / chemical shift anisotropy (CSA) of retinal chromophore in GPR and BPR

Position	Protein	Chemical shift (ppm)	CSA† (ppm)	η‡	δ_{11} (ppm)	δ_{22} (ppm)	δ_{33} (ppm)
C15	GPR	164.0	-118.3 ± 1.0	0.62 ± 0.03	259.8 ± 2.6	186.5 ± 2.0	45.7 ± 1.0
	BPR	167.8	-120.1 ± 0.6	0.34 ± 0.02	248.3 ± 1.9	207.4 ± 1.6	47.7 ± 1.0
H15	GPR	7.7	-	-	-	-	-
	BPR	8.5	-	-	-	-	-
C14	GPR	122.4	69.8 ± 0.6	1.00 ± 0.03	192.2 ± 0.6	122.4 ± 1.1	52.6 ± 1.6
	BPR	122.8	72.8 ± 0.6	0.99 ± 0.04	195.6 ± 0.6	122.4 ± 1.4	50.4 ± 2.0
H14	GPR	5.9	-	-	-	-	-
	BPR	5.3	-	-	-	-	-
C13	GPR	166.4	-145.0 ± 2.0	0.34 ± 0.15	263.6 ± 12.4	214.3 ± 11.7	21.4 ± 2.0
	BPR	166.6	-145.9 ± 2.1	0.33 ± 0.16	263.6 ± 13.3	215.5 ± 12.5	20.7 ± 2.1
C12	GPR	132.8	79.6 ± 0.8	0.99 ± 0.03	212.4 ± 0.8	132.4 ± 1.2	53.6 ± 2.0
	BPR	133.8	81.5 ± 1.0	1.00 ± 0.04	215.3 ± 1.0	133.8 ± 1.6	52.3 ± 2.6
H12	GPR	3.0	-	-	-	-	-
	BPR	2.7	-	-	-	-	-
C11	GPR	140.1	-117.6 ± 0.9	0.59 ± 0.02	233.6 ± 1.9	164.2 ± 1.4	22.5 ± 0.9
	BPR	138.8	-116.4 ± 0.9	0.65 ± 0.02	234.8 ± 1.9	159.2 ± 1.3	22.4 ± 0.9
H11	GPR	5.3	-	-	-	-	-
	BPR	5.1	-	-	-	-	-
C10	GPR	132.7	79.6 ± 0.8	0.99 ± 0.03	212.3 ± 0.8	132.3 ± 1.2	53.5 ± 2.0
	BPR	133.2	81.5 ± 1.0	1.00 ± 0.04	214.7 ± 1.0	133.2 ± 1.6	51.7 ± 2.6
H10	GPR	3.6	-	-	-	-	-
	BPR	3.5	-	-	-	-	-
pSB ^{15}N	GPR	182.1	-141.8 ± 2.8	0.73 ± 0.04	304.8 ± 5.3	201.2 ± 3.3	40.3 ± 2.8
	BPR	182.1	-138.1 ± 2.3	0.83 ± 0.03	308.5 ± 4.2	193.8 ± 2.3	44.0 ± 2.3
pSB ^{1}H	GPR	12.7	-	-	-	-	-
	BPR	11.9	-	-	-	-	-
C20	GPR	16.2	-	-	-	-	-
	BPR	15.7	-	-	-	-	-
C18	GPR	23.4	-	-	-	-	-
	BPR	23.3	-	-	-	-	-
C16/C17	GPR	27.9/32.6	-	-	-	-	-
	BPR	28.7/33.2	-	-	-	-§	-

* ^{15}N chemical shifts are referenced indirectly to liquid ammonia (25°C) and ^{13}C chemical shift are referenced indirectly to DSS (sodium trimethylsilylpropanesulfonate).

† A threshold of 105% and 102% of minimal χ^2 is used for defining the error range of ^{13}C and ^{15}N CSA respectively

‡ A threshold of 105% of minimal χ^2 is used for defining the error range of ^{13}C η value respectively. The ^{15}N η value has been derived by fitting the recoupled peak splitting and its error range is defined by the range of 98-102% of the experimental peak splitting.

Table S3: Comparison of retinal ^{1}H chemical shifts obtained by different ssNMR methods

ssNMR method	protein	chemical shift (ppm)				
		H10	H11	H12	H14	H15
HETCOR*	GPR	3.9	5.5	3.4	6.0	7.6
(^{1}H offset 6.1 ppm)	BPR	3.7	5.2	3.0	5.4	8.4
	$\Delta_{BPR-GPR}$	-0.2	-0.3	-0.4	-0.6	0.8
D-HSQC	GPR	3.6	5.3	3.0	5.9	7.7
(VFMAS)	BPR	3.5	5.1	2.7	5.3	8.5
	$\Delta_{BPR-GPR}$	-0.2	-0.2	-0.3	-0.6	0.8

* ^{1}H offset is set to 6.1 ppm

Table S4: Summary of acquisition and processing parameters for DNP ssNMR experiments*

Experiment	Spectral window (F2 * F1 in ppm)	Acquisition matrix (F2 * F1)	Processing matrix (F2 * F1)	Window functions§ (F2, F1)
^{15}N RNCSA (DCP-filtered)	500 * 222†	2048 * 64‡	2048 * 1024	GM (lb -50, gb 0.04), GM (lb -100, gb 0.06)
^{1}H-^{15}N HETCOR	398 * 33.1†	512 * 128	2048 * 1024	GM (lb -50, gb 0.04), Qsine (ssb 2)
^{13}C-^{13}C DQ-SQ	405 * 556	1600 * 128	8192 * 1024	GM (lb -20, gb 0.1), Qsine (ssb 4)
^{13}C RNCSA (DQF, DCP)	296 * 257†	1024 * 64‡	4096 * 1024	GM (lb -50, gb 0.04), GM (lb -150, gb 0.1)
^{1}H-^{13}C hNOE, SIMPLE	405 (1D)	2048 (1D)	8192 (1D)	GM (lb -50, gb 0.1)
N(C)CX	405 * 197	2048 * 40	8192 * 1024	GM (lb -40, gb 0.1), GM (lb -20, gb 0.1)
	405 * 74	2048 * 24	8192 * 1024	GM (lb -40, gb 0.1), GM (lb -20, gb 0.1)
	405 * 74	2048 * 16	8192 * 1024	GM (lb -40, gb 0.1), Qsine (ssb 3)
DCP NεCδ	405 * 197	2048 * 40	8192 * 1024	GM (lb -40, gb 0.1), GM (lb -20, gb 0.1)
Long contact-time	405 * 197	1024 * 80	8192 * 1024	GM (lb -30, gb 0.04), Qsine (ssb 3)
^{15}N-^{13}C DCP	405 * 49	1024 * 40	8192 * 1024	GM (lb -30, gb 0.04), Qsine (ssb 3)
	405 * 123	1024 * 96	8192 * 1024	GM (lb -30, gb 0.04), Qsine (ssb 4)
^{15}N-^{13}C TEDOR	405 * 49	2048 * 48	8192 * 1024	GM (lb -20, gb 0.04), Qsine (ssb 4)
	405 * 49	2048 * 48	8192 * 1024	GM (lb -20, gb 0.04), Qsine (ssb 4)
	259 * 99	1024 * 40	8192 * 1024	EM (lb -100), Qsine (ssb 3)
^{15}N-^{13}C TEDOR-DQF	259 * 99	1024 * 40	8192 * 1024	EM (lb -100), Qsine (ssb 3)

* F2 and F1 denotes the direct and indirect dimensions respectively

† The spectral window of F1 dimension is back-calculated via respective theoretical scaling factors (0.446 for $RN10_1^4$, 0.578 for FSLG, 0.310 for $R12_1^4$).

‡ Total number of hypercomplex points, which corresponds to 32 non-zero FIDs.

§ GM denotes the Gaussian window function. EM denotes the exponential window function.

Table S5: Size of QM core/QM buffer/MM regions in GPR for selected core regions*

Example	QM core region identity	QM core region size	QM buffer region size	MM protein region size	MM lipid/solvent region size §
1	retinal (C10-to-C15 segment and C20 methyl) and Lys231†	H 19, C 13, N 2, O 1 (total **35 atoms**)	H 166, C 112, N 20, O 26 (total **324 atoms**)	H 1589, C 1066, N 242, O 275, S12 (total **3184 atoms**)	H 35328, C 5680, N 144, O 13280, Na 18, P 160, Cl 16 (total **54626 atoms**)
2	Ile193 ‡	H 11, C 6, N 1, O 1 (total **19 atoms**)	H 137, C 106, N 18, O 18, S 3 (total **282 atoms**)	H 1626, C 1079, N 245, O 283, S9 (total **3242 atoms**)	same as above

* For comparison, the conventional full-system QM treatment of GPR would include **3543 atoms** (1774 hydrogens, 1191 carbons, 302 oxygens, 264 nitrogens and 12 sulfurs), which is 2-3 orders of magnitude larger than the size of QM core regions and 1 order of magnitude larger than the total QM regions (core and buffer) treated in the AF-QM/MM method.

† The largest QM core region treated in this work

‡ The large-sized retinal chromophore-Lys231 moiety is not included in the QM buffer region for Ile193

§ MM lipid/solvent region contains 144 DMPC, 16 DMPA, 18 Na^{+}, 16 Cl^{-} and 12000 H_2O molecules

Table S6: Comparison of experimental and DFT simulated chemical shift differences of retinal binding pocket residues*

residue	position	experimental Δδ† (ppm)	DFT simulated Δδ† (ppm)
Asp97	Cγ (Fig. S9)	-0.1	-0.1
Trp98	Nε1 (Fig. S9)	0.0	0.4
Trp98	Cδ1 (Fig. S9)	0.4	0.0
Thr101	C' (*15*)	0	-0.5
Thr101	Cα (*15*)	-0.2	-0.1
Thr101	Cβ (*15*)	0.8	0.4
Thr101	Cγ2 (*15*)	-0.7	-0.4
Val102	C' (Fig. S14)	-0.2	-0.3
Val102	Cα (Fig. S14)	-0.1	0.7
Val102	Cβ (Fig. S14)	0.0	0.4
Val102	Cγ (Fig. S14)	0.7	-0.3
Pro103	N (Fig. S14)	0.0	-0.2
Trp159	Nε1 (Fig. S13)	1.9	2.2
Ile192	C' (Fig. S13)	-0.5	-0.1
Ile193	C' (Fig. S13)	-1.5	-1.1
Trp197	Nε1 (Fig. S13)	6.3	6.9
Tyr200	Cζ (Fig. S11)	1.1	0.1
Tyr200	Cε (Fig. S11)	-0.7	0.8
Tyr200	Cδ (Fig. S11)	0.7	0.0
Tyr200	Cγ (Fig. S11)	1.5	1.3
Tyr200	Cα (Fig. S11)	0.1	-0.2
Pro201	N (Fig. S11)	0.0	0.8
Asp227	Cγ (Fig. S9)	0.6	0.5
Asn230	Nδ2 (Fig. S15)	3.8	4.6
Lys231	Cα (Fig. S10)	-1.9	-2.1
Lys231	Cβ (Fig. S10)	-2.1	-1.3
Lys231	Cγ (Fig. S10)	1.4	1.6
Lys231	Cδ (Fig. S10)	-0.3	-0.3
Lys231	Cε (Fig. S10)	1.6	2.2

* DFT simulated chemical shift parameters were obtained on the refined structures

† Defined as δ(GPR) – δ(BPR)

Table S7: Comparison of experimental and DFT simulated retinal chemical shift values*†

position	experimental chemical shift (ppm)	simulated chemical shift (ppm)	experimental CSA (ppm)	simulated CSA (ppm)	experimental η	simulated η	experimental δ_{11} (ppm)	simulated δ_{11} (ppm)	experimental δ_{22} (ppm)	Simulated δ_{22} (ppm)	experimental δ_{33} (ppm)	simulated δ_{22} (ppm)
H15	7.7 (8.5)	7.4 (7.9)	-	-	-	-	-	-	-	-	-	-
C15	164.0 (167.8)		-118.3 (-120.1)	-107.4 (-110.5)	0.62 (0.34)	0.44 (0.41)	259.8 (248.3)	233.0 (236.7)	186.5 (207.4)	184.9 (191.4)	45.7 (47.7)	47.3 (48.4)
H14	5.9 (5.3)	6.2 (6.1)	-	-	-	-	-	-	-	-	-	-
C14	122.4 (122.8)		69.8 (72.8)	68.1 (69.2)	1.00 (0.99)	0.81 (0.86)	192.2 (195.6)	184.2 (187.1)	122.4 (122.4)	129.2 (128.4)	52.6 (50.4)	54.6 (54.2)
C13	166.4 (166.6)		-145.0 (-145.9)	-132.5 (-128.9)	0.34 (0.33)	0.23 (0.28)	263.6 (263.6)	239.4 (239.9)	214.3 (215.5)	208.4 (203.6)	21.4 (20.7)	25.2 (28.4)
H12	3.0 (2.7)	3.1 (3.2)	-		-	-	-	-	-	-	-	-
C12	132.8 (133.8)		79.6 (81.5)	78.9 (80.4)	0.99 (1.00)	0.89 (0.88)	212.4 (215.3)	208.1 (210.2)	132.4 (133.8)	138.1 (139.4)	53.6 (52.3)	54.8 (54.3)
H11	5.3 (5.1)	4.9 (5.0)	-		-	-	-	-	-	-	-	-
C11	140.1 (138.8)		-117.6 (-116.4)	-114.2 (-113.1)	0.59 (0.65)	0.59 (0.62)	233.6 (234.8)	229.0 (229.0)	164.2 (159.2)	162.1 (158.4)	22.5 (22.4)	24.3 (24.1)
H10	3.6 (3.5)	3.3 (3.3)	-		-	-	-	-	-	-	-	-
C10	132.7 (133.2)		79.6 (81.5)	69.9 (70.5)	0.99 (1.00)	0.84 (0.84)	212.3 (214.7)	205.5 (207.0)	132.3 (133.2)	125.0 (124.3)	53.5 (51.7)	60.4 (59.9)
C20	16.2 (15.7)	24.9 (23.4)	-	-	-	-	-	-	-	-	-	-

* DFT simulated chemical shift parameters were obtained on the refined structures

Table S8: Bond lengths of refined retinal polyene chain*

bond	Bond length (Å)	
	GPR	BPR
C5=C6	1.368	1.367
C6-C7	1.506	1.506
C7=C8	1.345	1.345
C8-C9	1.478	1.478
C9=C10	1.342	1.342
C10-C11	1.464	1.474
C10-H10	1.103	1.103
C11=C12	1.340	1.340
C11-H11	1.122	1.102
C12-C13	1.466	1.476
C12-H12	1.104	1.104
C13=C14	1.347	1.342
C13-C20 (methyl)	1.510	1.539
C14-C15	1.435	1.461
C14-H14	1.114	1.102
C15=pSB N	1.285	1.279
C15-H15	1.102	1.117
pSB N-Lys231 Cε	1.478	1.458
pSB N-H	1.008	0.981

* Bonds composing conjugated retinal polyene chain are highlighted in gray.

Table S9: Summary of the AF-QM/MM structural modeling results

parameter	root-MSE (ppm)	data range* (ppm)	slope	intercept (ppm)	reduced R^2	data visualization (Fig. S21)
GPR retinal CSA	6.4	83.8 (77.0)	0.92 ± 0.02	8.6 ± 3.0	0.993	a
BPR retinal CSA	5.3	84.4 (74.4)	0.91 ± 0.01	8.8 ± 2.5	0.995	b
Retinal ^{13}C CSA (GPR+BPR)	5.7	82.9 (76.1)	0.92 ± 0.01	8.7 ± 1.9	0.994	c
Retinal ^{1}H (GPR+BPR)	0.43	1.9 (1.8)	0.92 ± 0.08	0.41 ± 0.40	0.943	d
ΔCS(^{13}C, retinal binding pocket)	0.64	1.0 (0.9)	0.71 ± 0.13‡	0.07 ± 0.12	0.589‡	e
ΔCS(^{15}N, retinal binding pocket)	0.28	2.6 (2.7)	1.03 ± 0.05	0.41 ± 0.15	0.990	f

* Defined as the standard deviation of experimental data (or of simulated values in parentheses). This parameter indicates the scale of the distribution of respect data. Small data ranges here indicate that the data are rather gathered in a small range and therefore inherently less "resolved" for the AF-QM/MM structural modeling. The similar data ranges of experimental and simulated values indicate that our AF-QM/MM simulations are capable of reproducing the chemical shift parameters to their full scale.

‡ Most ΔCS(^{13}C) is of small values as indicated by the data range*, which is only twice of root-MSE. This condition, in particular for chemical shift changes in small scale, makes the slope of correlation not a suitable primary parameter for evaluating the deviation of simulated values from the experimental readouts. Instead, the root-MSE can serve as the main parameter for this purpose.

Table S10: Comparison of experimental chemical shift parameters and QM/MM-derived values computed on PR structures without NMR-guided structural remodeling. The BPR structure (4JQ6) was used for constructing these initial structural models of PRs.

parameter	root-MSE (ppm)	data range* (ppm)	slope	intercept (ppm)	reduced R^2	data visualization (Fig. S21)
GPR retinal CSA	10.2	83.8 (76.7)	0.91 ± 0.02	8.7 ± 3.4	0.991	a
BPR retinal CSA	8.2	84.4 (78.2)	0.92 ± 0.02	7.8 ± 2.5	0.996	b
Retinal ^{13}C CSA (GPR+BPR)	8.9	82.9 (76.5)	0.92 ± 0.01	8.2 ± 2.0	0.994	c
Retinal ^{1}H (GPR+BPR)	0.44	1.9 (1.9)	0.97 ± 0.08	0.0 ± 0.40	0.943	d
ΔCS(^{13}C, retinal binding pocket)	0.91	1.0 (0.3)	0.13 ± 0.06	0.03 ± 0.06	0.147	e
ΔCS(^{15}N, retinal binding pocket)	2.96	2.6 (0.5)	-0.13 ± 0.06	0.18 ± 0.18	0.440	f

Table S11: Structural deviation of retinal binding pocket in various PR structures. The deviations were defined as RMSD (in Å) of residues in retinal binding pocket. AF2 indicates structural models generated by AlphaFold2. For the structures determined by X-ray crystallography or cryo-EM, corresponding PDB code are shown as well.

	GPR (this work)	BPR (this work)	GPR (AF2)	BPR Med12 (AF2)	BPR HOT75 (AF2)	GPR (7b03)	BPR (4jq6)	BPR (4kly)
GPR (this work)	-	0.41	0.67	0.69	0.64	0.78	0.57 – 0.74	0.69 – 0.74
BPR (this work)		-	0.75	0.75	0.68	0.84	0.58 – 0.61	0.65 – 0.75
GPR (AF2)			-	0.15	0.25	0.69	0.52 – 0.55	0.51 – 0.65
BPR Med12 (AF2)				-	0.27	0.70	0.51 – 0.53	0.48 – 0.62
BPR HOT75 (AF2)					-	0.64	0.44 – 0.46	0.50 – 0.62
GPR (7b03)						-	0.65 – 0.70	0.75 – 0.83
BPR (4jq6)							0.24 – 0.50	0.50 – 0.65
BPR (4kly)								0.28 – 0.50

Table S12: Electrostatic potential at retinal chromophore in PRs

retinal position	Electrostatic field potential* (atomic unit)†	
	GPR	BPR
C5	-0.22	-0.22
C6	-0.23	-0.23
C7	-0.26	-0.25
C8	-0.28	-0.28
C9	-0.31	-0.31
C10	-0.33	-0.33
C11	-0.35	-0.35
C12	-0.36	-0.36
C13	-0.36	-0.37
C14	-0.37	-0.39
C15	-0.38	-0.42
pSB Nζ	-0.41	-0.44
H15	-0.38	-0.43
C20	-0.35	-0.37

* Calculations have been performed using the same parameters without further calibration.

† 1 a.u = E_h/e=27.211 V

Table S13: *In-silico* analysis of PR color switching mechanism

in silico "construct"	maximum light absorption wavelength (nm)		
	GPR	BPR (ionone ring in polyene plane)	BPR (ring reoriented)
retinal in vacuum	614.7	627.1	639.5
retinal in non-polarized protein environment (- water in color switch center)**	498.5	478.8	471.8
retinal in polarized protein environment (- water in color switching center)	520.5	502.8	492.7
retinal in non-polarized protein environment (+ water in color switch center)	495.1	483.5	476.6
retinal in polarized protein environment (+ water in color switching center)***	517.1	507.5	497.5
T101 conformation exchanged	534.8	-	494.7
Y201 conformation exchanged	528.2	-	493.5
deH$_2$-retinal (additional C=C double bond on ionone ring)	554 (551)*	-	533.8 (525)*
GPR Cryo-EM Structure (7B03, (*18*))	639 (520)*	-	-
BPR X-Ray Structure (4JQ6, chain C/B, (*17*))	-	622 / 628 (500)*	622 / 628 (500)*

* Experimental values are provided in parenthesis.

** The AMBER charge model (non-polarized fixed charge model) was utilized for the non-polarized protein environment.

*** The PPC (polarized protein-specific charges) charge model was utiilized for the polarized protein environment.

Table S14: Molecular cloning of microbial rhodopsins for recombinant expression

protein	construct	native host	UniProt (wt)	function	plasmid backbone	codon optimization	tag	Cloning site
PR	wt/L105Q/N230S /L105Q,N230S	γ-proteobacterium EBAC31A08 (G- bacterim)	Q9F7P4	proton pumping	pET-27b(+)	no	C-His$_6$	NdeI-NheI
ESR	wt/ L93Q	Exiguobacterium sibiricum *DSM17290/CIP10946/JCM1349/255-15* (G+ bacterim)	B1YFV8	proton pumping	pET-21a(+)	yes	C-His$_6$	NdeI-XhoI
NM_R2	wt/L119Q	Nonlabens marinus *S1-08* (G- flavobacterium)	W8VZ79	sodium pumping	pET-21a(+)	yes	C-His$_{6/}$	NdeI-XhoI
NM_R3	wt/L106Q/S234N /L106Q,S234N	Nonlabens marinus *S1-08* (G- flavobacterium)	W8VZW3	chloride pumping	pET-21a(+)	yes	C-His$_6$	NdeI-XhoI
NpHR	wt/I134Q	Natronomonas pharaonis (archaeon)	A0A1U7EU03	chloride pumping	pET-21a(+)	no	C-His$_6$	NdeI-XhoI

Table S15: Expression and purification conditions for various microbial rhodopsins

protein	[IPTG] / mM	temperature and time	solubilization*	washing	elution
PR	0.84	27 ºC, 16 h	MES 50 mM, NaCl 300 mM, imidazol 5 mM, 1.5% DDM, pH 6.0 (16 h)	MES 50 mM, NaCl 300 mM, imidazol 50 mM, 0.15% DDM, pH 6.0	MES 50 mM, NaCl 300 mM, imidazol 500 mM, 0.05% DDM, pH 7.5
ESR	1.0	27 ºC, 16 h	Tris 50 mM, 300 mM NaCl, imidazol 5 mM, 1.0% DDM, pH 8.0 (12 h)	Tris 50 mM, 300 mM NaCl, imidazol 50 mM, 0.15% DDM, pH 7.4	Tris 50 mM, 300 mM NaCl, imidazol 500 mM, 0.10% DDM, pH 7.4
NM_R2	0.5	27 ºC, 16 h	MES 50 mM, NaCl 300 mM, imidazol 5 mM, 1.0% Triton X100, pH 6.0 (12 h)	MES 50 mM, NaCl 300 mM, imidazol 50 mM, 0.15% DDM, pH 6.0	Tris 50 mM, 300 mM NaCl, imidazol 500 mM, 0.10% DDM, pH 7.4
NM_R3	0.5	22 ºC, 12 h	Tris 50 mM, NaCl 200 mM, imidazole 5 mM, 1.0% DDM, pH 7.5 (6 h)	0.15% DDM Tris 50 mM, NaCl 200 mM, Im 25 mM, pH 7.5	0.05% DDM Tris 50 mM, NaCl 200 mM, Im 500 mM, pH 7.5

* All solubilization steps were carried out at 4 ºC. Detergent amount is in w/v for DDM and v/v for Triton X-100

REFERENCES AND NOTES

1. O. Beja, O. Béjà, L. Aravind, E. V. Koonin, M. T. Suzuki, A. Hadd, L. P. Nguyen, S. B. Jovanovich, C. M. Gates, R. A. Feldman, J. L. Spudich, E. N. Spudich, E. F. De Long, Bacterial rhodopsin: Evidence for a new type of phototrophy in the sea. *Science* **289**, 1902–1906 (2000).

2. A. Rozenberg, K. Inoue, H. Kandori, O. Beja, Microbial rhodopsins: The last two decades. *Annu. Rev. Microbiol.* **75**, 427–447 (2021).

3. V. Gordeliy, K. Kovalev, E. Bamberg, F. Rodriguez-Valera, E. Zinovev, D. Zabelskii, A. Alekseev, R. Rosselli, I. Gushchin, I. Okhrimenko, Microbial rhodopsins. *Methods in molecular biology* **2501**, 1–52 (2022).

4. C. Bamann, E. Bamberg, J. Wachtveitl, C. Glaubitz, Proteorhodopsin. *Biochim. Biophys. Acta* **1837**, 614–625 (2014).

5. D. Man, W. Wang, G. Sabehi, L. Aravind, A. F. Post, R. Massana, E. N. Spudich, J. L. Spudich, O. Béjà, Diversification and spectral tuning in marine proteorhodopsins. *EMBO J.* **22**, 1725–1731 (2003).

6. A. Pushkarev, G. Hevroni, S. Roitman, J. G. Shim, A. Choi, K. H. Jung, *O. Béjà*, The use of a chimeric rhodopsin vector for the detection of new proteorhodopsins based on color. *Front. Microbiol.* **9**, 439 (2018).

7. D. K. Olson, S. Yoshizawa, D. Boeuf, W. Iwasaki, E. F. DeLong, Proteorhodopsin variability and distribution in the North Pacific Subtropical Gyre. *ISME J.* **12**, 1047–1060 (2018).

8. M. Hoffmann, M. Wanko, P. Strodel, P. H. König, T. Frauenheim, K. Schulten, W. Thiel, E. Tajkhorshid, M. Elstner, Color tuning in rhodopsins: The mechanism for the spectral shift

between bacteriorhodopsin and sensory rhodopsin II. *J. Am. Chem. Soc.* **128**, 10808–10818 (2006).

9. K. Nakanishi, V. Baloghnair, M. Arnaboldi, K. Tsujimoto, B. Honig, An external point-charge model for bacteriorhodopsin to account for its purple color. *J. Am. Chem. Soc.* **102**, 7945–7947 (1980).

10. M. Karasuyama, K. Inoue, R. Nakamura, H. Kandori, I. Takeuchi, Understanding colour tuning rules and predicting absorption wavelengths of microbial rhodopsins by data-driven machine-learning approach. *Sci. Rep.* **8**, 15580 (2018).

11. Y. Ozaki, T. Kawashima, R. Abe-Yoshizumi, H. Kandori, A color-determining amino acid residue of proteorhodopsin. *Biochemistry* **53**, 6032–6040 (2014).

12. R. Rangarajan, J. F. Galan, G. Whited, R. R. Birge, Mechanism of spectral tuning in green-absorbing proteorhodopsin. *Biochemistry* **46**, 12679–12686 (2007).

13. J. R. Church *et al.*, Deciphering the spectral tuning mechanism in proteorhodopsin: The dominant role of electrostatics instead of chromophore geometry. *Chemistry* **28**, e202200139 (2022).

14. C. Lee, S. Sekharan, B. Mertz, Theoretical insights into the mechanism of wavelength regulation in blue-absorbing proteorhodopsin. *J. Phys. Chem. B* **123**, 10631–10641 (2019).

15. J. Mao, N. N. do, F. Scholz, L. Reggie, M. Mehler, A. Lakatos, Y. S. Ong, S. J. Ullrich, L. J. Brown, R. C. D. Brown, J. Becker-Baldus, J. Wachtveitl, C. Glaubitz, Structural basis of the green-blue color switching in proteorhodopsin as determined by NMR spectroscopy. *J. Am. Chem. Soc.* **136**, 17578–17590 (2014).

16. C. Shen, X. Jin, W. J. Glover, X. He, Accurate prediction of absorption spectral shifts of proteorhodopsin using a fragment-based quantum mechanical method. *Molecules* **26**, (2021).

17. T. Ran, G. Ozorowski, Y. Gao, O. A. Sineshchekov, W. Wang, J. L. Spudich, H. Luecke, Cross-protomer interaction with the photoactive site in oligomeric proteorhodopsin complexes. *Acta Crystallogr. D Biol. Crystallogr.* **69**, 1965–1980 (2013).

18. S. Hirschi, D. Kalbermatter, Z. Ucurum, T. Lemmin, D. Fotiadis, Cryo-EM structure and dynamics of the green-light absorbing proteorhodopsin. *Nat. Commun.* **12**, 4107 (2021).

19. X. He, T. Zhu, X. Wang, J. Liu, J. Z. Zhang, Fragment quantum mechanical calculation of proteins and its applications. *Acc. Chem. Res.* **47**, 2748–2757 (2014).

20. Q. Z. Ni, E. Daviso, T. V. Can, E. Markhasin, S. K. Jawla, T. M. Swager, R. J. Temkin, J. Herzfeld, R. G. Griffin, High frequency dynamic nuclear polarization. *Acc. Chem. Res.* **46**, 1933–1941 (2013).

21. T. Biedenbander, V. Aladin, S. Saeidpour, B. Corzilius, Dynamic nuclear polarization for sensitivity enhancement in biomolecular solid-state NMR. *Chem. Rev.* **122**, 9738–9794 (2022).

22. W. Y. Chow, G. De Paepe, S. Hediger, Biomolecular and biological applications of solid-state NMR with dynamic nuclear polarization enhancement. *Chem. Rev.* **122**, 9795–9847 (2022).

23. A. Pushkarev, O. Beja, Functional metagenomic screen reveals new and diverse microbial rhodopsins. *ISME J.* **10**, 2331–2335 (2016).

24. J. Feng, B. Mertz, Proteorhodopsin activation is modulated by dynamic changes in internal hydration. *Biochemistry* **54**, 7132–7141 (2015).

25. J. Mao, V. Aladin, X. Jin, A. J. Leeder, L. J. Brown, R. C. D. Brown, X. He, B. Corzilius, C. Glaubitz, Exploring protein structures by DNP-enhanced methyl solid-state NMR spectroscopy. *J. Am. Chem. Soc.* **141**, 19888–19901 (2019).

26. H. Ashkenazy, S. Abadi, E. Martz, O. Chay, I. Mayrose, T. Pupko, N. Ben-Tal, ConSurf 2016: An improved methodology to estimate and visualize evolutionary conservation in macromolecules. *Nucleic Acids Res.* **44**, W344–350 (2016).

27. C. Berezin, F. Glaser, J. Rosenberg, I. Paz, T. Pupko, P. Fariselli, R. Casadio, N. Ben-Tal, ConSeq: The identification of functionally and structurally important residues in protein sequences. *Bioinformatics* **20**, 1322–1324 (2004).

28. E. A. Colell, J. A. Iserte, F. L. Simonetti, C. Marino-Buslje, MISTIC2: Comprehensive server to study coevolution in protein families. *Nucleic Acids Res.* **46**, W323-W328 (2018).

29. G. Hou, I. J. Byeon, J. Ahn, A. M. Gronenborn, T. Polenova, Recoupling of chemical shift anisotropy by R-symmetry sequences in magic angle spinning NMR spectroscopy. *J. Chem. Phys.* **137**, 134201 (2012).

30. R. D. Curtis, G. H. Penner, W. P. Power, R. E. Wasylishen, Dipolar-chemical shift NMR spectra of the carbon-nitrogen linkage in benzylideneaniline: Carbon and nitrogen chemical shielding anisotropies. *J. Phys. Chem.* **94**, 4000–4006 (2002).

31. H. Luecke, B. Schobert, H. T. Richter, J. P. Cartailler, J. K. Lanyi, Structure of bacteriorhodopsin at 1.55 A resolution. *J. Mol. Biol.* **291**, 899–911 (1999).

32. V. Aladin, M. Vogel, R. Binder, I. Burghardt, B. Suess, B. Corzilius, Complex formation of the tetracycline-binding aptamer investigated by specific cross-relaxation under DNP. *Angew. Chem. Int. Ed. Engl.* **58**, 4863–4868 (2019).

33. D. Daube, V. Aladin, J. Heiliger, J. J. Wittmann, D. Barthelmes, C. Bengs, H. Schwalbe, B. Corzilius, Heteronuclear cross-relaxation under solid-state dynamic nuclear polarization. *J. Am. Chem. Soc.* **138**, 16572–16575 (2016).

34. M. M. Hoffmann, S. Bothe, T. Gutmann, F. F. Hartmann, M. Reggelin, G. Buntkowsky, Directly vs indirectly enhanced13c in dynamic nuclear polarization magic angle spinning NMR experiments of nonionic surfactant systems. *J. Phys. Chem. C* **121**, 2418–2427 (2017).
35. A. Bertarello, L. Benda, K. J. Sanders, A. J. Pell, M. J. Knight, V. Pelmenschikov, L. Gonnelli, I. C. Felli, M. Kaupp, L. Emsley, R. Pierattelli, G. Pintacuda, Picometer resolution structure of the coordination sphere in the metal-binding site in a metalloprotein by NMR. *J. Am. Chem. Soc.* **142**, 16757–16765 (2020).
36. A. Klein, P. Rovó, V. V. Sakhrani, Y. Wang, J. B. Holmes, V. Liu, P. Skowronek, L. Kukuk, S. K. Vasa, P. Güntert, L. J. Mueller, R. Linser, Atomic-resolution chemical characterization of (2x)72-kDa tryptophan synthase via four- and five-dimensional (1)H-detected solid-state NMR. *Proc. Natl. Acad. Sci. U.S.A.* **119**, (2022).
37. J. B. Holmes, V. Liu, B. G. Caulkins, E. Hilario, R. K. Ghosh, V. N. Drago, R. P. Young, J. A. Romero, A. D. Gill, P. M. Bogie, J. Paulino, X. Wang, G. Riviere, Y. K. Bosken, J. Struppe, A. Hassan, J. Guidoulianov, B. Perrone, F. Mentink-Vigier, C. E. A. Chang, J. R. Long, R. J. Hooley, T. C. Mueser, M. F. Dunn, L. J. Mueller, Imaging active site chemistry and protonation states: NMR crystallography of the tryptophan synthase α-aminoacrylate intermediate. *Proc. Natl. Acad. Sci. U.S.A.* **119**, (2022).
38. J. Swails, T. Zhu, X. He, D. A. Case, AFNMR: Automated fragmentation quantum mechanical calculation of NMR chemical shifts for biomolecules. *J. Biomol. NMR* **63**, 125–139 (2015).
39. X. He, B. Wang, K. M. Merz, Jr., Protein NMR chemical shift calculations based on the automated fragmentation QM/MM approach. *J. Phys. Chem. B* **113**, 10380–10388 (2009).

40. J. Jumper, R. Evans, A. Pritzel, T. Green, M. Figurnov, O. Ronneberger, K. Tunyasuvunakool, R. Bates, A. Žídek, A. Potapenko, A. Bridgland, C. Meyer, S. A. A. Kohl, A. J. Ballard, A. Cowie, B. Romera-Paredes, S. Nikolov, R. Jain, J. Adler, T. Back, S. Petersen, D. Reiman, E. Clancy, M. Zielinski, M. Steinegger, M. Pacholska, T. Berghammer, S. Bodenstein, D. Silver, O. Vinyals, A. W. Senior, K. Kavukcuoglu, P. Kohli, D. Hassabis, Highly accurate protein structure prediction with AlphaFold. *Nature* **596**, 583–589 (2021).

41. F. Melaccio, M. del Carmen Marín, A. Valentini, F. Montisci, S. Rinaldi, M. Cherubini, X. Yang, Y. Kato, M. Stenrup, Y. Orozco-Gonzalez, N. Ferré, H. L. Luk, H. Kandori, M. Olivucci, Toward automatic rhodopsin modeling as a tool for high-throughput computational photobiology. *J. Chem. Theory Comput.* **12**, 6020–6034 (2016).

42. A. Altun, S. Yokoyama, K. Morokuma, Spectral tuning in visual pigments: An ONIOM(QM:MM) study on bovine rhodopsin and its mutants. *J. Phys. Chem. B* **112**, 6814–6827 (2008).

43. A. Altun, S. Yokoyama, K. Morokuma, Mechanism of spectral tuning going from retinal in vacuo to bovine rhodopsin and its mutants: Multireference ab initio quantum mechanics/molecular mechanics studies. *J. Phys. Chem. B* **112**, 16883–16890 (2008).

44. S. D. Fried, S. Bagchi, S. G. Boxer, Extreme electric fields power catalysis in the active site of ketosteroid isomerase. *Science* **346**, 1510–1514 (2014).

45. M. G. Romei, C. Y. Lin, Mathews, II, S. G. Boxer, Electrostatic control of photoisomerization pathways in proteins. *Science* **367**, 76–79 (2020).

46. S. D. Fried, S. G. Boxer, Electric fields and enzyme catalysis. *Annu. Rev. Biochem.* **86**, 387–415 (2017).

47. N. M. Levinson, S. G. Boxer, A conserved water-mediated hydrogen bond network defines bosutinib's kinase selectivity. *Nat. Chem. Biol.* **10**, 127–132 (2014).

48. C. Zheng, Y. Mao, J. Kozuch, A. O. Atsango, Z. Ji, T. E. Markland, S. G. Boxer, A two-directional vibrational probe reveals different electric field orientations in solution and an enzyme active site. *Nat. Chem.* **14**, 891–897 (2022).

49. X. Wang, X. He, J. Z. Zhang, Predicting mutation-induced Stark shifts in the active site of a protein with a polarized force field. *J. Phys. Chem. A* **117**, 6015–6023 (2013).

50. T. Zhu, J. Z. Zhang, X. He, Automated fragmentation QM/MM calculation of amide proton chemical shifts in proteins with explicit solvent model. *J. Chem. Theory Comput.* **9**, 2104–2114 (2013).

51. J. Lee, X. Cheng, J. M. Swails, M. S. Yeom, P. K. Eastman, J. A. Lemkul, S. Wei, J. Buckner, J. C. Jeong, Y. Qi, S. Jo, V. S. Pande, D. A. Case, C. L. Brooks III, A. D. MacKerell Jr, J. B. Klauda, W. Im, CHARMM-GUI input generator for NAMD, GROMACS, AMBER, OpenMM, and CHARMM/OpenMM simulations using the charmm36 additive force field. *J. Chem. Theory Comput.* **12**, 405–413 (2016).

52. R. Otten, R. A. P. Pádua, H. A. Bunzel, V. Nguyen, W. Pitsawong, M. K. Patterson, S. Sui, S. L. Perry, A. E. Cohen, D. Hilvert, D. Kern, How directed evolution reshapes the energy landscape in an enzyme to boost catalysis. *Science* **370**, 1442–1446 (2020).

53. J. Zheng, N. Guo, A. Wagner, Selection enhances protein evolvability by increasing mutational robustness and foldability. *Science* **370**, (2020).

54. K. S. Sarkisyan, D. A. Bolotin, M. V. Meer, D. R. Usmanova, A. S. Mishin, G. V. Sharonov, D. N. Ivankov, N. G. Bozhanova, M. S. Baranov, O. Soylemez, N. S. Bogatyreva, P. K. Vlasov, E. S. Egorov, M. D. Logacheva, A. S. Kondrashov, D. M. Chudakov, E. V. Putintseva, I. Z.

Mamedov, D. S. Tawfik, K. A. Lukyanov, F. A. Kondrashov, Local fitness landscape of the green fluorescent protein. *Nature* **533**, 397–401 (2016).

55. R. Blomberg, H. Kries, D. M. Pinkas, P. R. E. Mittl, M. G. Grütter, H. K. Privett, S. L. Mayo, D. Hilvert, Precision is essential for efficient catalysis in an evolved Kemp eliminase. *Nature* **503**, 418–421 (2013).

56. M. Mehler, F. Scholz, S. J. Ullrich, J. Mao, M. Braun, L. J. Brown, R. C. D. Brown, S. A. Fiedler, J. Becker-Baldus, J. Wachtveitl, C. Glaubitz, The EF loop in green proteorhodopsin affects conformation and photocycle dynamics. *Biophys. J.* **105**, 385–397 (2013).

57. M. Mehler, C. E. Eckert, A. J. Leeder, J. Kaur, T. Fischer, N. Kubatova, L. J. Brown, R. C. D. Brown, J. Becker-Baldus, J. Wachtveitl, C. Glaubitz, Chromophore distortions in photointermediates of proteorhodopsin visualized by dynamic nuclear polarization-enhanced solid-state NMR. *J. Am. Chem. Soc.* **139**, 16143–16153 (2017).

58. I. Bertini, F. Engelke, L. Gonnelli, B. Knott, C. Luchinat, D. Osen, E. Ravera, On the use of ultracentrifugal devices for sedimented solute NMR. *J. Biomol. NMR* **54**, 123–127 (2012).

59. A. J. Leeder, L. J. Brown, J. Becker-Baldus, M. Mehler, C. Glaubitz, R. C. D. Brown, Synthesis of isotopically labeled all-trans retinals for DNP-enhanced solid-state NMR studies of retinylidene proteins. *J Labelled Comp Radiopharm* **61**, 922–933 (2018).

60. S. Tang, D. A. Case, Calculation of chemical shift anisotropy in proteins. *J. Biomol. NMR* **51**, 303–312 (2011).

61. T. Zhu, X. He, J. Z. Zhang, Fragment density functional theory calculation of NMR chemical shifts for proteins with implicit solvation. *Phys. Chem.* **14**, 7837–7845 (2012).

62. R. Salomon-Ferrer, A. W. Gotz, D. Poole, S. Le Grand, R. C. Walker, Routine microsecond molecular dynamics simulations with AMBER on GPUs. 2. explicit solvent particle mesh ewald. *J Chem Theory Comput.* **9**, 3878–3888 (2013).

63. D. Boeuf, S. Audic, L. Brillet-Gueguen, C. Caron, C. Jeanthon, MicRhoDE: A curated database for the analysis of microbial rhodopsin diversity and evolution. *Database* **2015**, bav080 (2015).

64. S. Sunagawa, S. G. Acinas, P. Bork, C. Bowler, Tara Oceans Coordinators, S. G. Acinas, M. Babin, P. Bork, E. Boss, C. Bowler, G. Cochrane, C. de Vargas, M. Follows, G. Gorsky, N. Grimsley, L. Guidi, P. Hingamp, D. Iudicone, O. Jaillon, S. Kandels, L. Karp-Boss, E. Karsenti, M. Lescot, F. Not, H. Ogata, Ś.. Pesant, N. Poulton, J. Raes, C. Sardet, M. Sieracki, S. Speich, L. Stemmann, M. B. Sullivan, S. Sunagawa, P. Wincker, D. Eveillard, G. Gorsky, L. Guidi, D. Iudicone, E. Karsenti, F. Lombard, H. Ogata, S. Pesant, M. B. Sullivan, P. Wincker, C. de Vargas, Tara Oceans: Towards global ocean ecosystems biology. *Nat. Rev. Microbiol.* **18**, 428–445 (2020).

65. H. Ashkenazy, E. Erez, E. Martz, T. Pupko, N. Ben-Tal, ConSurf 2010: Calculating evolutionary conservation in sequence and structure of proteins and nucleic acids. *Nucleic Acids Res.* **38**, W529–533 (2010).

66. N.-O. Kyle Carothers, Ocean Explorer, National Oceanic and Atmospheric Administration (2022); oceanexplorer.noaa.gov/explorations/04deepscope/background/deeplight/deeplight.html.

67. K. Inoue, H. Ono, R. Abe-Yoshizumi, S. Yoshizawa, H. Ito, K. Kogure, H. Kandori, A light-driven sodium ion pump in marine bacteria. *Nat. Commun.* **4**, 1678 (2013).

68. N. M. Szeverenyi, M. J. Sullivan, G. E. Maciel, Observation of spin exchange by two-dimensional fourier transform 13C cross polarization-magic-angle spinning. *J. Magn. Reson.* **47**, 462–475 (1982).

69. B. M. Fung, A. K. Khitrin, K. Ermolaev, An improved broadband decoupling sequence for liquid crystals and solids. *J. Magn. Reson.* **142**, 97–101 (2000).

70. J. L. Markley, A. Bax, Y. Arata, C. W. Hilbers, R. Kaptein, B. D. Sykes, P. E. Wright, K. Wüthrich, Recommendations for the presentation of NMR structures of proteins and nucleic acids-(IUPAC Recommendations 1998). *Pure Appl. Chem.* **70**, 117–142 (1998).

71. M. Bak, J. T. Rasmussen, N. C. Nielsen, SIMPSON: A general simulation program for solid-state NMR spectroscopy *J Magn Reson* **213**, 366–400 (2011).

72. M. Bak, J. T. Rasmussen, N. C. Nielsen, SIMPSON: A general simulation program for solid-state NMR spectroscopy. *J. Magn. Reson.* **147**, 296–330 (2000).

73. S. F. Liu, J. D. Mao, K. Schmidt-Rohr, A robust technique for two-dimensional separation of undistorted chemical-shift anisotropy powder patterns in magic-angle-spinning NMR. *J. Magn. Reson.* **155**, 15–28 (2002).

74. N. Pfleger, M. Lorch, A. C. Woerner, S. Shastri, C. Glaubitz, Characterisation of Schiff base and chromophore in green proteorhodopsin by solid-state NMR. *J. Biomol. NMR* **40**, 15–21 (2008).

75. H. J. de Groot, G. S. Harbison, J. Herzfeld, R. G. Griffin, Nuclear magnetic resonance study of the Schiff base in bacteriorhodopsin: Counterion effects on the 15N shift anisotropy. *Biochemistry* **28**, 3346–3353 (1989).

76. A. Bielecki, A. C. Kolbert, M. H. Levitt, Frequency-switched pulse sequences - homonuclear decoupling and dilute spin Nmr in solids. *Chem. Phys. Lett.* **155**, 341–346 (1989).

77. M. Hong, Solid-state dipolar INADEQUATE NMR spectroscopy with a large double-quantum spectral width. *J. Magn. Reson.* **136**, 86–91 (1999).

78. M. Hohwy, H. J. Jakobsen, M. Eden, M. H. Levitt, N. C. Nielsen, Broadband dipolar recoupling in the nuclear magnetic resonance of rotating solids: A compensated C7 pulse sequence. *J. Chem. Phys.* **108**, 2686–2694 (1998).

79. J. M. Walter, D. Greenfield, C. Bustamante, J. Liphardt, Light-powering Escherichia coli with proteorhodopsin. *Proc. Natl. Acad. Sci. U.S.A.* **104**, 2408–2412 (2007).

80. G. S. Harbison, S. O. Smith, J. A. Pardoen, P. P. J. Mulder, J. Lugtenburg, J. Herzfeld, R. Mathies, R. G. Griffin, Solid-state 13C NMR studies of retinal in bacteriorhodopsin. *Biochemistry* **23**, 2662–2667 (1984).

81. S. O. Smith, H. J. M. de Groot, R. Gebhard, J. M. L. Courtin, J. Lugtenburg, J. Herzfeld, R. G. Griffin, Structure and protein environment of the retinal chromophore in light- and dark-adapted bacteriorhodopsin studied by solid-state NMR. *Biochemistry* **28**, 8897–8904 (1989).

82. G. S. Harbison P. P. J. Mulder, H. Pardoen, J. Lugtenburg, J. Herzfeld, R. G. Griffin, High-Resolution C-13 Nmr of retinal derivatives in the solid-state. *J. Am. Chem. Soc.* **107**, 4810–4816 (1985).

83. N. Kubatova, J. Mao, C. E. Eckert, K. Saxena, S. L. Gande, J. Wachtveitl, C. Glaubitz, H. Schwalbe, Light dynamics of the retinal-disease-relevant G90D bovine rhodopsin mutant. *Angew. Chem. Int. Ed. Engl.* **59**, 15656–15664 (2020).

84. L. Shi, M. A. M. Ahmed, W. Zhang, G. Whited, L. S. Brown, V. Ladizhansky, Three-dimensional solid-state nmr study of a seven-helical integral membrane proton pump—structural insights *J. Mol. Biol.* **386**, 1078–1093 (2009),.

85. L. Shi, E. M. Lake, M. A. Ahmed, L. S. Brown, V. Ladizhansky, Solid-state NMR study of proteorhodopsin in the lipid environment: Secondary structure and dynamics. *Biochim. Biophys. Acta* **1788**, 2563–2574 (2009).

86. F. Hempelmann, S. Hölper, M. K. Verhoefen, A. C. Woerner, T. Köhler, S. A. Fiedler, N. Pfleger, J. Wachtveitl, C. Glaubitz, His75-Asp97 cluster in green proteorhodopsin. *J. Am. Chem. Soc.* **133**, 4645–4654 (2011).

87. Y. Shen, A. Bax, SPARTA+: A modest improvement in empirical NMR chemical shift prediction by means of an artificial neural network. *J. Biomol. NMR* **48**, 13–22 (2010).

88. B. Han, Y. Liu, S. W. Ginzinger, D. S. Wishart, SHIFTX2: Significantly improved protein chemical shift prediction. *J. Biomol. NMR* **50**, 43–57 (2011).

89. J. Meiler, PROSHIFT: Protein chemical shift prediction using artificial neural networks. *J. Biomol. NMR* **26**, 25–37 (2003).

90. X. P. Xu, D. A. Case, Automated prediction of 15N, 13Calpha, 13Cbeta and 13C' chemical shifts in proteins using a density functional database. *J. Biomol. NMR* **21**, 321–333 (2001).

91. A. Cavalli, X. Salvatella, C. M. Dobson, M. Vendruscolo, Protein structure determination from NMR chemical shifts. *Proc. Natl. Acad. Sci. U.S.A.* **104**, 9615–9620 (2007).

92. L. Joedicke, J. Mao, G. Kuenze, C. Reinhart, T. Kalavacherla, H. R. A. Jonker, C. Richter, H. Schwalbe, J. Meiler, J. Preu, H. Michel, C. Glaubitz, The molecular basis of subtype selectivity of human kinin G-protein-coupled receptors. *Nat. Chem. Biol.* **14**, 284–290 (2018).

93. Y. Liu, J. Saurí, E. Mevers, M. W. Peczuh, H. Hiemstra, J. Clardy, G. E. Martin, R. T. Williamson, Unequivocal determination of complex molecular structures using anisotropic NMR measurements. *Science* **356**, 7 (2017).

94. N. Nath, J. C. Fuentes-Monteverde, D. Pech-Puch, J. Rodríguez, C. Jiménez, M. Noll, A. Kreiter, M. Reggelin, A. Navarro-Vázquez, C. Griesinger, Relative configuration of micrograms of natural compounds using proton residual chemical shift anisotropy. *Nat. Commun.* **11**, 4372 (2020).

95. C. J. Pickard, E. Salager, G. Pintacuda, B. Elena, L. Emsley, Resolving structures from powders by NMR crystallography using combined proton spin diffusion and plane wave DFT calculations. *J. Am. Chem. Soc.* **129**, 8932–8933 (2007).

96. T. Zhu, J. Z. Zhang, X. He, Correction of erroneously packed protein's side chains in the NMR structure based on ab initio chemical shift calculations. *PCCP* **16**, 18163–18169 (2014).

97. X. S. Jin, T. Zhu, J. Z. H. Zhang, X. He, A systematic study on RNA NMR chemical shift calculation based on the automated fragmentation QM/MM approach. *RSC Adv.* **6**, 108590–108602 (2016).

98. X. Jin, T. Zhu, J. Z. H. Zhang, X. He, Automated fragmentation QM/MM calculation of NMR chemical shifts for protein-ligand complexes. *Front. Chem.* **6**, 150 (2018).

99. M. Shi, X. Jin, Z. Wan, X. He, Automated fragmentation quantum mechanical calculation of (13)C and (1)H chemical shifts in molecular crystals. *J. Chem. Phys.* **154**, 064502 (2021).

100. M. J. Frisch *et al.*, Gaussian 09, revision E. 01. *Gaussian Inc., Wallingford, CT*, (2009).

101. J. R. Cheeseman, G. W. Trucks, T. A. Keith, M. J. Frisch, A comparison of models for calculating nuclear magnetic resonance shielding tensors. *J. Chem. Phys.* **104**, 5497–5509 (1996).

102. U. Haeberlen, High Resolution NMR in Solids: Selective Averaging (Academic, New York). (1976).

103. C. Ji, Y. Mei, J. Z. Zhang, Developing polarized protein-specific charges for protein dynamics: MD free energy calculation of pKa shifts for Asp26/Asp20 in thioredoxin. *Biophys. J.* **95**, 1080–1088 (2008).

104. C. I. Bayly, P. Cieplak, W. Cornell, P. A. Kollman, A well-behaved electrostatic potential based method using charge restraints for deriving atomic charges: The RESP model. *J. Phys. Chem.* **97**, 10269–10280 (2002).

105. W. Rocchia, E. Alexov, B. Honig, Extending the applicability of the nonlinear Poisson-Boltzmann equation: Multiple dielectric constants and multivalent ions. *J. Phys. Chem. B* . **105**, 6507–6514 (2001).

106. A. T. Petkova, M. Hatanaka, C. P. Jaroniec, J. G. Hu, M. Belenky, M. Verhoeven, J. Lugtenburg, R. G. Griffin, J. Herzfeld, Tryptophan interactions in bacteriorhodopsin: A heteronuclear solid-state NMR study. *Biochemistry* **41**, 2429–2437 (2002).

107. M. Martori López, Creation and visualization of a database regarding diversity and distribution of proteorhodopsin-containing bacteria across the whole ocean. http://hdl.handle.net/10230/43935, (2019).
